# 44th National Congress of the Italian Society of Nephrology Nurses (SIAN)

**DOI:** 10.3390/healthcare14203445

**Published:** 2026-10-14

**Authors:** Marco Sguanci, Lea Godino, Anna Grimaldi, Gaetano Ferrara, Giovanni Cangelosi, Sara Morales Palomares, Stefano Mancin, on behalf of the Italian Society of Nephrology Nurses (SIAN) Board of Directors

**Affiliations:** Italian Society of Nephrology Nurses (SIAN), Via Capotesta 1/30, 07026 Olbia, Italyamaranto1984@libero.it (G.F.);

**Keywords:** nephrology nursing, hemodialysis, kidney disease, nursing research, patient-centred care, professional development, healthcare innovation, nursing education

## Abstract

The 44th National Congress of the Italian Society of Nephrology Nurses (SIAN), held in Bologna, Italy, from 4 to 6 May 2026, provided a national forum for discussion on the evolving role of nursing in nephrology. The Congress was preceded by a dedicated pre-congress session focused on education, research, and clinical practice, with particular attention to the involvement of nursing students and early-career professionals in scientific activities. Across plenary sessions, round tables, workshops, oral communications, and digital poster presentations, the scientific programme addressed major areas of contemporary nephrology nursing, including professional development, patient-centred care, vascular access management, dialysis technologies, kidney transplantation, symptom management, home and community care, digital health, artificial intelligence, sustainability, and nutritional care. Particular attention was given to the integration of evidence-based practice, clinical expertise, technological innovation, interprofessional collaboration, and patient education. This Conference Proceedings provides an overview of the scientific and educational activities of the meeting and brings together the accepted contributions presented during both the pre-congress session and the main Congress. Collectively, these contributions illustrate the diversity and continued development of nephrology nursing practice and research in Italy and highlight the importance of scientific societies in promoting professional competence, knowledge exchange, and innovation in kidney care.

## 1. Pre-Congress Session

Title: Education, Research and Clinical Practice: A Dialogue for the Evolution of Nephrology Nursing Care
Location: Bologna, Italy
Dates: 4 May 2026
Event Organizer: Italian Society of Nephrology Nurses (SIAN)

### 1.1. Scientific Committee Chair

Dr. Domenica Gazineo

### 1.2. Scientific Committee

Dr. Mauro Parozzi
Dr. Silvia Cappelletti
Dr. Lea Godino

### 1.3. PCS Introduction

The pre-congress session (PCS) of the 44th National Congress of the Italian Society of Nephrology Nurses (SIAN), entitled “Education, Research and Clinical Practice: A Dialogue for the Evolution of Nephrology Nursing Care”, was designed to promote a structured dialogue between academic education, clinical practice, scientific societies, and publishing stakeholders involved in the advancement of nursing knowledge [1].

Against the backdrop of increasing challenges related to the attractiveness of the nursing profession and the retention of nursing students, the initiative focused on strengthening the scientific education of nursing students and newly graduated nurses. Research was presented as a key driver for professional development and for translating rapidly evolving kidney-care guidance into evidence-informed practice [2,3,4,5,6,7,8,9,10,11]. By providing a dedicated space for the presentation and critical discussion of undergraduate theses and applied research projects, the session aimed to foster methodological competence, critical thinking, and professional awareness among emerging nurses [6].

A central objective of the conference was to enhance integration between university education, research, and clinical practice, while encouraging mentoring, networking, and knowledge transfer across different care settings [3,4,6]. The program also sought to reinforce participants’ sense of belonging to the scientific community and to stimulate interest in nursing research as a means of improving quality of care, supporting innovation, and contributing to the future development of the nursing profession [5,6].

In line with the core values of nursing, the session positioned students and early-career nurses as active contributors to professional growth, clinical improvement and scientific production within nephrology nursing [7,8,9,10,11].

### 1.4. Scientific Contributions

This section includes all accepted scientific contributions presented during the PCS session, including oral communications and digital posters.

**PCS01. Person assisted with urostomy: educational strategies to improve stoma management in the home setting**
Francesca Corinaldesi
Marche University Hospital, Surgical Area Department—Ancona (Italy); francesca.corinaldesi@ospedaliriuniti.marche.it
**Abstract**
*Background/Objectives*: Urostomy involves the external diversion of the urinary tract through a stoma and is performed when bladder function can no longer be preserved. This condition may negatively affect patients’ psychosocial well-being, body image, interpersonal relationships, and quality of life. Adaptation to life with a stoma requires the acquisition of self-care skills and competencies. Nurses play a key role in supporting patients through targeted educational interventions aimed at improving stoma self-management in the home setting. This literature review aimed to identify evidence-based educational strategies and best nursing practices to enhance self-care in adults with urostomy. *Methods*: A literature review was conducted using PubMed, Embase, and CINAHL databases. The search strategy combined the keywords “ostom*,” “ostomy patients,” “urolog*,” “educat*,” “support,” “intervention,” “strateg*,” and “nurs*” with Boolean operators AND/OR. The research question was structured according to the PIO framework: Population = discharged patients with urostomy; Intervention = nursing intervention; Outcome = improved self-care. Studies involving adult urostomy patients, published in English or Italian, were included. Peer reviews and conference proceedings were excluded. *Results*: Thirty-two studies met the inclusion criteria. Findings highlighted the importance of preoperative education, psychological support, validated assessment tools, and structured self-care training in improving stoma management and quality of life. Educational interventions positively influenced anxiety reduction, adaptation, body image perception, and self-management abilities. *Conclusions*: Structured nursing education, initiated preoperatively and continued after discharge, improves self-care skills and adaptation in urostomy patients. Implementing evidence-based educational programs may enhance patient outcomes and quality of life.
**Keywords:** urostomy; educational intervention; nurse; adult ostomy patients; strategies

**PCS02. Quality of Information on Kidney Transplantation in Social Media: A Cross-Platform Analysis and Identification of Red Flags**
Emmanuele Stefanini ^1^, Serena Ludovica Filomena Caparrotti ^1^, Antonio De Biase ^1^, Domenico Di Sivo ^1^, Salvatore Orabona ^1^, Orsola Agorini ^2^, Adolfo Gallo ^2^, Francesco Rinaldi ^2^, Anna Iavarone ^3^, Gianluca Marino ^2^, Anna Grimaldi ^2,^* and Giuseppe de Falco ^1^

1. University of Campania “Luigi Vanvitelli”, AO dei Colli Teaching Center, Via Leonardo Bianchi, 80131, Naples, Italy
2. University of Campania “Luigi Vanvitelli”, Aversa Teaching Center (ASL Caserta), Via Santa Lucia, 81031 Aversa, Caserta, Italy
3. Pegaso Telematic University, Naples, Italy

* Correspondence: anna.grimaldi92a@gmail.com
**Abstract**
*Background/Objectives*: Social media are an increasingly important source of health information, although content quality is heterogeneous and often unregulated. In kidney transplantation, inaccurate information may negatively influence treatment adherence and follow-up. This study aims to evaluate the quality of information related to kidney transplantation and to identify the presence of red flags. *Methods*: A cross-sectional content analysis was conducted on Italian-language content published on Instagram, TikTok, and YouTube. Information quality was assessed using the EQIP score. Predefined red flags were identified. Quantitative variables were expressed as median [IQR] and mean ± SD. Differences between platforms were analyzed using the Kruskal–Wallis test with post-hoc analysis (Mann–Whitney test with Bonferroni correction). *Results*: A total of 228 contents were included. Overall information quality was moderate, with significant differences across platforms. YouTube showed higher quality scores compared to Instagram and TikTok, while TikTok scored higher than Instagram. At least one red flag was identified in a minority of contents, mainly related to minimization of follow-up, unrealistic expectations, and unsupervised treatment changes. The prevalence of red flags was higher on Instagram and TikTok compared to YouTube. *Conclusions*: The quality of information on kidney transplantation varies significantly across social media platforms, with higher quality observed in more structured content. The presence of red flags highlights relevant issues in digital health communication. Strategies are needed to promote evidence-based content and improve health literacy.
**Keywords:** kidney transplantation; social media; information quality.

**PCS03. The Caring Relationship in Dialysis: A Comparative Analysis Between Two Centers in Italy**

1. Chiara Fanelli ^1,^*, Maria Francisca Navajas Martinez ^2^, Livia Nazzaro ^2^ and Sabrina Saccoccia ^2,3^
2. Bachelor’s Degree in Nursing, Università Campus Bio-Medico di Roma, Via Álvaro del Portillo 21, 00128 Rome, Italy
3. Nephrology Unit, Fondazione Policlinico Universitario Campus Bio-Medico, Via Álvaro del Portillo 200, 00128 Rome, Italy
4. Department of Biomedicine and Prevention, University of Rome Tor Vergata, Via Montpellier 1, 00133 Rome, Italy

* Correspondence: chiara.fanelli@alcampus.it; fanellic28@gmail.com
**Abstract**
*Background/Objectives*: End-stage renal disease requires continuous dialysis treatment, profoundly affecting patients’ quality of life. Hemodialysis generates over 600 h per year of nurse-patient contact, making the therapeutic relationship a key clinical element. Depression affects 20–40% of dialysis patients, increasing mortality risk by 51%. This study aimed to analyse patients’ perceptions of nursing care quality in two Italian dialysis centers, comparing the relational role of nurses in a provincial versus an urban setting. *Methods*: A cross-sectional observational study was conducted using a structured questionnaire (27 items, Likert scale 1–5 plus open-ended questions), administered between October 2025 and February 2026 via Google Forms. The sample included 130 adult patients (≥18 years, on dialysis ≥3 months): 60 from the “Città di Maglie” Centre (Lecce) and 70 from the “Nuova Itor” facility (Rome). Domains assessed included technical competence, communication quality, emotional support, care continuity, dignity and privacy, and psychological well-being. *Results*: Overall, 78% of patients rated nurses’ technical competence as very high and 90% reported feeling listened to always or often. However, more than 80% reported difficulties understanding information provided, a finding common to both centres. The Lecce centre showed greater personalisation of care and relational continuity, while the Rome facility was characterised by higher standardisation of procedures and availability of specialist services. *Conclusions*: Person-centred dialysis care requires integration of technical skills and relational competences. Communication emerged as the main critical area regardless of context, underscoring the need for targeted training in health literacy. Adopting the Primary Nursing model, integrating psychologists into multidisciplinary teams, and reducing nursing turnover are recommended strategies to enhance the therapeutic relationship and patient outcomes.
**Keywords:** hemodialysis; therapeutic relationship; nursing care; communication; care continuity

**PCS04. eHealth Literacy and Professional Self-Efficacy among Nursing Students: A Multicenter Observational Study on the Use of Digital Resources in Nephrology**
Adolfo Gallo ^1^, Orsola Agorini ^1^, Francesco Rinaldi ^1^, Giuseppe De Falco ^2^, Antonio De Biase ^2^, Emmanuele Stefanini ^2^, Serena Ludovica Filomena Caparrotti ^2^, Domenico Di Sivo ^2^, Salvatore Orabona ^2^, Anna Iavarone ^3^, Anna Grimaldi ^1^ and Gianluca Marino ^1,^*

1. University of Campania “Luigi Vanvitelli”, Aversa Teaching Center (ASL Caserta), Via Santa Lucia, 81031 Aversa, Caserta, Italy
2. University of Campania “Luigi Vanvitelli”, AO dei Colli Teaching Center, Via Leonardo Bianchi 80131, Naples, Italy
3. Pegaso Telematic University, Naples, Italy

* Correspondence: gianluca.marino1@unicampania.it
**Abstract**
*Background/Objectives*: The increasing availability of digital health resources requires nursing students to develop adequate eHealth literacy, defined as the ability to seek, understand, and use online health information. At the same time, professional self-efficacy is a key determinant of nursing competence development and quality of care. In nephrology, this relationship remains underexplored. The aim of this study is to evaluate the association between eHealth literacy and professional self-efficacy among nursing students. *Methods*: A multicenter cross-sectional observational study was conducted and reported according to STROBE recommendations. A total of 50 questionnaires were collected; 48 with complete responses to validated scales were included in the analysis. eHealth literacy was assessed using the I-eHEALS (8 items), and self-efficacy using the NPSES2 (10 items). Analyses included descriptive statistics, Pearson and Spearman correlations, and simple linear regression. *Results*: Mean levels of eHealth literacy and self-efficacy were overall good. A positive relationship emerged between eHealth literacy and self-efficacy, supported by consistent analytical findings. No meaningful differences were observed based on the use of digital resources, educational setting, or time spent using digital tools. *Conclusions*: In this sample, higher eHealth literacy is associated with greater nursing self-efficacy. However, the strength of the association is moderate, and findings should be interpreted cautiously due to the limited sample size and cross-sectional design. Further studies with larger samples are needed to confirm these results.
**Keywords:** eHealth literacy; self-efficacy; nursing students

**PCS05. Interprofessional Collaboration in Nephrology Clinical Placements: Nursing Students’ Perceptions in a Multicenter Study Using the I-AITCS II**
Antonio De Biase ^1^, Stefanini Emmanuele ^1^, Serena Ludovica Filomena Caparrotti ^1^, Domenico Di Sivo ^1^, Salvatore Orabona ^1^, Orsola Agorini ^2^, Adolfo Gallo ^2^, Francesco Rinaldi ^2^, Anna Iavarone ^3^, Gianluca Marino ^2^, Anna Grimaldi ^2^ and Giuseppe de Falco ^1,^*

1. University of Campania “Luigi Vanvitelli”, AO dei Colli Teaching Center, Via Leonardo Bianchi, 80131, Naples, Italy
2. University of Campania “Luigi Vanvitelli”, Aversa Teaching Center (ASL Caserta), Via Santa Lucia, 81031, Aversa, Caserta, Italy
3. Pegaso Telematic University, Naples, Italy

* Correspondence: giuseppe.defalco@ospedalideicolli.it
**Abstract**
*Background/Objectives:* The management of nephrology patients requires a high level of integration among healthcare professionals. Interprofessional collaboration is a key determinant of care quality and patient safety. Nursing students’ perceptions during clinical placements represent an important indicator of the effectiveness of educational environments. This study aims to describe the level of interprofessional collaboration perceived by nursing students during clinical placements in nephrology settings. *Methods:* A cross-sectional observational study was conducted among 48 nursing students from different universities (University of Campania “L. Vanvitelli”—Aversa and AO Monaldi teaching centers—and Parthenope University). Interprofessional collaboration was assessed using the validated I-AITCS II scale (23 items, Likert 1–5), covering partnership, cooperation, and coordination. Socio-demographic and educational variables were collected. Statistical analysis included descriptive statistics (mean ± SD, frequencies, percentages) and internal consistency assessment using Cronbach’s alpha. *Results*: The sample was predominantly female, under 25 years of age, and enrolled in the third year. Most students had clinical placements in hospital hemodialysis units. The mean I-AITCS II score was 4.08 ± 0.64, with 65% reporting high levels of collaboration. Internal consistency was high (α = 0.97). Overall responses were positive, with minor critical issues in cooperation and conflict management. *Conclusions*: Perceived interprofessional collaboration among nursing students in nephrology settings is overall high. However, some critical aspects remain in cooperation and coordination, highlighting the need to strengthen collaborative dynamics in both clinical and educational contexts.
**Keywords:** interprofessional collaboration; nursing students; clinical placement.

**PCS06. Assessment tools for chronic kidney disease–associated pruritus: a scoping review**
Freddy Elias Lopez Medina ^1^, Sofia Matteucci ^2,^*, Anna Grimaldi ^3^, Sara Morales Palomares ^3,4^, Stefano Mancin ^2^, Daniela Cattani ^2^, Alessandra Dacomi ^2^, Giuseppina Tomaiuolo ^2^, Diego Lopane ^2^ and Beatrice Mazzoleni ^5^

1. Humanitas University, Via Rita Levi Montalcini 4, 20090 Pieve Emanuele, Milan, Italy
2. IRCCS Humanitas Research Hospital, via Manzoni 56, 20089, Rozzano, Milan, Italy
3. Italian Society of Nephrology Nurses (SIAN), Via Capotesta 1/30, 07026 Olbia, Italy
4. Department of Pharmacy, Health and Nutritional Sciences (DFSSN), University of Calabria, Rende, Cosenza, Italy
5. Department of Biomedical Science Humanitas University, Via Rita Levi Montalcini 4, Milan, Pieve Emanuele, 20090, Italy

* Correspondence: sofia.matteucci@humanitas.it
**Abstract**
*Background/Objectives:* Chronic kidney disease–associated pruritus (CKD-aP) is a common and burdensome complication, particularly among patients undergoing dialysis, yet it remains under-recognized. The wide range of available assessment tools may limit consistent evaluation and clinical management. This scoping review aims to identify and summarize validated instruments used to assess pruritus in adults with CKD, focusing on their characteristics and measurement domains. *Methods*: This scoping review was conducted following the Joanna Briggs Institute methodology and reported according to PRISMA-ScR guidelines. Search was performed in PubMed/MEDLINE, Embase, CINAHL, and the Cochrane Library. Primary studies evaluating validated tools for pruritus assessment in adult CKD populations were included. Study selection and data extraction were independently performed by two reviewers. Results were synthesized using a narrative descriptive approach. *Results*: Out of 992 records identified, 10 studies published between 2017 and 2025 were included. Considerable heterogeneity was observed in study design and geographical distribution. Identified tools included unidimensional scales, primarily assessing symptom intensity, and multidimensional instruments evaluating additional domains such as duration, functional impact, and quality of life. Most tools demonstrated acceptable psychometric properties, although differences emerged in validation methods, application contexts, and measured dimensions. *Conclusions:* Substantial variability exists in the tools used to assess CKD-aP. While simpler scales may support rapid screening, multidimensional instruments allow a broader evaluation of patient experience. Wider adoption of validated tools may improve assessment and support more consistent clinical management.
**Keywords:** chronic kidney disease; CKD-aP; assessment tools.

**PCS07. Dialysis as a High Cognitive Load Environment: Nursing Students’ Perception Using NASA-TLX in a Paper-Based Scenario**
Francesco Rinaldi ^1^, Adolfo Gallo ^1^, Orsola Agorini ^1^, Giuseppe De Falco ^2^, Antonio De Biase ^2^, Emmanuele Stefanini ^2^, Serena Ludovica Filomena Caparrotti ^2^, Domenico Di Sivo ^2^, Salvatore Orabona ^2^, Anna Iavarone ^3^, Anna Grimaldi ^1,^* and Gianluca Marino ^1^

1. University of Campania “Luigi Vanvitelli”, Aversa Teaching Center (ASL Caserta), Via Santa Lucia, 81031, Aversa, Caserta, Italy
2. University of Campania “Luigi Vanvitelli”, AO dei Colli Teaching Center, Via Leonardo Bianchi, 80131, Naples, Italy
3. Pegaso Telematic University, Naples, Italy

* Correspondence: anna.grimaldi92a@gmail.com
**Abstract**
*Background/Objectives:* Dialysis is a complex care setting characterized by the simultaneous management of clinical parameters, technological devices, and communication with chronic patients. In educational contexts, these conditions may increase cognitive workload, potentially affecting decision-making processes and patient safety. The NASA Task Load Index (NASA-TLX) assesses perceived workload, while situational anxiety can be measured using the STAI-Y. This study aims to quantify cognitive workload in a simulated dialysis scenario and to analyze its association with anxiety and self-efficacy. *Methods*: A cross-sectional observational study was conducted using a standardized written clinical scenario involving the management of a complex dialysis session. Nursing students were recruited during educational activities. Instruments included NASA-TLX (Raw), STAI-Y1, and a visual analog scale (VAS) for self-efficacy. Variables were described using mean ± standard deviation and median. Normality was assessed with the Shapiro–Wilk test. Spearman correlations and internal consistency (Cronbach’s α) were calculated. *Results*: A total of 47 third-year nursing students were included. Cognitive workload was perceived as high. Anxiety levels were moderate, while self-efficacy was relatively good. A positive association emerged between workload and anxiety, whereas no relationship was observed with self-efficacy. The NASA-TLX demonstrated good internal consistency. *Conclusions*: The dialysis scenario is perceived as highly cognitively demanding, with a significant emotional impact. Temporal and mental demands are the main contributors to workload. Standardized scenarios may help identify cognitive challenges and support targeted educational interventions.
**Keywords:** dialysis; cognitive workload; nursing students.

**PCS08. Patient Safety and Risk Management in Home-Based Nephrology Care: Implications for Community Nursing. A Scoping Review**
Francesco Rinaldi ^1^, Adolfo Gallo ^1^, Orsola Agorini ^1^, Marino Gennaro ^2^, Amato Domenico ^3^, Anna Iavarone ^4^, Anna Grimaldi ^1,4^ and Gianluca Marino ^1,4,^*

1. University of Campania “Luigi Vanvitelli”, Aversa Teaching Center (ASL Caserta), Via Santa Lucia, 81031, Aversa, Caserta, Italy
2. Local Health Authority (ASL) Caserta, District 17, Via Santa Lucia, 81031, Aversa, Caserta, Italy
3. Local Health Authority (ASL) Caserta, District 13, Via Caudina, 81024, Maddaloni (CE), Italy
4. Pegaso Telematic University, Naples, Italy

* Correspondence: gianluca.marino1@unicampania.it
**Abstract**
*Background/Objectives:* Patients with chronic kidney disease receiving home-based care present high levels of frailty and multimorbidity, with an increased risk of adverse events. In this context, patient safety is an emerging priority, and the role of the community nurse is central in clinical risk management. This study aims to map the available evidence on the impact of community nursing care in promoting patient safety and preventing adverse events in home-based nephrology patients. *Methods*: The review was conducted according to PRISMA-ScR guidelines. The search was performed across four electronic databases (PubMed, CINAHL, Scopus, and Web of Science) and complemented with grey literature, including Google Scholar and international organization websites (WHO, KDIGO, ISPD), up to March 2026. Studies involving adult patients with chronic kidney disease receiving home or community care, focusing on patient safety and nursing interventions, were included. A total of 1285 records were identified; after duplicate removal, 965 were screened. Eighty-five full-text articles were assessed, with 29 studies included in the final analysis. *Results*: Community nursing care was associated with a reduction in adverse events, including medication errors, infections, and fluid overload, improving clinical monitoring and continuity of care. Key interventions included therapeutic education, caregiver involvement, and frailty assessment. However, challenges remain related to organizational variability and limited standardization of risk assessment tools. *Conclusions:* Community nurses play a central role in ensuring patient safety in home-based nephrology care. Further research is needed to develop standardized care models and risk management tools.
**Keywords:** chronic kidney disease; patient safety; community nursing

**PCS09. The First Clinical Placement in Dialysis: Emotional Experience and Professional Identity Development among Nursing Students**
Orsola Agorini ^1^, Francesco Rinaldi ^1^, Adolfo Gallo ^1^, Giuseppe De Falco ^2^, Antonio De Biase ^2^, Emmanuele Stefanini ^2^, Serena Ludovica Filomena Caparrotti ^2^, Domenico Di Sivo ^2^, Salvatore Orabona ^2^, Anna Iavarone ^3^, Anna Grimaldi ^1^ and Gianluca Marino ^1,3^

1. University of Campania “Luigi Vanvitelli”, Aversa Teaching Center (ASL Caserta), Via Santa Lucia, 81031, Aversa, Caserta, Italy
2. University of Campania “Luigi Vanvitelli”, AO dei Colli Teaching Center, Via Leonardo Bianchi, 80131, Naples, Italy
3. Pegaso Telematic University, Naples, Italy

* Correspondence: anna.grimaldi92a@gmail.com
**Abstract**
*Background/Objectives*: The first clinical placement is crucial in the development of nursing students’ professional identity and self-efficacy. Complex settings such as dialysis, characterized by high technological demands and continuous relationships with chronic patients, may generate intense emotional experiences and influence career orientation. The descriptive phenomenological approach according to Giorgi allows exploration of the lived meaning of such experiences. The aim of this study is to describe and understand the emotional and professional experiences of students during their first clinical placement in dialysis. *Methods*: A qualitative phenomenological study was conducted following Giorgi’s approach. Thirty-six students (second–third year) undertaking their first clinical placement in dialysis were included. Data were collected through individual audio-recorded semi-structured interviews. Analysis followed Giorgi’s steps: holistic reading, identification of meaning units, transformation, and synthesis into themes and essential structure. Methodological rigor was ensured through audit trail and researcher triangulation. *Results:* Several themes emerged. Students reported initial disorientation and challenges related to technology, followed by progressive adaptation and learning. Emotional experiences evolved from insecurity to more positive feelings. Support from the healthcare team and relationships with chronic patients facilitated learning and empathy. Growth involved increased competencies, self-efficacy, and development of professional identity, consistent with existing literature. Educational needs included stronger theoretical preparation, greater practical involvement, and the use of simulation and structured briefing. *Conclusions*: Clinical placement in dialysis represents a complex yet meaningful learning experience. The implementation of structured briefing, simulation, and emotional debriefing may facilitate student adaptation and support the development of professional identity.
**Keywords:** nursing students; dialysis; professional identity

**PCS10. Telemonitoring in Home Peritoneal Dialysis: Impact on Nursing Care and Clinical Outcomes. A Scoping Review**
Orsola Agorini ^1^, Adolfo Gallo ^1^, Francesco Rinaldi ^1^, Marino Gennaro ^2^, Amato Domenico ^3^, Anna Iavarone ^4^, Anna Grimaldi ^1^ and Gianluca Marino ^1,^*

1. University of Campania “Luigi Vanvitelli”, Aversa Teaching Center (ASL Caserta), Via Santa Lucia, 81031, Aversa, Caserta, Italy
2. Local Health Authority (ASL) Caserta, District 17, Via Santa Lucia, 81031, Aversa, Caserta, Italy
3. Local Health Authority (ASL) Caserta, District 13, Via Caudina, 81024, Maddaloni, Caserta, Italy
4. Pegaso Telematic University, Naples, Italy

* Correspondence: gianluca.marino1@unicampania.it
**Abstract**
*Background/Objectives:* Home peritoneal dialysis is an established strategy in the management of advanced chronic kidney disease. The integration of telemonitoring has introduced new care models potentially capable of improving clinical outcomes and redefining the nursing role, although evidence remains heterogeneous. The aim of this study is to map the available evidence on the impact of telemonitoring in home peritoneal dialysis on clinical outcomes and nursing care. *Methods*: The search was conducted across four electronic databases (PubMed, CINAHL, Scopus, and Web of Science) and complemented by grey literature, including Google Scholar and international scientific society websites (ISPD, KDIGO, ERA), up to March 2026. Studies involving adult patients undergoing home peritoneal dialysis with telemonitoring were included. A total of 1103 records were identified; after duplicate removal, 589 were screened. Eighty-seven full-text articles were assessed, with 36 studies included in the final analysis. *Results*: Telemonitoring was associated with reduced hospitalizations, improved treatment adherence, and earlier management of complications, with a potential reduction in peritonitis. From a nursing perspective, a shift toward more proactive care models emerged, characterized by continuous monitoring, data interpretation, and increased decision-making autonomy. However, challenges persist due to system heterogeneity and the need for specific training. *Conclusions*: Telemonitoring represents a relevant innovation in home peritoneal dialysis, improving outcomes and redefining the nursing role. Further studies are needed to standardize care models and evaluate long-term effectiveness.
**Keywords:** peritoneal dialysis; telemonitoring; nursing care

**PCS11. Non-Pharmacological Nursing Interventions for the Management of Uremic Pruritus in Hemodialysis Patients: A Systematic Review**
Alessandro Armata ^1,^*, Mariaelena Federici ^2^ and Simone Stevanin ^3^

1. Nursing Home Anni d’Oro; Melilli, Siracusa, Italy
2. Undergraduate Student, Bachelor of Science in Nursing Program, University of Padova, Padova, Italy
3. Bachelor of Science in Nursing Program, University Hospital of Padova, University of Padova, Padova, Italy

* Correspondence: animamates02@gmail.com
**Abstract**
*Background/Objectives:* Uremic pruritus, also referred to in the literature as chronic kidney disease-associated pruritus (CKD-aP), affects 40–50% of patients on hemodialysis, significantly compromising sleep quality, psychological well-being, and overall quality of life. Available pharmacological therapies carry relevant risk profiles in this population. Identifying effective and safe non-pharmacological nursing interventions therefore represents a clinical and care priority. *Methods*: A systematic review was conducted following CRD (2008) guidelines and the PRISMA Statement. The review question was structured according to the PIO framework: adult hemodialysis patients with CKD-aP (P), non-pharmacological nursing interventions (I), and reduction in pruritus intensity (O). Five databases were searched: PubMed, CINAHL, Cochrane Library, Scopus, and Google Scholar (English language, 2010–2024). Of 65 identified records, 8 were included following title, abstract, and full-text screening. *Results*: The 8 included studies—4 clinical studies, 2 cross-sectional studies, 1 experimental trial, and 1 quasi-experimental study—involved 751 adults with chronic kidney disease on hemodialysis. Four intervention categories were identified: auricular acupressure, narrowband ultraviolet B phototherapy (NB-UVB), topical herbal therapy (aromatherapy with violet essential oil), and dietary counseling on phosphorus restriction. All interventions yielded a statistically significant reduction in pruritus intensity (VAS). Dietary counseling additionally demonstrated positive effects on serum phosphorus levels and on inflammatory markers, including hs-CRP and IL-2. *Conclusions*: The non-pharmacological nursing interventions examined appear effective in reducing CKD-aP, acting on both symptom intensity and the patient’s biochemical and inflammatory profile, with a favorable safety profile. Nursing emerges as a central professional role in delivering interventions readily integrable into daily clinical practice. All included studies were conducted in non-European settings; further Italian and European studies with adequate sample sizes are warranted to strengthen the evidence base for advanced nephrology nursing practice.
**Keywords:** uremic pruritus; hemodialysis; non-pharmacological nursing interventions; CKD-associated pruritus; systematic review.

**PCS12. Dysphagia in chronic kidney disease: prevalence, underlying mechanisms, and clinical implications: a scoping review**
Donika Qoshi ^1^, Sofia Matteucci ^2^, Anna Grimaldi ^3^, Sara Morales Palomares ^3,4^, Stefano Mancin ^2,^*, Daniela Cattani ^2^, Alessandra Dacomi ^2^, Giuseppina Tomaiuolo ^2^, Diego Lopane ^2^ and Beatrice Mazzoleni ^5^

1. Humanitas University, Via Rita Levi Montalcini 4, 20090 Pieve Emanuele, Milan, Italy
2. IRCCS Humanitas Research Hospital, via Manzoni 56, 20089, Rozzano, Milan, Italy
3. Italian Society of Nephrology Nurses (SIAN), Via Capotesta 1/30, 07026 Olbia, Italy
4. Department of Pharmacy, Health and Nutritional Sciences (DFSSN), University of Calabria, Rende, Italy
5. Department of Biomedical Science Humanitas University, Via Rita Levi Montalcini 4, 20090 Milan, Pieve Emanuele, Italy

* Correspondence: stefano.mancin@humanitas.it
**Abstract**
*Background/Objectives:* Chronic kidney disease (CKD) affects more than 10% of the adult population worldwide; dysphagia represents a clinically relevant but frequently under-recognized manifestation, with potential consequences for nutritional status, hydration, swallowing safety, and quality of life. This scoping review aims to map and synthesize the available evidence on the relationship between dysphagia and CKD. *Methods*: The review was conducted in accordance with the Joanna Briggs Institute framework and reported following PRISMA-ScR guidelines. A comprehensive search was performed in PubMed/MEDLINE, Embase, CINAHL, Cochrane Library, Scopus, and Web of Science, including grey literature. Study selection and data extraction were independently performed by two reviewers. *Results*: Out of 908 records initially identified, 14 studies were included. The included studies involved diverse patient groups across different stages of CKD and care settings. The mean prevalence of dysphagia based on clinical assessment was approximately 16%. Instrumental evaluations frequently identified swallowing alterations, with a substantial proportion of patients presenting features suggestive of impaired swallowing safety. Dysphagia was commonly associated with uremic sarcopenia, protein–energy malnutrition, and oral frailty. Proposed mechanisms included uremic neuro-myopathy, toxin accumulation, gut dysbiosis, and impaired gastrointestinal motility. **Conclusions:** Dysphagia is a frequent but often under-recognized condition in CKD. Its association with sarcopenia and malnutrition may worsen clinical outcomes. Further studies are needed to better define its impact.
**Keywords:** chronic kidney disease; dysphagia; sarcopenia

**PCS13. Nutritional management in adults with end-stage kidney disease undergoing dialysis**
Denise Cucciniello ^1^ and Marco Sguanci ^1,^*, Diego Lopane ^2^, Giovanni Cangelosi ^1,3^, Sofia Matteucci ^2^, Anna Grimaldi ^1^, Gaetano Ferrara ^1^, Sara Morales Palomares ^1,4^, Beatrice Mazzoleni ^5^ and Stefano Mancin ^1^

1. Italian Society of Nephrology Nurses (SIAN), Via Capotesta 1/30, 07026 Olbia, Italy
2. IRCCS Humanitas Research Hospital, via Manzoni 56, 20089, Rozzano, Milano Italy
3. School of Pharmacy, Polo Medicina Sperimentale e Sanità Pubblica “Stefania Scuri”, Università di Camerino, Via Madonna delle Carceri 9, 62032 Camerino, Italy
4. Department of Pharmacy, Health and Nutritional Sciences (DFSSN), University of Calabria, Rende, Italy
5. Department of Biomedical Science Humanitas University, Via Rita Levi Montalcini 4, Milan, Pieve Emanuele, 20090, Italy

* Correspondence: sguancim@gmail.com
**Abstract**
*Background/Objectives*: Nutritional therapy is a cornerstone in the management of adults with end-stage kidney disease (ESKD) receiving hemodialysis or peritoneal dialysis. However, the coexistence of multiple guidance documents may contribute to variability in clinical practice and patient education. This study aimed to systematically review and synthesize available evidence and recommendations on nutritional management in adults with ESKD on dialysis. *Methods:* The review was conducted and reported in accordance with PRISMA guidelines, including documents published between 2015 and 2025. Searches were performed in PubMed, Scopus, Embase, CINAHL, and the Cochrane Library, and complemented by guideline repositories and grey literature. Methodological quality was assessed using the AGREE II instrument. *Results:* A total of 11 records were included. Most documents demonstrated high methodological quality, whereas institutional documents scored lower. Recommendations consistently emphasized the prevention of protein–energy wasting through adequate energy intake and protein intake, with adjustments based on dialysis modality and patient condition. Additional key elements included individualized electrolyte and fluid management, as well as structured patient education with progressive intensification of nutritional support. *Conclusions:* Recommendations across documents were largely consistent. Harmonization of key nutritional targets and implementation strategies may enhance nurse-led education and improve patient adherence in clinical practice.
**Keywords:** dialysis; clinical practice guidelines; nutrition therapy

## 2. Conference Information

Title: Prevention, Care and Innovation: New Perspectives in Nephrology Nursing
Location: Bologna, Italy
Dates: 4–6 May 2026
Event Organizer: Italian Society of Nephrology Nurses (SIAN)

### 2.1. Scientific Committee

#### 2.1.1. Congress President

Dr. Stefano Mancin

#### 2.1.2. Scientific Committee Chair

Dr. Domenica Gazineo

#### 2.1.3. Congress Scientific Committee

Dr. Francesco Barci
Dr. Silvia Cappelletti
Dr. Lea Godino
Dr. Angela Greco
Dr. Addolorata Palmisano
Dr. Francesco Pastore
Dr. Alessandro Pizzo
Dr. Marco Sguanci

### 2.2. Organizing Committee

Promoter: Italian Society of Nephrology Nurses (SIAN)

Professional Congress Organizer and ECM Provider: My Meeting S.r.l.

SIAN Executive Board: Stefano Mancin; Domenica Gazineo; Silvia Cappelletti; Francesco Barci; Angela Greco; Addolorata Palmisano; Alessandro Pizzo.

### 2.3. Conference Introduction

The 44th National Congress of the Italian Society of Nephrology Nurses (SIAN), entitled “Prevention, Care and Innovation: New Perspectives in Nephrology Nursing”, was held in Bologna (Italy) from 4 to 6 May 2026. The congress was designed as a multidisciplinary forum dedicated to the evolution of nephrology nursing, with a specific focus on professional responsibility, innovation, integration, and the development of new care models for people with kidney disease [12,13,14,15].

The scientific programme addressed the transformation of contemporary nephrology through plenary sessions, round tables, simulation laboratories, corporate symposia, oral communications, and digital poster presentations. The opening day focused on the evolution of the nursing role in nephrology, new competencies, health promotion, professional communication, and multiprofessional integration within care pathways [16,17,18].

The congress also explored sustainability in nephrology care, including green dialysis and environmental impact reduction [19,20,21,22], as well as technological innovation in dialysis, continuous renal replacement therapy, vascular access, home and community care, and digital solutions [23,24,25,26]. The programme combined scientific evidence, clinical practice, and interactive learning, supporting a nursing culture able to integrate technical competence with environmental responsibility and interprofessional collaboration [19,20,21,22,27].

The final day focused on emerging professional models in nephrology, humanities, legal and clinical governance, peritoneal dialysis, and nephrology nutrition. Particular emphasis was placed on patient autonomy, personalized care, therapeutic education, and the translation of evidence into practice, themes also reflected in contemporary literature on peritoneal dialysis training and kidney self-management [28,29,30].

Overall, the congress session aimed to serve as a laboratory for discussion and knowledge exchange, where theory and practice meet and where nurses, clinicians, researchers, institutions, and industry partners can contribute to advancing quality, safety, and innovation in nephrology nursing [21,22,27].

### 2.4. Scientific Contributions

This section includes all accepted scientific contributions (SCs) presented during the 44th National Congress of SIAN, including oral communications and digital posters.

The complete scientific program of the 44th National Congress, including the pre-congress session (PCS) and scientific contributions (SCs), is provided in File S1.

**SC01. Care and management of patients undergoing AVF surgery: monitoring of vascular access in the post-operative period**
Ruggiero Ciannarella ^1^, Giuseppe Civetta ^2^, Giuseppe Petruzzelli ^3^, Grazia Petruzzelli ^4^, Maria Luisa Biga ^5^, Pasquale Vincenzo Pietro ^6^, Giuseppe Quacquarelli ^7^, Ruggiero Lemma ^8^, Raffaella Pischetola ^9^ and Tiziana Piccolo ^10^

1. Domus Lauretana, Trinitapoli, Italy
2. U.O.C. Oncologia, P.O. Barletta, ASL BT, Italy
3. Centro Dialisi, P.O. Barletta, ASL BT, Italy
4. NephroCare, Minervino, Italy
5. U.O.C. Nefrologia e Dialisi, ASL BAT, Italy
6. Centro Dialisi, P.O. Barletta, ASL BT, Italy
7. Centro Dialisi, P.O. Barletta, ASL BT, Italy
8. Centro Dialisi, P.O. Barletta, ASL BT, Italy
9. Centro Dialisi, P.O. Barletta, ASL BT, Italy
10. U.O.C. Nefrologia e Dialisi, ASL BAT, Italy

* Correspondence: ruggierociannarella04@gmail.com
**Abstract**
*Background/Objectives:* This study aims to evaluate the usefulness of ultrasound as a tool for vascular access mapping, and therefore for assessing vessel diameter and depth, as well as the level of maturation of the vascular access itself. Six weeks after its creation, the vascular access should have a Qa >600 mL/minute, a diameter >6 mm, and a depth no greater than 6 mm. *Methods*: The study was conducted at the Dialysis Center of the Barletta Hospital, where a sample of seven patients underwent vascular access creation via arteriovenous fistula surgery between January and May 2025. The patients were monitored 1, 4, and 6 weeks after the procedure, in accordance with the most recent guidelines. A surveillance form was completed, reporting associated pathologies, the type of fistula, and any use of anticoagulants or antiplatelet drugs. Transverse and longitudinal images were also acquired using a high-frequency probe under ultrasound control performed by nursing staff. After parameter collection, a statistical analysis of the vascular accesses was performed, and in cases of failed maturation, the etiology related to the underlying disease was assessed. *Results*: The study highlighted that postoperative ultrasound allows for the assessment of vascular access maturity. *Conclusions*: The use of ultrasound allows for monitoring the maturation process of the fistula and avoiding early punctures when it is not yet mature. It is therefore confirmed as a fundamental tool for monitoring arteriovenous fistulas, improving the safety of hemodialysis treatment and the management of vascular accesses.
**Keywords:** doppler ultrasound; arteriovenous fistula; maturation of vascular access

**SC02. Assessment of Quality of Life in Hemodialysis Patients with Uremic Pruritus**
Selene La Legname ^1,^*, Vincenza Giordano ^2^, Chiara Palazzo ^3^, Michele Virgolesi ^2^, Francesco Ricciardi ^4^, Antonio Caputo ^5^, Rita Nocerino ^6^, Michele Strazzulli ^7^, Assunta Guillari ^8^ and Teresa Rea ^2^

1. Blocco Operatorio, Azienda Ospedaliera Universitaria Luigi Vanvitelli, Napoli, Italy
2. Dipartimento di Sanità Pubblica, Università Federico II di Napoli, Napoli, Italy
3. Dipartimento di Oncologia, Ematologia e Terapie Cellulari, Ospedale Santobono Pausilipon, Napoli, Italy
4. Unità Operativa Complessa di Emodinamica, Azienda Ospedaliera di Rilievo Nazionale Antonio Cardarelli, Napoli, Italy
5. Dipartimento di Salute Mentale, Azienda Sanitaria Locale, Salerno, Italy
6. Area Dipartimentale dei Servizi Sanitari, Azienda Ospedaliera Universitaria Federico II, Napoli, Italy
7. Unità Operativa Complessa di Ortopedia e Traumatologia, Ospedale Santobono Pausilipon, Napoli, Italy
8. Dipartimento di Scienze Mediche Traslazionali, Università Federico II di Napoli, Napoli, Italy

* Correspondence: selenelalegname@gmail.com
**Abstract**
*Background/Objectives:* Chronic kidney disease-associated pruritus (CKD-aP) is a frequent and clinically significant symptom in patients with chronic kidney disease undergoing hemodialysis. It is associated with a substantial impact on quality of life and remains frequently underestimated in clinical practice. This study aimed to investigate the impact of uremic pruritus on the quality of life of patients undergoing hemodialysis. *Methods*: A single-center, cross-sectional observational study was conducted on 81 patients: adults with stage V chronic kidney disease, on dialysis for ≥3 months. Data were collected using a sociodemographic questionnaire, the Visual Analogue Scale, the SF-36 Health Survey, and the 5-D Pruritus Scale. Descriptive statistical analysis was performed using Jamovi software. *Results*: CKD-aP was identified in approximately 50% of the cohort, with 42.9% of participants reporting moderate-to-severe intensity. SF-36 scores showed marked impairment, particularly in the “General Health” and “Energy/Fatigue” domains. The 5-D Pruritus Scale indicated selective interference with sleep patterns and daily activities. Although male patients reported higher descriptive pruritus scores, the high heterogeneity and non-normal distribution of the data limit the generalizability of the findings. CKD-aP emerged as a determinant of reduced quality of life and a potential marker of clinical frailty. Even mild symptomatic presentations require clinical attention, as they were associated with decreased quality of life. *Conclusions*: The study confirms that uremic pruritus is a prevalent and clinically relevant symptom. A structured, multidimensional assessment is warranted, and its management should not be considered a secondary concern but rather an integral component of the care pathway for hemodialysis patients.
**Keywords:** chronic kidney disease-associated pruritus; uremic pruritus; hemodialysis; quality of life; chronic kidney disease

**SC03. Implementation of nurse-led ultrasound in vascular access monitoring: a strategy for the prevention of complications in hemodialysis**
Giuseppe Petruzzelli ^1,^*, Grazia Petruzzelli ^2^, Maria Luisa Biga ^3^, Pasquale Vincenzo Pietro ^4^, Giuseppe Quacquarelli ^4^, Ruggiero Lemma ^4^, Raffaella Pischetola ^4^ and Tiziana Piccolo ^5^

1. Dialysis Center, P.O. Barletta, ASL BT, Italy
2. NephroCare Dialysis Center, Minervino, Italy
3. U.O.C. Nephrology and Dialysis, ASL BAT, Italy
4. Dialysis Center, P.O. Barletta, ASL BT, Italy
5. U.O.C. Nephrology and Dialysis, ASL BAT, Italy

* Correspondence: giuseppe.terlizzi67@libero.it
**Abstract**
*Background/Objectives*: In the field of dialysis, preserving the native arteriovenous fistula (AVF) is a primary clinical goal. Nurse-led ultrasound has emerged as a fundamental tool for the morphological and functional assessment of vascular access, enabling timely and accurate monitoring. *Methods*: This case report describes A.P., a 76-year-old male with end-stage renal disease due to nephrolithiasis, on hemodialysis for 3 years, with a left distal radio-cephalic side-to-end AVF. During a routine nursing ultrasound at the Barletta dialysis center, an endoluminal hypoechoic area was detected 3 cm from the anastomosis. Assessment was completed by calculating the flow rate in the distal third of the brachial artery. Dialysis efficiency parameters remained stable, with normal Kt/V, dialysis clearance of 210 mL/min, and 6% recirculation. Following specialist consultation, a stenosis in the efferent vein was confirmed, and angioplasty was performed. A post-angioplasty nursing ultrasound the same day showed a marked reduction in the hypoechoic area compared to the pre-intervention scan. *Results*: This case report demonstrates the benefits of a monthly ultrasound prevention program, which allowed for the early identification of a complication, thereby preventing AVF failure. *Conclusions*: Nurse-led ultrasound represents an indispensable strategy for active and timely surveillance, enabling the detection of both current and potential future vascular access issues.
**Keywords:** nurse-led ultrasound; hemodialysis; arteriovenous fistula

**SC04. The electronic stethoscope: a new frontier for monitoring arteriovenous fistula (AVF) in dialysis**
Grazia Petruzzelli ^1,^*, Leonardo Di Leo ^2^, Giuseppe Petruzzelli ^3^ and Tiziana Piccolo ^4^

1. NephroCare Dialysis Center, Minervino, Italy
2. Pediatrics Unit, Bisceglie Hospital, Italy
3. Dialysis Center, Barletta Hospital, Italy
4. Nephrology Unit, Barletta Hospital, Italy

* Correspondence: grazia-petruzzelli@libero.it
**Abstract**
*Background/Objectives*: In hemodialysis patients, a well-functioning vascular access is essential for the effective delivery of therapy. Particularly, patients with AVF (arterovenous fistula) require regular observation of the arterial vessel. This continuous monitoring is crucial to detect early structural and functional abnormalities which, otherwise, could lead to reduced dialysis efficiency and access failure. This study aims to evaluate the usefulness of an electronic stethoscope in monitoring AVF in hemodialysis patients, through the early detection of vascular abnormalities such as stenosis or thrombosis. *Methods*: The study was conducted at the Dialysis Center of Monsignor Dimiccoli Hospital in Barletta and included 41 patients with native AVF. All patients underwent physical examination of the fistula, with particular focus on auscultation of the arterialized vessel using a Littmann Digital Core 8490 electronic stethoscope. Auscultation sounds were recorded and stored in a dedicated application. To assess the reliability of auscultation findings in relation to AVF function, selected cases showing abnormal sounds or non-continuous systolic-diastolic murmurs underwent confirmatory vascular ultrasound. All collected data were documented in a structured monitoring form. *Results*: A continuous systolic-diastolic bruit was observed in 80% of patients, while 15% presented a high-pitched (whistling) bruit and 5% a water-hammer sound. In cases with abnormal auscultation, ultrasound evaluation confirmed the presence of vascular abnormalities. *Conclusions*: Sound recording and graphical visualization allowed a more objective clinical assessment and proved useful for effective monitoring of AVF over time.
**Keywords:** arteriovenous fistula; electronic stethoscope; hemodialysis

**SC05. Abruzzo’s nephrodialysis nurses and palliative care**
Giovanna La Selva ^1,^* and Valeria Caponnetto ^2^

1. Complex Clinical Structure of Nefrology and dialysis, Hospital of Santo Spirito, Pescara, Italy
2. University of L’Aquila, Department of Life, Health and Environmental Sciences, L’Aquila, Italy

* Correspondence: g.laselva@gmail.com
**Abstract**
*Background/Objectives:* This study aims to investigate the knowledge, opinions, and self-perceived competence of nurses working in Nephrology and Dialysis units regarding palliative care, as well as their perceived need for further training in this field. *Methods*: A cross-sectional observational study was conducted by administering an anonymous questionnaire to a sample of approximately 130 nephrology and dialysis nurses from the Local Health Authorities (ASL) of Pescara, Chieti, and Teramo. The questionnaire consisted of four sections designed to collect data on the following areas: socio-demographic characteristics, nurses’ knowledge and opinions, the level of application of knowledge and available institutional tools for palliative care management, and training in the field of palliative care. *Results*: The study sample (N = 51; 72.5% female) showed a median age of 50 years and 14 years of clinical experience in nephrology. Only 13% of participants had completed post-graduate training. Findings highlighted significant knowledge gaps: 15% were familiar with palliative care guidelines, 25% with end-of-life (EoL) legislation (Law 219/2017), and 25% with patient assessment tools. Furthermore, 43% reported an absence of EoL management protocols. While participants strongly recognized the importance of multidisciplinarity (96%) and continuous reassessment of patient needs (82.4%), 51% experienced difficulties in approaching EoL care. *Conclusions*: To improve training standards according to national and international guidelines, a Professional Development Plan (PFA) on ‘Palliative Care in Nephrology’ has been launched for nephrology and dialysis nurses at ASL Pescara (currently awaiting authorization).
**Keywords:** nephrology; palliative care; professional training

**SC06. Listening to Chronicity: Narrative Medicine in the Nurse-Patient Relationship in Hemodialysis**
Francesca Zullino
U.O.S. Dialisi, Presidio Ospedaliero SS Annunziata, Chieti, Italy; francescamia@hotmail.com
**Abstract**
*Background/Objectives:* Chronic diseases represent a major challenge for contemporary healthcare systems due to their clinical, relational and existential impact. Chronic Kidney Disease (CKD) and hemodialysis treatment profoundly influence patients’ identity, daily life and social relationships. In this context, narrative medicine offers tools to better understand the subjective experience of illness and promote person-centered care. This study aims to explore the experiences of hemodialysis patients, with particular attention to the meaning attributed to chronicity and the nurse–patient relationship. *Methods*: An exploratory qualitative study based on a narrative nursing approach was conducted at the Hemodialysis Center of the “SS. Annunziata” Hospital in Chieti. Data were collected using a semi-structured instrument, self-administered and anonymous, consisting of 31 questions and a free narrative space. A convenience sample of 32 participants (18 men, 14 women; mean age 66.75 years; mean dialysis seniority 5.53 years) was recruited. Data were analyzed through inductive thematic analysis following Braun and Clarke’s framework. *Results*: Five main themes emerged: Diagnosis as a biographical disruption; The onset of hemodialysis as an invasive experience; Living with chronicity; The care relationship, in which empathy and listening are essential complements to technical skills; Future perspectives, fluctuating between hope and conscious acceptance. *Conclusions*: The findings highlight the importance of integrating narrative medicine into nursing practice. Technical skills must be complemented by relational competencies to effectively address patients’ emotional needs. Listening to patients’ stories enhances care quality, strengthens the therapeutic alliance, and supports adaptation to chronic illness.
**Keywords:** narrative medicine; chronic kidney disease; hemodialysis; nurse-patient relationship

**SC07. Artificial Intelligence in Ultrasound-Guided Placement of Central Venous Catheters for Hemodialysis and High-Flow Access: A Systematic Review of Clinical and Technological Evidence (AIDIAL-CVC)**
Ciro Pozzuoli ^1,^*, Anna Grimaldi ^2^, Giuseppe Frattolillo ^2^, Domenico Di Sivo ^3^, Incoronata Chiusolo ^4^ and Vincenzo Di Nuzzo ^5^

1. General Surgery Unit, Marcianise Hospital, Caserta Local Health Authority (ASL Caserta), Viale Sossietta Scialla, 81025, Marcianise, Caserta, Italy
2. Intensive Care Unit, Marcianise Hospital, Caserta Local Health Authority (ASL Caserta) Viale Sossietta Scialla, 81025, Marcianise, Caserta, Italy
3. Congenital Heart Disease Unit, Monaldi Hospital, Azienda Ospedaliera dei Colli, Via Leonardo Bianchi, 80131, Naples, Italy
4. University Hospital of Padua, Via Nicolò Giustiniani, 2, 35128, Padua, Italy
5. Emergency Department, Marcianise Hospital, Caserta Local Health Authority (ASL Caserta), Viale Sossietta Scialla, 81025, Marcianise, Caserta, Italy

* Correspondence: ciro.pozzuoli@students.uniroma2.eu
**Abstract**
*Background/Objectives*: Ultrasound-guided placement of central venous catheters (CVCs) for hemodialysis improves procedural success and reduces complications compared to landmark-based techniques. AI-integrated ultrasound enables automated vessel identification and real-time support, though evidence remains limited. This study evaluates clinical, nursing, and technological evidence on AI-assisted CVC placement. *Methods*: A systematic review was conducted and reported according to the PRISMA guidelines. The literature search was performed across MEDLINE, Embase, Scopus, CINAHL, and ClinicalTrials.gov databases and supplemented by grey literature sources, including primary studies. Data extraction followed the AIDIAL-CVC framework, specifically developed by the authors to integrate procedural outcomes (first-attempt success, number of punctures, procedural time, and mechanical complications) with AI performance metrics. Risk of bias was assessed using RoB 2, ROBINS-I, and QUADAS-AI tools. *Results*: AI applications include automated vascular segmentation, needle tracking, and real-time decision-support systems. AI-assisted ultrasound was associated with increased first-attempt success rates and reductions in the number of punctures and procedural time, with complication rates comparable to or lower than conventional techniques. Emerging solutions based on AI-guided portable robotic devices have demonstrated high accuracy in complex vascular access placement. *Conclusions*: In the nephrology setting, AI-integrated ultrasound systems represent a promising strategy to optimize CVC placement for hemodialysis. However, further high-quality studies and standardized reporting are needed, with particular attention to the integration of advanced nursing competencies and an interdisciplinary nursing–engineering approach.
**Keywords:** artificial intelligence; central venous catheter; hemodialysis

**SC08. Mild Hypothermia in DBD/DNC Donors (34–35 °C) and Kidney Transplant Outcomes: A Systematic Review of Randomized Studies from a Nursing Perspective**
Ciro Pozzuoli ^1^, Anna Grimaldi ^1,2,^*, Vincenzo Di Nuzzo ^1^, Federica Colella ^3^, Domenico Di Sivo ^4^, Incoronata Chiusolo ^5^ and Giuseppe Frattolillo ^1^

1. Marcianise Hospital, Caserta Local Health Authority (ASL Caserta), Caserta, Italy
2. Hospital Transplant Coordination (COP-P), Regional Transplant Network of Campania, Italy
3. Corporate Organ Procurement Coordinator, Regional Transplant Network of Campania, Italy
4. Monaldi Hospital, Azienda Ospedaliera dei Colli, Naples, Italy
5. University Hospital of Padua, Padua, Italy

* Correspondence: anna.grimaldi92a@gmail.com
**Abstract**
*Background/Objectives:* Delayed graft function (DGF) is an early complication of kidney transplantation linked to ischemia–reperfusion injury and donor management in intensive care, affecting graft outcomes. Targeted mild hypothermia (34–35 °C) has been proposed to reduce this risk, but evidence remains heterogeneous. This study evaluates whether its use in brain-dead donors before organ procurement reduces DGF in adult recipients, from a nursing perspective. *Methods:* A systematic review was conducted and reported according to PRISMA guidelines. Literature searches were performed in MEDLINE, Embase, Scopus, CINAHL, and ClinicalTrials.gov, and supplemented by grey literature. Randomized controlled trials including donors declared dead by neurological criteria and undergoing mild hypothermia prior to kidney procurement were selected. The primary outcome was delayed graft function, defined as the need for dialysis within the first 7 days after transplantation. Risk of bias was assessed using the Cochrane Risk of Bias 2 tool, and the certainty of evidence was evaluated using the GRADE approach. *Results*: Mild donor hypothermia reduced DGF incidence compared to normothermia, with greater benefit in high-risk donors. Pooled data suggest improved early renal function, though with notable heterogeneity. Recent evidence indicates a smaller effect in low-risk donors, suggesting interaction with donor characteristics. *Conclusions*: Mild hypothermia in DBD/DNC donors is a clinically relevant intervention for advanced nursing practice. Temperature monitoring and specialized nursing skills may improve adherence to targets and reduce care variability, supporting interdisciplinary clinical innovation.
**Keywords:** systematic review; hypothermia; kidney transplantation

**SC09. Nursing masterclass and quality of care in renal replacement therapy: evaluation of effectiveness at one-year follow-up**
Carolina Circognini ^1,^*, Angelo Pirreco ^1,4^, Salvatore Bonaventura ^1^, Giusi Pecora ^1^, Carmelita Marcantoni ^1^, Angelo Gambera ^2^ and Vincenzo Parrinello ^3^

1. U.O. “Nefrologia e Dialisi”, AOUP “G. Rodolico—San Marco”, 95123 Catania, Italy;
2. Dipartimento di Scienze Mediche, Chirurgiche e Tecnologie Avanzate “G.F. Ingrassia” (DGFI). Università degli Studi di Catania, 95123 Catania, Italy;
3. U.O. “Qualità e Rischio Clinico”, AOUP “G. Rodolico—San Marco”, 95123 Catania, Italy;
4. Dipartimento di Biomedicina e Prevenzione, Università degli Studi di Roma “Tor Vergata”, Roma, Italy

* Correspondence: carolinacircognini1@gmail.com
**Abstract**
*Background/Objectives:* Renal replacement therapy (HD: Haemodialysis, PD: Peritoneal dialysis, CRRT: Continuous renal replacement therapy) requires advanced nursing competencies across clinical, technical and organizational domains. In highly complex care settings, structured education is essential to ensure quality, safety and standardization of clinical practice. This study aimed to assess the effectiveness of a nursing masterclass in strengthening competencies and its perceived impact on quality of care at one-year follow-up. *Methods*: A single-center observational study with a quantitative–qualitative approach was conducted. The masterclass (October-November 2024) involved 18 nurses from nephrology and critical care areas focusing on vascular access, HD, PD, and CRRT. The program combined theoretical education and practical training with multidisciplinary teaching. At one-year follow-up, an anonymous questionnaire including a Likert scale (1–5) and open-ended questions was administered to assess satisfaction, competency retention and perceived impact on care quality and work organization. Quantitative data were analyzed using descriptive statistics while open-ended responses were examined through thematic categorization. *Results:* The questionnaire was completed by 12 participants (66.7%) reflecting variability in clinical setting and professional experience. Satisfaction averaged 4.5/5, while 75% of respondents assigned a score ≥4 to the item concerning improvement in quality of care. Practical application of acquired competencies was relevant in the CRRT modules. Participants also reported increased confidence in managing dialysis treatments. *Conclusions:* The masterclass was effective in strengthening nursing competencies and improving perceived quality of care. This educational model may represent a sustainable and transferable tool for clinical governance and patient safety.
**Keywords:** nursing training; renal replacement therapy; quality of healthcare

**SC10. The role of the wound care specialist nurse in nephrology settings in the management of chronic kidney disease–associated pruritus: a phenomenological study**
Giovanni Gavarro ^1,^*, Domenico Di Sivo ^2^, Incoronata Chiusolo ^3^, Giuseppe Frattolillo ^1^, Ciro Pozzuoli ^4^, Vincenzo Di Nuzzo ^5^, Gaetano Ferrara ^6^ and Anna Grimaldi ^1^

1. Intensive Care Unit, Marcianise Hospital, Caserta Local Health Authority (ASL Caserta) Viale Sossietta Scialla, 81025 Marcianise, Caserta, Italy
2. Congenital Heart Disease Unit, Monaldi Hospital, Azienda Ospedaliera dei Colli, Via Leonardo Bianchi, 80131 Naples, Italy;
3. University Hospital of Padua, Via Nicolò Giustiniani, 2, 35128 Padua, Italy;
4. General Surgery Unit, Marcianise Hospital, Caserta Local Health Authority (ASL Caserta), Viale Sossietta Scialla, 81025 Marcianise, Caserta, Italy;
5. Emergency Department, Marcianise Hospital, Caserta Local Health Authority (ASL Caserta), Viale Sossietta Scialla, 81025 Marcianise, Caserta, Italy;
6. Nephrology and Dialysis Unit, Ramazzini Hospital, Via Guido Molinari, 2, 41012, Carpi, Modena, Italy

* Correspondence: gavarro.giovanni96@gmail.com
**Abstract**
*Background/Objectives:* Chronic kidney disease–associated pruritus (CKD-aP) is a frequent and disabling symptom among dialysis patients, yet it often remains marginal in care processes. The role of the wound care specialist nurse as a consultative resource in nephrology has been minimally explored. This study aims to understand the lived experience of wound care specialist nurses working as consultants in the management of CKD-aP in hospital nephrology settings. *Methods*: A qualitative study was conducted using a descriptive phenomenological approach grounded in Husserlian philosophy and analyzed according to Giorgi’s method. Fifteen wound care specialist nurses working as consultants in nephrology and dialysis services were recruited through purposive sampling. Individual semi-structured interviews were audio-recorded, transcribed verbatim, and analyzed through holistic reading, identification of meaning units, phenomenological transformation, and synthesis of the essential structure, supported by strategies to ensure methodological rigor (bracketing, audit trail, and researcher triangulation). *Results*: Five meaning structures emerged: (1) invisibility yet centrality of pruritus in patients’ experience; (2) mediation and clinical translation of lived experience, with the need to legitimize the symptom; (3) negotiated professional identity, with variable team inclusion; (4) organizational constraints (limited time, fragmented communication, delayed access) affecting effectiveness; (5) emotional and transformative impact of the role. *Conclusions*: The consultative role of the wound care specialist nurse emerges as a relational, interpretive, and organizational practice, in addition to a technical one. Its recognition could enhance the visibility of complex symptoms and improve care integration within nephrology services.
**Keywords:** CKD-aP; wound care specialist nurse; phenomenological study

**SC11. Waste management in hemodialysis: measurement, challenges, and improvement interventions. The key role of nurses in the environmental sustainability of the dialysis center.**
Rita Pecoraro ^1,^*, Giuseppe Di Chiaro ^2^, Gaetano Alfano ^1^, Patrizia Scannavini ^4^, Patrizia Albinelli ^4^, Stefania Sarti ^5^, Anna Rita Villani ^1^ and Gabriele Donati ^1,3^

1. Nephrology, Dialysis and Kidney Transplant Unit, University Hospital of Modena, Modena, Italy
2. PhD Program in Clinical and Experimental Medicine (CEM), Department of Biomedical, Metabolic and Neurosciences, University of Modena and Reggio Emilia, Modena, Italy
3. CHIMOMO Department, University of Modena and Reggio Emilia, Modena, Italy
4. Hospital Hygiene Unit, University Hospital of Modena, Modena, Italy
5. Hospital Hospitality Services, University Hospital of Modena, Modena, Italy

* Correspondence: rita.pecoraro96@gmail.com
**Abstract**
*Background/Objectives*: Waste management is a key component of sustainability in dialysis centers, as hemodialysis generates significant amounts of special waste associated with considerable CO_2_ emissions^1^. This study, conducted within the European KitNewCare project, aims to analyze waste management processes in hemodialysis, identifying critical issues and potential improvement strategies to reduce both waste volume and environmental impact. *Methods*: Nursing practices during a single dialysis session were observed, and devices and packaging were weighed at the beginning and end of treatment, with data stratified by treatment modality and machine type. The analysis was complemented by an anonymous questionnaire administered to nursing staff and a multidisciplinary audit involving physicians, nurses, and hospital hygiene services. A new agreement on waste management was also included to improve paper and plastic differentiation. *Results*: Results show a waste production of 1.7–1.8 kg per session in standard hemodialysis and 1.6–2.0 kg in online hemodiafiltration, with variations depending on the equipment used. Key issues include technical limitations of some machines and difficulties in proper waste classification. In response, several improvement actions were implemented, including upgrading dialysis machines, adopting environmentally sustainable practices, developing operational tools to support waste segregation, and introducing monthly waste monitoring. *Conclusions*: The study demonstrates that objective measurement and audit processes can transform technical and procedural challenges into concrete organizational improvements, enhancing both quality of care and environmental sustainability. Future steps will focus on monitoring waste production and estimating the impact on CO_2_ emissions after implementation.
**Keywords:** hemodialysis; healthcare waste; environmental sustainability

**SC12. Community-Based Care and Pre-Dialysis CKD: A Proposal for the Implementation of the Community Nurse for Early Identification and Community-Based Management**
Angelo Pirreco ^1,9,^*, Gaetano Gallo ^2,9^, Milena Gigliello ^3,9^, Erika Acquistapace ^4,9^, Viviana Carù ^5,9^, Raffaele Consoli ^6,9^, Francesco Sacchini ^7,9^, Giovanni Cangelosi ^8,9^, Dhurata Ivziku ^10^ and Daniela Tartaglini ^10,11^

1 Department of Biomedicine and Prevention, University of Rome “Tor Vergata”, Rome, Italy
2 Nephrology and Dialysis Unit, Local Health Authority of Piacenza, Piacenza, Italy
3 Department of Surgery, “Santa Maria della Speranza” Hospital, Local Health Authority of Salerno, Salerno, Italy
4 Department of Emergency Medicine, Emergency Department, Valduce Hospital, Como, Italy
5 Department of Cardiology, Circolo and Fondazione Macchi Hospital, ASST Sette Laghi, Varese, Italy
6 Department of Nephrology, Dialysis and Renal Transplantation, Fondazione IRCCS Ca’ Granda Ospedale Maggiore Policlinico, Milan, Italy
7 Community Hospital of Sant’Elpidio a Mare and Fermo Correctional Facility, Local Health Authority AST Fermo, Fermo, Italy
8 School of Pharmacy, Experimental Medicine and “Stefania Scuri” School of Public Health, University of Camerino, Camerino, Macerata, Italy
9 Italian Society of Nephrology Nurses (SIAN), Via Capotesta 1/30, 07026 Olbia, Italy, Public Health Research Group, Italy
10 Directorate of Healthcare Professions, Campus Bio-Medico University Hospital Foundation, Rome, Italy
11 Nursing Research Unit, Campus Bio-Medico University of Rome, Rome, Italy

* Correspondence: angelo.pirreco@students.uniroma2.eu
**Abstract**
*Background/Objectives:* Chronic Kidney Disease (CKD) is a major public health priority due to its high prevalence, silent progression, and prognostic burden. The Italian Ministerial Decree 77/2022 promotes the reorganization of community-based care, strengthening territorial services such as Community Houses. The 2024 KDIGO guidelines emphasize early risk stratification, while the 2025 national Preventive Diagnostic Therapeutic Care Pathway (PPDTA) framework supports integrated care, secondary prevention, and reduction of late referral. This study proposes an implementation model of the Community Nurse to improve early identification and management of pre-dialysis CKD in community-based settings. *Methods:* A qualitative literature review on patient and professional experiences was conducted alongside an analysis of national healthcare policies. Based on these findings, a change management proposal was developed to support the integration of the Community Nurse into Community Houses and General Practice settings. *Results:* The model identifies the Community Nurse as a key professional for early detection, therapeutic education, patient empowerment, and multiprofessional coordination. Core activities include community-based screening of risk factors (diabetes, hypertension, family history), application of KDIGO criteria, structured nursing counselling, early activation of nephrology referral pathways, and monitoring through process and outcome indicators aligned with the national PPDTA. This approach enhances continuity of care and reduces late referral. *Conclusions*: Integrating the Community Nurse into community-based care is a feasible strategy to improve early CKD detection, slow disease progression, and optimize referral pathways. This model may reduce complications and healthcare costs associated with advanced CKD, improving sustainability and equity in healthcare systems.
**Keywords:** chronic kidney disease; community-based care; community nurse

**SC13. Nursing Students’ Burden in Hemodialysis Settings: Exploring Lived Experiences and Risk of Burnout**
Sofia Zagame ^1^ and Angelo Pirreco ^2,^*

1 Clinica Gretter—Istituto Clinico Vidimura srl, Piazza Mancini Battaglia, 5, 95126 Catania, Italy
2 Department of Biomedicine and Prevention, University of Rome “Tor Vergata”, Rome, Italy

* Correspondence: angelo.pirreco@students.uniroma2.eu
**Abstract**
*Background/Objectives:* Hemodialysis units are complex clinical environments characterized by high technological intensity, chronic patient care, and emotionally demanding interactions. Nursing students undertaking clinical placements in these settings may experience significant psychological and educational burden, potentially increasing the risk of burnout, recognized as an occupational phenomenon associated with chronic workplace stress. This study aims to explore the lived experiences of nursing students in hemodialysis settings and to identify factors associated with perceived burden and early signs of burnout. *Methods*: A qualitative study was conducted using a phenomenological approach. Nursing students who completed clinical placements in hemodialysis units were recruited through purposive sampling. Data were collected through semi-structured interviews focusing on emotional experiences, learning processes, relational dynamics, and perceived stressors. Interviews were audio-recorded, transcribed verbatim, and analyzed using thematic analysis according to Braun and Clarke. *Results*: Students perceived hemodialysis settings as highly technical and emotionally demanding. Key themes included (1) gap between theoretical preparation and clinical complexity, (2) central role of clinical tutors in learning and emotional support, (3) emotional burden characterized by anxiety, fatigue, and fear of errors, and (4) heterogeneous coping strategies, including peer support and adaptive engagement. Despite these challenges, students maintained relational involvement with patients without clear signs of depersonalization. *Conclusions*: Clinical placements in hemodialysis units expose nursing students to significant emotional and cognitive demands, contributing to early indicators of burnout. Structured mentorship, targeted educational preparation, and psycho-educational support are recommended to improve learning experiences and prevent burnout risk.
**Keywords:** nursing students; hemodialysis; burnout; clinical education; qualitative research; student experience

**SC14. Impact of an AI-Based Assistant on the Quality of Individualized Care Plans in Patients Undergoing Renal Replacement Therapy: A Randomized Crossover Study**
Anna Grimaldi ^1,^*, Ciro Pozzuoli ^2^, Giuseppe Frattolillo ^1^, Domenico Di Sivo ^3^, Incoronata Chiusolo ^4^ and Vincenzo Di Nuzzo ^5^

1 Intensive Care Unit, Marcianise Hospital, Caserta Local Health Authority (ASL Caserta) Viale Sossietta Scialla, 81025 Marcianise, Caserta, Italy
2 General Surgery Unit, Marcianise Hospital, Caserta Local Health Authority (ASL Caserta), Viale Sossietta Scialla, 81025 Marcianise, Caserta, Italy
3 Congenital Heart Disease Unit, Monaldi Hospital, Azienda Ospedaliera dei Colli, Via Leonardo Bianchi, 80131 Naples, Italy
4 University Hospital of Padua, Via Nicolò Giustiniani, 2, 35128, Padua, Italy;
5 Emergency Department, Marcianise Hospital, Caserta Local Health Authority (ASL Caserta), Viale Sossietta Scialla, 81025 Marcianise, Caserta, Italy

* Correspondence: anna.grimaldi92a@gmail.com
**Abstract**
*Background/Objectives*: The Individualized Care Plan (ICP) is central to nursing in nephrology, ensuring continuity, safety, and patient-centered care in renal replacement therapy. The integration of diagnoses, outcomes, and interventions using standardized NANDA-I, NOC, and NIC (NNN) languages is a key indicator of care quality. However, clinical complexity and documentation burden may affect completeness. Generative artificial intelligence based on Large Language Models (LLMs) is emerging to support healthcare documentation, although evidence in nephrology remains limited. This study evaluates the impact of a generative AI-based assistant on the quality and efficiency of ICPs. *Methods*: A randomized crossover experimental study was conducted using simulated clinical cases. Twenty nephrology nurses developed ICPs based on four clinical vignettes under two conditions: standard documentation and AI-assisted support using a Large Language Model (draft reviewed by the nurse). Quality was assessed using an ad hoc checklist by two independent blinded reviewers; completion times were recorded. *Results*: A total of 160 ICPs were analyzed (80 standard, 80 AI-assisted). AI-assisted ICPs achieved higher mean quality scores than standard documentation. The proportion of high-quality ICPs increased, while completion time decreased. High inter-rater reliability was observed. *Conclusions*: Generative AI, under nursing supervision, improves the quality and efficiency of care planning in nephrology. Further studies in real clinical settings are needed to assess safety and applicability.
**Keywords:** individualized care plans; artificial intelligence; nursing care

**SC15. BVM-C: The Link between Patient, Doctor, and Nurse**
Enrico Lo Voi ^1^, Lucia Michele Clavier ^2^ and Barbara Padiglioni ^3,^*

1 S.C. Nefrologia e Dialisi, Ospedale di Assisi, USL Umbria 1, Assisi, Perugia, Italy
2 S.C. Nefrologia e Dialisi, Ospedale di Castiglione del Lago, USL Umbria 1, Castiglione del Lago, Perugia, Italy
3 S.C. Nefrologia e Dialisi, Ospedale della Media Valle del Tevere, USL Umbria 1, Todi, Perugia, Italy

* Correspondence: barbara.padiglioni@uslumbria1.it
**Abstract**
*Background/Objectives*: Tolerance of hemodialysis treatments is as important as solute clearance efficiency. Automatic modulation of hourly ultrafiltration and sodium concentration helps prevent abrupt changes in blood volume. This study aimed to implement the Surdial X BVM-C module in routine clinical practice as a daily tool to improve patient well-being and achieve better control of hydration status. *Methods*: By applying BVM in open-loop mode for two weeks, the critical blood volume (Vercrit) for each patient was identified, i.e., the maximum tolerable threshold of relative reduction. Subsequently, the relationship between Vercrit and each patient’s total ultrafiltration (UF) was quantified, reflecting refilling capacity per kg of UF. A dynamic Vercrit was then established as a function of the prescribed UF: this is the key BVM-C parameter that modulates UF/h and sodium profiles to achieve a gradual, asymptomatic reduction in blood volume. *Results*: Use of BVM-C improved treatment delivery in unstable patients, in parallel with a significant increase in quality of life (QoL). Case reports: Female, 70 years old, with type 2 diabetes mellitus, micro- and macroangiopathic complications, and marked intradialytic cardiovascular instability, frequently requiring interruption of UF or discontinuation of the session. BVM-C enabled gradual intradialytic hemodynamic stabilization and a reduction in dry weight. In parallel, a clear decrease in NT-proBNP was observed. Male, 86 years old, with low blood pressure values both intra- and interdialytically. He showed no evidence of sodium and water retention; however, the intradialytic blood volume curve and NT-proBNP values suggested the need to reduce dry weight. BVM-C made it possible to reduce dry weight without intradialytic complications, with a concomitant reduction in NT-proBNP, suggesting improved cardiac compensation. *Conclusions*: BVM-C proved to be a simple and effective tool to improve treatment tolerance, optimize dry weight, and harmonize collaboration between physicians who prescribe and nurses who deliver personalized dialysis therapies.
**Keywords:** collaboration; blood volume; refilling

**SC16. Effectiveness of Antimicrobial Uniforms in a Dialysis Unit: Design of a Pilot Study on Infection Reduction and Healthcare Worker Safety**
Emanuele Orlandi ^1,^*, Valerio Di Nardo ^2^ and Luigi Vecchi ^3^

1 Dialysis and Nephrology Unit, S. Maria Hospital, Terni, Italy
2 Staff Education and Training, S. Maria Hospital, Terni, Italy
3 Dialysis and Nephrology Unit, S. Maria Hospital, Terni, Italy

* Correspondence: e.orlandi@aospterni.it
**Abstract**
*Background/Objectives:* Healthcare-associated infections (HAIs) remain a major concern in dialysis units, where patient frailty and the frequent management of vascular access significantly increase the risk of microbial transmission. Repeated manipulation of central venous catheters (CVCs) further heightens susceptibility to infection. Laboratory studies have shown that technical fabrics treated with zinc oxide nanoparticles (ZnO NPs) possess strong antibacterial and antiviral properties. Their effectiveness has been confirmed in hospital environments through a reduction in nosocomial infection rates. However, evidence supporting the use of antimicrobial uniforms in dialysis-specific clinical settings remains limited. Primary objective: to reduce microbial contamination on clinical surfaces and healthcare worker uniforms during routine activities and to assess a potential decrease in HAIs, particularly CVC-related infections. Secondary: evaluating healthcare workers’ perceived safety, protection, and comfort, as well as assessing the cost–benefit ratio of antimicrobial uniform use. *Methods*: This 12-month prospective pilot study includes institutional approval, routine use uniforms with zinc oxide nanoparticles (ZnO NPs) by dialysis staff, and biweekly microbiological sampling of clinical surfaces, workstations, and uniforms. Infection-related data will be collected before and after uniform implementation (T0–T1). Structured questionnaires will assess perceived safety, and HAI rates will be analyzed throughout the study. *Expected Results:* A reduction in environmental microbial load and HAI incidence is anticipated, along with improved perceived safety among healthcare workers. *Conclusions*: The adoption of antimicrobial uniforms may represent an innovative strategy for HAI prevention in dialysis units. This pilot study aims to provide preliminary evidence to support future larger, multicenter investigations on ZnO NP-treated garments.
**Keywords:** antimicrobial textiles; dialysis; hai prevention

**SC17. Anticoagulation in CRRT: Evidence from an Integrative Review and Clinical-Nursing Implications**
Gaetano Artiola, Vincenzo Pettillo, Kevin Generoso, Serena Caparroti, Fabiana Sommella and Roberto Colonna
Post-operative intensive care, critical area department, AO dei Colli, Napoli, Italy; gart1587@gmail.com
**Abstract**
*Background/Objectives:* Continuous Renal Replacement Therapy (CRRT) is the preferred treatment for critically ill patients with acute kidney injury and hemodynamic instability. Anticoagulation of the extracorporeal circuit is essential to maintain circuit patency and ensure treatment continuity. The main strategies include unfractionated heparin (UFH), low-molecular-weight heparin (LMWH), and regional citrate anticoagulation (RCA). Current KDIGO guidelines recommend citrate as the first-line option in the absence of contraindications, although significant variability persists in clinical practice. This study aimed to evaluate the efficacy and safety of different anticoagulation strategies in CRRT and to explore their clinical and nursing implications. *Methods:* An integrative literature review was conducted using PubMed, Embase, and Cochrane databases up to 2026. Randomized controlled trials, observational studies, systematic reviews, and international guidelines addressing anticoagulation in CRRT were included. The primary outcome was filter lifespan, while secondary outcomes included bleeding events, circuit clotting, metabolic complications, and mortality. A total of 10 studies were included, comprising 7 clinical studies or comparative analyses and 3 guidelines or consensus documents. *Results*: Regional citrate anticoagulation significantly increased filter lifespan compared with heparin (approximately 36.7 vs. 16.4 h) and reduced major bleeding events (2.4% vs. 12.6%). Circuit clotting rates were also lower with citrate (21.3% vs. 50.7%). No significant differences were observed in hospital or 28–90-day mortality. However, citrate use was associated with a higher incidence of metabolic complications, particularly hypocalcemia and metabolic alkalosis, requiring close laboratory monitoring. *Conclusions*: RCA represents the preferred anticoagulation strategy in CRRT due to improved circuit lifespan and reduced bleeding risk. Standardized protocols and advanced nursing competencies are essential to optimize safety and treatment effectiveness in critically ill patients.
**Keywords:** CRRT; anticoagulation; citrate; heparin; intensive care

**SC18. Early Recognition of Clinical Deterioration in the Nephropathic Patient: Role of the Nephrology Nurse and Integration with Early Warning Systems**
Carmela Fatone ^1^, Martina Cuomo ^2^, Ciro Bellotta ^3^, Anna Musica ^4^ and Anna Grimaldi ^4^

1 Emergency Gastroenterology and Digestive Endoscopy AORN “Antonio Cardarelli”, 9 Cardarelli Street, 80131 Naples, Italy
2 Pain Management Unit, Monaldi Hospital, Azienda Ospedaliera dei Colli, Via Leonardo Bianchi, 80131 Naples, Italy
3 Intensive Care Unit AORN “Antonio Cardarelli”, 9 Cardarelli Street, 80131 Naples, Italy
4 Intensive Care Unit, Marcianise Hospital, Caserta Local Health Authority (ASL Caserta) Viale Sossietta Scialla, 81025 Marcianise, Caserta, Italy

* Correspondence: anna.grimaldi92a@gmail.com
**Abstract**
*Background/Objectives:* The nephropathic patient presents high care complexity and is exposed to a significant risk of acute clinical deterioration, often preceded by early, non-specific signs that are difficult to recognize. Timely identification of these signals is essential to prevent complications and improve clinical outcomes. In this context, the nephrology nurse plays a central role in continuous monitoring and early detection of warning signs. This study aims to analyze the available evidence on early recognition of clinical deterioration in nephropathic patients, with particular attention to the role of the nephrology nurse, warning indicators, and integration with early warning systems. *Methods:* A narrative literature review was conducted following established methodological approaches, through consultation of PubMed and CINAHL databases. Recent studies and reviews related to clinical monitoring of patients with acute and chronic kidney disease and the use of early deterioration detection systems were included. *Results*: The evidence identifies the main early indicators as: reduced urine output, alterations in fluid balance and volume status, electrolyte imbalances (particularly hyperkalemia), changes in level of consciousness, and hemodynamic instability. The integration of systematic nursing observation with early warning systems enables more timely recognition of clinical deterioration and promotes early activation of appropriate clinical and care interventions. *Conclusions*: The nephrology nurse plays a key role in the early identification of clinical deterioration. The adoption of structured monitoring models and integration with early warning tools improve patient safety and clinical outcomes. Promoting advanced training and shared care protocols in nephrology settings is essential.
**Keywords:** nephropathic patient; clinical deterioration; nephrology nursing

**SC19. Vascular Remote Care: The New Digital Frontier in Monitoring the Management of AVF Access**
Martina Cuomo ^1^, Carmela Fatone ^2^, Ciro Bellotta ^3^, Anna Musica ^4^ and Anna Grimaldi ^4^

1 Pain Management Unit and TAV, Monaldi Hospital, Azienda Ospedaliera dei Colli, Via Leonardo Bianchi, 80131 Naples, Italy
2 Emergency Gastroenterology and Digestive Endoscopy AORN “Antonio Cardarelli”, 9 Cardarelli Street, 80131 Naples, Italy
3 Intensive Care Unit AORN “Antonio Cardarelli”, 9 Cardarelli Street, 80131 Naples, Italy
4 Intensive Care Unit, Marcianise Hospital, Caserta Local Health Authority (ASL Caserta) Viale Sossietta Scialla, 81025 Marcianise, Caserta, Italy

* Correspondence: cuomo.martina92@gmail.com
**Abstract**
*Background/Objectives:* The arteriovenous fistula (AVF) is the gold standard for vascular access in hemodialysis patients but remains vulnerable to preventable complications such as stenosis and thrombosis, the main causes of access loss. International guidelines recommend continuous and structured surveillance; however, traditional intermittent assessments often delay dysfunction detection. Telenursing has proven effective in chronic care, improving self-care and continuity, while remote monitoring systems are feasible and effective in dialysis settings. This study aims to describe an innovative Vascular Remote Care nursing model for home AVF monitoring. *Methods*: A clinical-organizational protocol was developed based on evidence-based principles, centered on the nephrology nurse. It integrates advanced nursing surveillance, patient education for self-monitoring, digital platforms for clinical and symptom data collection, and nurse-managed early warning systems. The model includes remote follow-up and hospital–community integration, with future implementation of predictive algorithms for early detection of AVF dysfunction risk. *Expected Results*: The adoption of this model is expected to improve early identification of complications, reduce vascular access loss, and decrease unplanned hospital admissions. It is also hypothesized to increase patient engagement and adherence to monitoring, with a potential positive impact on healthcare system sustainability and optimization of care pathways. *Conclusions*: Vascular Remote Care based on telenursing represents an evolution of the nephrology care model, integrating advanced nursing skills and digital technologies in a proactive, patient-centered approach. This paradigm strengthens the nurse’s role in vascular access management and represents a concrete opportunity to improve clinical outcomes, paving the way for future effectiveness studies.
**Keywords:** arteriovenous fistula; telenursing; nephrology nursing

**SC20. Experiences of Patients and Healthcare Professionals in Pre-Dialysis Chronic Kidney Disease: A Qualitative Systematic Review**
Erika Acquistapace ^1^, Giovanni Cangelosi ^2^, Viviana Carù ^3^, Raffaele Consoli ^4^, Gaetano Gallo ^5,^*, Dhurata Ivziku ^6^, Milena Gigliello ^7^, Francesco Sacchini ^8^, Daniela Tartaglini ^9^ and Angelo Pirreco ^10^

1 Department of Emergency Medicine, Emergency Department, General Hospital di Zona Valduce, Como, Italy
2 School of Pharmacy, Experimental Medicine and “Stefania Scuri” Public Health Department, University of Camerino, 62032 Camerino, Macerata, Italy
3 Department of Cardiology, Circolo e Fondazione Macchi Hospital, ASST Sette Laghi, Varese, Italy
4 Department of Nephrology, Dialysis and Renal Transplantation, Fondazione IRCCS Ca’ Granda Ospedale Policlinico, Milan, Italy
5 U.O.C. Nefrologia e Dialisi, Azienda Unità Sanitaria Locale of Piacenza, Piacenza, Italy
6 Direzione Assistenziale delle Professioni Sanitarie, Fondazione Policlinico Universitario Campus Biomedico, Rome, Italy
7 Department of Surgery, “Santa Maria della Speranza” Hospital, Local Health Authority of Salerno, Salerno, Italy
8 Community Hospital of Sant’Elpidio a Mare and Fermo Correctional Facility, Local Health Authority AST Fermo, Fermo, Italy
9 Facoltà di Medicina e Chirurgia, Università Campus Biomedico di Roma, Roma, Italy
10 Department of Biomedicine and Prevention, University of Rome “Tor Vergata”, Rome, Italy

* Correspondence: g.gallo@ausl.pc.it
**Abstract**
*Background/Objectives:* Chronic Kidney Disease (CKD) in the pre-dialysis phase represents a crucial window to slow disease progression and reduce the need for renal replacement therapies. However, the lived experience of patients in stages 3–4 and that of healthcare professionals involved in their care remain insufficiently explored in a systematic way. This study aimed to synthesize qualitative evidence on the experiences of patients with pre-dialysis CKD and healthcare professionals, identifying key domains influencing engagement, self-management, and continuity of care. *Methods*: Two qualitative systematic reviews with meta-synthesis were conducted according to the Preferred Reporting Items for Systematic Reviews and Meta-Analyses (PRISMA) guidelines. Literature searches were performed in PubMed, Scopus, CINAHL, MEDLINE, Embase, Ovid, Web of Science, and the Cochrane Library (2000–2025). Studies involving adult patients with CKD stages 3a–4 and healthcare professionals were included. Methodological quality was assessed using the Joanna Briggs Institute checklist. Data were analyzed through inductive thematic synthesis. *Results*: Eighteen studies were included for patients and twenty-one for professionals. Six main themes emerged for patients: illness perception, symptoms, impact on daily life, knowledge and self-management, coping strategies, and relationships with healthcare services. Patients reported limited knowledge and difficulties in self-management. From the professionals’ perspective, key themes included communication, care organization, decision support, professional role, and interprofessional collaboration. *Conclusions*: Pre-dialysis CKD is an under-recognized condition. Interventions targeting communication, education, and integrated care pathways are essential to improve patient engagement and care continuity.
**Keywords:** chronic kidney disease; pre-dialysis; experiences

**SC21. Health and illness, or illness and health? What if we had read the map backwards?**
Marisa Pegoraro
Palliative Care Study Group, Italian Society of Nephrology Nurses (SIAN), Via Capotesta 1/30, 07026 Olbia, Italy; marisapegoraro.996@gmail.com
**Abstract**
*Background/Objective:* Despite increasing healthcare spending, there is no proportional improvement in the population’s health status, while illness appears to be increasingly widespread. This raises questions about the true understanding of biological processes and the very nature of illness. Biology, understood as the “logic of life,” uses criteria and modes of relationship that are not fully integrated into current medicine, which often lacks a holistic and evolutionary vision of illness. In this context, nursing can represent an innovative operational space. The objective is to present Ryke Geerd Hamer’s conceptual map and analyze the so-called “5 biological laws,” which describe the connections between the mind, brain, and organs, and the biological meaning of illness. *Methods*: A narrative review of the literature was conducted, with examples of how the same event, experienced with different emotional meanings, can generate different physiological responses. These responses are interpreted in relation to criteria and evolutionary stages of phylogeny, ontogeny, and embryogenesis, as well as the role of the autonomic nervous system in regulating the activation and recovery phases. *Results*: The analysis proposes a reading of illness as the expression of an adaptive biological program activated in response to emotionally significant events. The body is described as a system oriented toward survival and repair, regulated by the alternation between activity and rest mediated by the sympathetic and parasympathetic nervous systems. *Conclusions*: Knowledge of the so-called “New Medicine” suggests an integrative perspective on illness and the meaning of symptoms, potentially useful for expanding the role of nursing in health promotion. These approaches deserve further study and critical evaluation in scientific and professional contexts.
**Keywords:** new medicine; biology; embryogenesis

**SC22. The Physical Symptom: A Gateway to the True Origins of Illness. Dedicated to Nurses**
Marisa Pegoraro
Palliative Care Study Group, Italian Society of Nephrology Nurses (SIAN), Via Capotesta 1/30, 07026 Olbia, Italy; marisapegoraro.996@gmail.com
**Abstract**
*Background/Objective:* Nursing is based on a holistic view of the person, considered as a bio-psycho-socio-relational unity. Despite over thirty years of academic development, this approach struggles to translate into organizational and clinical models, fully integrated into contemporary medicine. In practice, the experience of illness is often accompanied by a perceived incongruence between clinical definition and subjective experience. Some paradigms of quantum physics emphasize the inter-connection of phenomena and the role of the observer in the observed processes suggesting the need to broaden the interpretative frameworks, even in the healthcare setting. From this perspective, the symptom can be considered not only as a pathological expression, but as a meaningful signal within the context of the individual’s history. The objective is to present tools for self-awareness and interpretation of the subjective symptom, placed within the biological and psycho-socio-relational context of the individual. *Methods*: A narrative review of the conceptual foundations of the “ENIA” and “Ascolto Biologico^®^” methods was conducted. Application examples and experiential proposals based on Epoché (suspension of judgment), Narration, Intuition, and Action were analyzed. Particular attention was paid to the difference between subjective description of the symptom and standard diagnostic classification, as well as the role of language in constructing meaning. *Results*: The analysis suggests that integrating subjective narrative and biological interpretation of the symptom can foster greater awareness. This approach highlights the organism’s potential for self-regulation and contributes to a less threatening reworking of the illness experience. *Conclusions*: The proposed approach offers an integrative perspective useful in both personal and professional settings, consistent with the principles of person-centered care. Further research is needed to evaluate its applicability and integration in clinical and educational contexts.
**Keywords:** holism; symptom; narrative

**SC23. The implementation of Primary Nursing in the continuous clinical monitoring of patients who are candidates for renal transplantation**
Bianca Asbusti ^1,^*, Belinda Gargiulo ^1^, LauraPanicali ^2^, Rosa Palumbo ^1^, Antonella Rossini ^1^, Melissa Ballarini ^1^ and Roberta Toschi ^1^

1 AUSL Bologna, Italy
2 AOSP Bologna, Italy

* Correspondence: bianca.asbusti@gmail.com
**Abstract**
*Background/Objectives:* The management of patients on the renal transplant waiting list requires timely clinical monitoring and documentation. Six-monthly reporting may delay updates and affect transplant readiness. This intervention aims to improve timeliness and continuity of care through a real-time document management system, integrated with the Primary Nursing model. *Methods*: Primary Nursing has operationalized the model through the role of the Primary Nurse, who: (1) He collaborates with the doctor for a systematic review of the reports. (2) Identify relevant data for the transplant pathway at an early stage. (3) Coordinate secure document transmission. *Results*: The introduction of the model has determined: Greater responsiveness in the management of clinical events; Constant and accurate updating documentation; Reduction of verification times by the Transplant Center; More fluid and effective communication between the Dialysis Center and the Transplant Center; Central role of the Primary Nurse. Criticality: high commitment to continuous monitoring. Strategy: development of a computer program for the direct and secure sharing of reports. *Conclusions*: Experience shows that a multiprofessional model integrated with Primary Nursing increases the efficiency of the transplant pathway, improving timeliness, documentary accuracy, and patient safety. The Primary Nurse represents a key figure for continuity, personalization, and coordination of the process.
**Keywords:** primary nursing; renal transplantation; dialysis

**SC24. Nursing Care Bundle for the Prevention of Vascular Access Dysfunction in Hemodialysis: Proposal for a Pilot Project**
Chiara Lo Presti ^1,^*, Alessandra Cassia ^1,2^, Giada Dimaruro ^1,2^, Laila Dimauro ^1,2^, Miriam Lozito ^1,2^, Alessio Sebastiano Vita ^1,2^, Antonino Vittorio ^1,2^, Marilù Correnti ^1^, Salvatore Floridia ^1,2^, Giovanni Gianni ^1^, Ludovica Renzelli ^1^ and Tito Gianni ^1^

1 Servizio Nefrodialitico Tike S.r.l—Ambulatori di Siracusa e Palazzolo A., Siracusa, Italy
2 Ambulatorio di Emodialisi Floridiano., Floridia, Siracusa, Italy

* Correspondence: infloprestichiara@gmail.com
**Abstract**
*Background/Objectives:* Vascular access dysfunction is one of the main complications in patients undergoing maintenance hemodialysis, with significant clinical and organizational impact. A systematic review by Yao et al. identified significant predictors of arteriovenous fistula (AVF) dysfunction. Although the available evidence has limitations, these factors provide a useful framework for identifying at-risk clinical domains. In parallel, the Italian National Federation of Nursing Professions promotes care bundles as tools to standardize care and improve patient safety. This study proposes a structured nursing care bundle and defines a pilot project for implementation in hospital and outpatient hemodialysis settings. *Methods:* Predictive factors identified by a previous study, were translated into four integrated nursing interventions: structured patient education; diabetes management and glycemic monitoring; prevention of intradialytic hypotension; and biochemical monitoring with standardized anticoagulant use according to protocol. A fifth intervention, clinical vascular access surveillance, was added according to KDOQI 2019 recommendations. The proposed pilot project includes baseline data collection, bundle implementation, and pre/post evaluation through process and outcome indicators. Expected *Results*: The bundle is expected to translate predictive factors into standardized interventions, reduce variability in care, and support early identification of access dysfunction, potentially decreasing thrombotic events and improving continuity of care. *Conclusions*: A structured nursing care bundle represents an innovative application of the care bundle methodology in nephrology, promoting evidence-based, replicable, and measurable practice.
**Keywords:** care bundle; vascular access; nephrology nursing; hemodialysis; patient safety

**SC25. Impact of Primary Nursing Model Implementation on Clinical and Organizational Outcomes in Hemodialysis: A Pre-Post Study Protocol in a Northern Italian Local Health Authority**
Andrea Contini ^1^, Roberta Barbieri ^1^, Monica Muroni ^1^, Gaetana Maria Droghi ^1^, Lorella Cappucciati ^1^, Martina Maserati ^1^, Enrica Cordani ^2^, Cristina Scaglia ^2^ and Gaetano Gallo ^2,^*

1 Dipartimento delle Professioni Sanitarie e Sociali, Azienda Unità Sanitaria Locale of Piacenza, Piacenza, Italy
2 U.O.C. Nefrologia e Dialisi, Azienda Unità Sanitaria Locale of Piacenza, Piacenza, Italy

* Correspondence: g.gallo@ausl.pc.it
**Abstract**
*Background/Objectives:* Hemodialysis care is characterized by high clinical and organizational complexity, requiring models that ensure continuity of care, professional accountability, and patient-centeredness. Task-based models, although widely adopted, may lead to fragmented care delivery and reduced perceived quality among both patients and healthcare professionals. The Primary Nursing model emphasizes continuity, accountability, and individualized care. This study aimed to evaluate the impact of Primary Nursing model implementation on the nursing work environment and patient-reported quality of life in hemodialysis settings. *Methods*: A prospective, single-center, pre–post observational study will be conducted in the Nephrology and Dialysis Unit of the Piacenza Local Health Authority, organized into one hub and three spoke centers. The intervention consists of the phased implementation of the Primary Nursing model, initially introduced in lower-complexity settings and subsequently extended to the hub center. Nurses and hemodialysis patients will be enrolled according to predefined inclusion and exclusion criteria. The nursing work environment will be assessed using the Practice Environment Scale of the Nursing Work Index (PES-NWI), while patient quality of life will be measured using the Kidney Disease Quality of Life-36 (KDQOL-36). Continuity of care will be evaluated as a process indicator by measuring the proportion of dialysis sessions delivered by the assigned primary nurse. Data will be collected at baseline and at 12 months. Statistical analysis will include paired tests with a significance level of *p* < 0.05. *Expected Results*: The intervention is expected to improve the nursing work environment and patient-reported outcomes, particularly quality of life and continuity of care. *Conclusions*: Findings may inform organizational strategies and support the scalability of the model in complex nephrology settings.
**Keywords:** primary nursing; hemodialysis; organizational models

**SC26. Standardizing Nutritional Competencies in Nephrology Nursing: A Scoping Review**
Gaetano Ferrara ^1,2^*, Sara Morales Palomares ^2,3^, Domenico Cannizzaro ^2,4^, Sofia Matteucci ^2,5^, Giovanni Cangelosi ^2,6^, Addolorata Palmisano ^2^, Stefano Mancin ^2^ and Marco Sguanci ^2^

1 Department of Biomedicine and Prevention, University of Rome “Tor Vergata”, Via Montpellier 1, 00133 Rome, Italy
2 Italian Society of Nephrology Nurses (SIAN), Via Capotesta 1/30, 07026 Olbia, Italy
3 Department of Pharmacy, Health and Nutritional Sciences (DFSSN), University of Calabria, Via Pietro Bucci, 87036 Rende, Cosenza, Italy
4 University of Modena and Reggio Emilia (UNIMORE), Via Università 4, 41121 Modena, Italy
5 IRCCS Humanitas Clinical and Research Hospital, Via Manzoni 56, 20089 Rozzano, Milano, Italy
6 School of Pharmacy, Experimental Medicine and Public Health Campus, University of Camerino, Via Madonna delle Carceri 9, 62032 Camerino, Macerata, Italy

* Correspondence: gaetano.ferrara@students.uniroma2.eu
**Abstract**
*Background/Objectives*: Chronic kidney disease (CKD) is frequently associated with malnutrition and protein-energy wasting, contributing to adverse clinical outcomes and reduced quality of life. Nephrology nurses play a pivotal role in nutritional care; however, their competencies remain heterogeneous, and standardized frameworks are lacking across the continuum of care. This review aimed to map the available evidence on the nutritional competencies of nephrology nurses in the care of adults with CKD. *Methods*: A scoping review was conducted following the Joanna Briggs Institute (JBI) methodology and reported according to the PRISMA-ScR guidelines. The search strategy was guided by the Population–Concept–Context (PCC) framework and focused on nephrology nurses, nutritional competencies, and nephrology care settings. A comprehensive literature search was conducted in September 2025 across PubMed/MEDLINE, Embase, CINAHL, Scopus, Web of Science, the Cochrane Library, and grey literature sources, including Google Scholar. *Results*: Of the 4171 records identified, 17 studies published between 2010 and 2025 were included. Nutritional competencies in nephrology nursing emerged as a multidimensional construct encompassing four domains: educational-communicative, advanced clinical, collaborative-professional, and general. Structured nursing interventions were associated with improved dietary adherence, earlier identification of malnutrition risk, and enhanced continuity of care. Nevertheless, important educational gaps and the absence of standardized competency frameworks persist. *Conclusions*: Nutritional competencies are critical to the quality and safety of nephrology care. The development of shared competency frameworks and targeted educational programs is essential to standardize clinical practice and strengthen the nursing role in the nutritional management of CKD.
**Keywords:** chronic kidney disease; nutritional competence; nephrology nursing; malnutrition

**SC27. Clinical experience with respiratory syncytial virus vaccination in patients undergoing haemodialysis: the key role of the nurse**
Flavia Marinotti ^1,^*, Claudia Miccoli ^2^, Giuseppe Scaparrotta ^3^, Nicola Celon ^4^ and Federico Nalesso ^4^

1 Nefrologia2 -Sezione trattamenti, Azienda Ospedale Università di Padova, Via Giustiniani 1, Padua, Italy
2 Nefrologia2 -Sezione trattamenti, Azienda Ospedale Università di Padova, Via Giustiniani 1, Padua, Italy
3 Nefrologia2 -Sezione trattamenti, Azienda Ospedale Università di Padova, Via Giustiniani 1, Padua, Italy
4 UOC Nefrologia, Dialisi e Trapianto, Azienda Ospedale Università di Padova, Via Giustiniani 1, Padua, Italy; Director school of Nephrology, University of Padua, Via VII Febbraio 2, Padua, Italy

* Correspondence: flaviamarinotti@gmail.com
**Abstract**
*Background/Objectives:* In patients with chronic kidney disease (CKD) undergoing haemodialysis, vaccination is essential due to immunosuppression and a high risk of infection. Respiratory syncytial virus (RSV) causes significant respiratory infections, which are often severe and require hospitalisation. Objective: to identify patients suitable for RSV vaccination and to assess their immune response and clinical outcomes for up to 12 months. *Methods*: Out of a total of 104 haemodialysis patients, 28 were considered eligible based on the inclusion criteria: age ≥ 60 years and the presence of comorbidities such as CKD, chronic lung disease, diabetes mellitus and heart disease. Following the provision of the information sheet and the obtaining of informed consent, and after ruling out hypersensitivity to the excipients, the injection site was selected, the vaccine was administered, and the patient was monitored for approximately 20 min. The immune response was assessed by serological measurement of IgG and IgA immunoglobulins. *Results*: The antibody test indicated a negative result in 57.1% of patients, a positive result in 25.0% and an inconclusive result in 17.9%. Five patients also received the flu vaccine, and one was a transplant patient. During the 12-month follow-up period, one case of asthmatic bronchitis and one case of bronchitis were documented. *Conclusions*: RSV vaccination in patients on haemodialysis was feasible in this cohort and was associated with a low observed incidence of respiratory events during the follow-up period, suggesting a potential clinical benefit in this high-risk population.
**Keywords:** haemodialysis; vaccination; RSV

**SC28. The “Grey Zone” of Fluid Balance: Nursing Knowledge on Perspiratio Insensibilis and Usability of the CareBalance-N App. A Cross-Sectional Observational Study**
Anna Grimaldi ^1^, Vincenzo Di Nuzzo ^2^, Giuseppe Frattolillo ^1^, Domenico Di Sivo ^3^, Giovanni Gavarro ^1^, Incoronata Chiusolo ^4^ and Ciro Pozzuoli ^5^

1 Intensive Care Unit, Marcianise Hospital, Caserta Local Health Authority (ASL Caserta) Viale Sossietta Scialla, 81025 Marcianise, Caserta, Italy;
2 Emergency Department, Marcianise Hospital, Caserta Local Health Authority (ASL Caserta), Viale Sossietta Scialla, 81025 Marcianise Caserta, Italy;
3 Congenital Heart Disease Unit, Monaldi Hospital, Azienda Ospedaliera dei Colli, Via Leonardo Bianchi, 80131 Naples, Italy;
4 University Hospital of Padua, Via Nicolò Giustiniani 2, 35128 Padua, Italy
5 General Surgery Unit, Marcianise Hospital, Caserta Local Health Authority (ASL Caserta), Viale Sossietta Scialla, 81025 Marcianise, Caserta, Italy

* Correspondence: anna.grimaldi92a@gmail.com
**Abstract**
*Background/Objectives*: Fluid balance management is a key nursing competency, but interpretation remains variable due to non-measurable components such as perspiratio insensibilis, relevant for clinical decision-making, especially in nephrology. Digital tools may improve standardization. This study assesses nursing knowledge, and evaluates the usability, acceptability, and potential integration of the CareBalance-N app across care settings. *Methods*: A cross-sectional descriptive observational study was conducted on 50 nurses working in dialysis, internal medicine, and intensive care units. A 25-item knowledge questionnaire (score 0–100) was administered, and an app mockup was evaluated using the User version of the Mobile Application Rating Scale (uMARS), a validated tool. Reporting followed STROBE recommendations. The app integrates a fluid balance calculator and an indicative estimate of *perspiratio insensibilis* based on body weight and clinical correction factors. *Results*: Knowledge scores were moderate (67.6 ± 7.7), with higher levels observed in nephrology compared to other settings. The app demonstrated good usability (uMARS 3.92 ± 0.16). A positive association emerged between knowledge levels and app evaluation, suggesting greater appreciation among more knowledgeable nurses. *Conclusions*: Perspiratio insensibilis represents a “grey zone” in nursing fluid balance assessment. CareBalance-N demonstrates good usability and may support continuity and interpretative consistency among professionals across different care settings.
**Keywords:** fluid balance; new technologies; nursing care

**SC29. Beyond the dressing: what is the impact of central venous catheter visibility in hemodialysis patients?**
Silvia Barcaro ^1,^*, Mariangela Mettifogo ^1^ and Laura Gattera ^2^

1 U.O.C. Nefrologia, Azienda ULSS 8 Berica, Vicenza, Italy
2 U.O.C. Nefrologia, Azienda ULSS 8 Berica, sede di Valdagno, Italy

* Correspondence: silvia.barcaro@aulss8.veneto.it
**Abstract**
*Background/Objectives:* Satisfaction with vascular access among hemodialysis patients is associated with improved quality of life and reduced rates of depression and mortality. Recent research on Peripherally Inserted Central Venous Catheters (PICCs) suggests that certain patients report improved body image when utilizing covering dressings. This study evaluates whether satisfaction with Central Venous Catheter (CVC) access varies significantly between occlusive and transparent dressings. *Methods*: The validated Short Form-Vascular Access Questionnaire (SF-VAQ) was administered to CVC patients over a one-week period at the hemodialysis centers of Vicenza, Arzignano, and Valdagno. Statistical analyses were performed using Shapiro-Wilk, Mann-Whitney U, Spearman’s Rho, and Kruskal-Wallis tests. *Results*: The sample comprised 48 subjects (median age: 71.1 years), with 58% utilizing transparent dressings. While overall satisfaction was not significantly associated with dressing type, covering dressings were linked to increased interference with daily living, concerns regarding access longevity, and anxiety over potential hospitalization due to malfunction. Notably, male gender, younger age, and active employment status were significantly correlated with lower satisfaction levels. *Conclusions*: Transparent dressings are recommended due to their minimal impact on daily activities and aesthetic perception. Systematic assessment of patient experience is essential in hemodialysis units to facilitate the disclosure of concerns and expectations, particularly among younger and employed populations. Future research should explore the link between vascular access satisfaction and treatment adherence to advance patient-centered care models.
**Keywords:** CVC; SF-VAQ; dressing

**SC30. A medical humanities-based research protocol for developing the legal knowledge of nephrology nurses**
Stefania Meo ^1,2^, Immacolata de Simone ^2,3^ and Marisa Pegoraro ^2,^*

1 Grande Ospedale Metropolitano Niguarda Milan Italy;
2 Palliative Care Study Group, Italian Society of Nephrology Nurses (SIAN), Via Capotesta 1/30, 07026 Olbia, Italy
3 Ospedale Maggiore Policlinico Milan, Milan, Italy

* Correspondence: marisapegoraro.996@gmail.com
**Abstract**
*Background/Objectives:* In the context of advanced chronic kidney disease (CKD), nurses are exposed to highly complex clinical, ethical, and emotional situations, particularly in advanced illness and at the end of life. This scenario highlights moral distress, a form of persistent ethical suffering associated with burnout and reduced quality of care. The phenomenon may be linked to insufficient training in communication, ethical, and legal competencies. These observations are supported by the recent national survey conducted by Dalla Gassa and by the “Thinking-Writing” workshop of this Working Group. This study aims to increase the legal and legislative knowledge needed for effective communication and patient advocacy and to support nurses in their role as mediators and facilitators of decision-making processes. *Methods*: This multicenter prospective study will provide experiential training interventions using medical humanities and narrative medicine, together with clinical audits designed to stimulate ethical reflection on dilemmas arising from cases presented by trainers and participants. The training will include online meetings via Zoom with 20 volunteer nurses recruited through SIAN over 6 months, for a total of 24 h of CME-accredited training. The following tools will be used to evaluate the intervention: (I) a legal and legislative knowledge survey; (II) a moral distress scale; and (III) a semi-structured interview exploring self-perception. *Expected Results*: An increase in legal knowledge after training is expected. *Conclusions*: Training based on medical humanities and narrative medicine represents a promising educational approach that can combine theoretical knowledge, ethical reflection, and lived experience. Further experimental studies will be needed to evaluate long-term effectiveness and its contribution to care models that respect the dignity of the individual.
**Keywords:** legal knowledge; moral distress; medical humanities

**SC31. Post-Dialysis Fatigue in the adult population: analysis of predictive factors and impact on Quality of Life. A cross-sectional observational study**
Giacinto Fabio Caruso, Mariachiara Figura, Giuseppe D’Anna and Roberto Latina
Department of Maternal and Child Health Promotion, Internal Medicine & Specialties of Excellence “G. D’Alessandro” (PROMISE), University of Palermo, Palermo, Italy; giacintofabio.caruso@policlinico.pa.it
**Abstract**
*Background/Objectives:* In the context of MRCA, nurses are exposed to highly complex clinical, ethical, and emotional situations, particularly in the advanced stages of illness and at the end of life. This scenario highlights the phenomenon of moral distress, persistent ethical suffering, associated with burnout and a reduction in the quality of care. This is a widespread and systematic phenomenon, linked to insufficient training in communication, ethical, and legal skills. This data is supported by the recent national survey conducted by Dalla Gassa and the “Thinking-Writing” workshop of this Working Group. Increase the legislative knowledge needed for effective communication, patient advocacy, and support nurses in their role as mediators and facilitators of decision-making processes. *Methods*: The experimental, multicenter, and prospective research offers experiential training interventions using medical humanities and narrative medicine, along with clinical audits to stimulate ethical questions and dilemmas based on cases presented by the trainers and participants themselves. The training includes online meetings via the Zoom platform with 20 nurses selected on a voluntary basis from SIAN for a duration of 6 months, for a total of 24 h of CME-accredited training. The following tools will be used to measure the benefits of the training interventions: (I) Legal and legislative knowledge survey; (II) Moral distress scale; (III) Semi-structured interview to explore self-perception. *Results*: We expect an increase in legal knowledge after training. *Conclusions*: Training based on medical humanities and narrative medicine represents an innovative and promising response, capable of combining theoretical knowledge, ethical reflection, and lived experience. Further experimental studies will be needed to evaluate its long-term effectiveness and contribute to the development of more humane care models that respect the dignity of the individual.
**Keywords:** legal knowledge; moral distress; medical humanities

**SC32. AUDIT 2025: Ultrasound-Guided AVF Puncture—Guidelines and Recommendations. Analysis of 2 years experience.**
Pasquale Pellicanò, Dario Pazzano, Maurizio Cozzupoli, Romeo Angelo, Antonino Caserta and Antonia Putortì
U.O.C. Nefrologia Dialisi abilitata al Trapianto di Rene del Grande Ospedale Metropolitano “Bianchi-Melacrino-Morelli”, Reggio Calabria, Italy; pasqualepellicano@icloud.com
**Abstract**
*Background/Objectives:* Efficient vascular access is mandatory for an adequate hemodialysis treatment. The arteriovenous fistula (AVF) remains the gold standard for vascular access, but an aging and increasingly complex patient population represents a critical challenge to its maintenance. International guidelines recommend the use of ultrasound in cases of difficult vascular access to improve safety and effectiveness during cannulation. *Methods:* As part of a periodic clinical audit program within our Unit, we evaluated the number of patients with AVFs, how many underwent ultrasound-guided puncture, and the reasons for its use. The January–September periods of 2024 and 2025 were selected to compare the adoption, effectiveness, and safety of the technique between the two years. *Results:* In 2024, 23 out of 63 patients (36%) received ultrasound-guided AVF punctures; in 2025, this increased to 24 out of 54 patients (44%, *p* = 0.38). Although the year-to-year increase was not statistically significant, it aligns with current literature recommendations. The success rate of ultrasound-guided punctures was very high, exceeding 95% in both years, confirming the method’s effectiveness and safety. *Conclusions:* Our findings are consistent with meta-analyses and systematic reviews, confirming that ultrasound-guided AVF puncture is a valuable tool for managing difficult and complex vascular access. Structured implementation can improve success rates and reduce complications compared with traditional techniques. Continuous training and standardization of the method are essential. The use of dedicated procedural documentation should become routine, contributing to improved quality of dialysis care.
**Keywords:** arteriovenous fistula (AVF); ultrasound-guided puncture; vascular access; hemodialysis; clinical audit

**SC33. Nursing Case Management and Care Coordination in the Evaluation of Living Kidney Donors: A Scoping Review**
Anna Grimaldi ^1^, Ciro Pozzuoli ^2^, Giuseppe Frattolillo ^1^, Domenico Di Sivo ^3^, Vincenzo Di Nuzzo ^4^ and Incoronata Chiusolo ^5,^*

1 Intensive Care Unit, Marcianise Hospital, Caserta Local Health Authority (ASL Caserta), Viale Sossietta Scialla, 81025 Marcianise, Caserta, Italy
2 General Surgery Unit, Marcianise Hospital, Caserta Local Health Authority (ASL Caserta), Viale Sossietta Scialla, 81025 Marcianise, Caserta, Italy
3 Congenital Heart Disease Unit, Monaldi Hospital, Azienda Ospedaliera dei Colli, Via Leonardo Bianchi, 80131 Naples, Italy
4 Emergency Department, Marcianise Hospital, Caserta Local Health Authority (ASL Caserta), Viale Sossietta Scialla, 81025 Marcianise, Caserta, Italy;
5 University Hospital of Padua, Via Nicolò Giustiniani 2, 35128, Padua, Italy

* Correspondence: renata91rc@gmail.com
**Abstract**
*Background/Objectives:* Living kidney donation is associated with better clinical outcomes and shorter waiting times compared with deceased donor transplantation, contributing substantially to global transplant activity. However, the evaluation pathway for living donors is complex, multidisciplinary, and potentially fragmented, with implications for donor-centered outcomes. In this context, nursing case management and care coordination represent advanced organizational strategies based on specialized nephrology nursing competencies. Nevertheless, their specific role in the pre-transplant evaluation of living kidney donors has not been systematically mapped. This review aimed to map the literature on nursing case management and care coordination interventions in the evaluation pathway of adult living kidney donors, describing their characteristics, outcomes, and implementation factors. *Methods*: A scoping review was conducted following the Arksey and O’Malley methodological framework and reported in accordance with PRISMA-ScR guidelines. Literature searches were performed in PubMed/MEDLINE, CINAHL, Scopus, and Embase. Qualitative, quantitative, mixed-methods studies and organizational reports were included if they addressed nursing coordination interventions in the evaluation process of adult living donors. Data were synthesized using descriptive analysis and thematic categorization. *Results*: Identified interventions included pathway planning and monitoring, facilitation of multidisciplinary communication, structured education, and longitudinal care coordination. Reported outcomes encompassed improved perceived continuity of care, reduced evaluation time, increased donor satisfaction, and lower emotional burden. *Conclusions*: Nursing case management and care coordination emerge as strategic approaches to optimize the evaluation pathway of living kidney donors, enhancing efficiency and supporting a more integrated, donor-centered care process.
**Keywords:** case management; kidney transplantation; scoping review

**SC34. Experiences and Practices of Nephrology Nurses in the Nutritional Care of Patients with Chronic Kidney Disease: A Qualitative Study**
Pasquale Di Fronzo ^1^, Sofia Matteucci ^2^, Gaetano Ferrara ^3,^*, Anna Grimaldi ^4^, Stefano Mancin ^1^ and Marco Sguanci ^1^

1 Italian Society of Nephrology Nurses (SIAN), Via Capotesta 1/30, 07026 Olbia, Italy
2 IRCCS Humanitas Research Hospital, Via Manzoni 56, 20089 Rozzano, Milan, Italy
3 Department of Biomedicine and Prevention, University of Rome “Tor Vergata”, Via Montpellier 1, 00133 Rome, Italy
4 Intensive Care Unit, Marcianise Hospital, Caserta Local Health Authority (ASL Caserta), Viale Sossietta Scialla, 81025 Marcianise, Caserta, Italy

* Correspondence: gaetano.ferrara@students.uniroma2.eu
**Abstract**
*Background/Objectives*: Chronic Kidney Disease (CKD) requires complex and continuous nutritional management. In this context, nutrition represents a core component of care, where nephrology nurses play a key role in patient education, support, and promotion of therapeutic adherence. This study aimed to explore nurses’ perceptions and nutritional competencies in managing patients with CKD in daily clinical practice. *Methods*: A descriptive qualitative study was conducted using semi-structured interviews with 22 nephrology nurses (October–December 2025). Data were analyzed through thematic analysis following Braun and Clarke’s approach. Study conduct and reporting adhered to the EQUATOR Network guidelines, specifically the 32-item Consolidated Criteria for Reporting Qualitative Research (COREQ). *Results*: Thematic analysis identified six main themes: (1) nutritional competencies and professional nursing identity, highlighting gaps in prior education and the need for further training; (2) provision of specific nutritional information, including structured and personalized education on diet and fluid intake; (3) collaboration with other healthcare professionals, emphasizing the importance of multidisciplinary teamwork; (4) experiential learning, through on-the-job training and self-directed learning; (5) care-related challenges and patient characteristics, including issues with adherence and generational and socio-cultural differences; (6) nutritional assessment, involving clinical-instrumental evaluation and the use of assessment tools. *Conclusions*: Nutrition is a central component in the management of patients with CKD; however, significant educational gaps among nurses persist. Adequately trained nurses could effectively influence patients’ dietary habits. Interdisciplinary collaboration remains limited, and nutritional management is affected by cultural and communication barriers, although opportunities for future development and collaboration exist.
**Keywords:** chronic kidney disease; nutrition therapy; nephrology nursing

**SC35. Perceived Frailty and Quality of Life in Kidney Transplant Recipients: A Systematic Review**
Domenico Di Sivo ^1,^*, Anna Grimaldi ^2^, Vincenzo Di Nuzzo ^3^, Giuseppe Frattolillo ^2^, Ciro Pozzuoli ^4^, Giovanni Gavarro ^2^ and Incoronata Chiusolo ^5^

1 Congenital Heart Disease Unit, Monaldi Hospital, Azienda Ospedaliera dei Colli, Via Leonardo Bianchi, 80131 Naples, Italy
2 Intensive Care Unit, Marcianise Hospital, Caserta Local Health Authority (ASL Caserta) Viale Sossietta Scialla, 81025 Marcianise, Caserta, Italy
3 Emergency Department, Marcianise Hospital, Caserta Local Health Authority (ASL Caserta), Viale Sossietta Scialla, 81025 Marcianise, Caserta, Italy
4 General Surgery Unit, Marcianise Hospital, Caserta Local Health Authority (ASL Caserta), Viale Sossietta Scialla, 81025 Marcianise, Caserta, Italy
5 University Hospital of Padua, Via Nicolò Giustiniani 2, 35128 Padua, Italy

* Correspondence: disivo.domenico1@gmail.com
**Abstract**
*Background/Objectives:* Kidney transplantation is the treatment of choice for end-stage renal disease; however, clinical and psychosocial vulnerabilities may persist during follow-up and negatively affect outcomes. Frailty, a known predictor of mortality and hospitalization in nephrology, can also be assessed through patient-reported measures, complementing traditional clinical indicators within a person-centered care approach. This review aimed to synthesize evidence on perceived frailty in adult kidney transplant recipients and its association with quality of life and clinical factors. *Methods*: A systematic review was conducted according to PRISMA guidelines. Searches were performed in MEDLINE, Embase, Scopus, CINAHL, and ClinicalTrials.gov, including grey literature. Observational studies on adult kidney transplant recipients assessing perceived frailty via self-reported tools or PROMs were included. Two independent reviewers conducted study selection and data extraction. Risk of bias was assessed using the Newcastle–Ottawa Scale and JBI checklist, and evidence certainty was evaluated with the GRADE approach. *Results*: Included studies reported variable prevalence of perceived frailty due to differences in populations, tools, and designs. Higher frailty was associated with greater comorbidity and longer pre-transplant dialysis. It was consistently linked to poorer quality of life (physical and psychosocial), increased depressive symptoms, reduced functional autonomy, and higher healthcare utilization. Self-reported instruments captured vulnerabilities not always identified by clinical indices, although heterogeneity limited comparability. *Conclusions*: The systematic assessment of perceived frailty may support a more comprehensive and person-centered follow-up in kidney transplant recipients, highlighting the contribution of nephrology nursing in long-term care management.
**Keywords:** kidney transplantation; frailty; systematic review

**SC36. PHOS-BALANCE-QI: Quality Improvement of a Structured Educational Intervention on Hyperphosphatemia in Hemodialysis Patients**
Incoronata Chiusolo ^1,^*, Ciro Pozzuoli ^2^, Giuseppe Frattolillo ^3^, Domenico Di Sivo ^4^, Vincenzo Di Nuzzo ^5^, Gaetano Ferrara ^6^ and Anna Grimaldi ^3^

1 University Hospital of Padua, Via Nicolò Giustiniani, 2, 35128, Padua, Italy
2 General Surgery Unit, Marcianise Hospital, Caserta Local Health Authority (ASL Caserta), Viale Sossietta Scialla, 81025, Marcianise, Caserta, Italy
3 Intensive Care Unit, Marcianise Hospital, Caserta Local Health Authority (ASL Caserta), Viale Sossietta Scialla, 81025 Marcianise, Caserta, Italy
4 Congenital Heart Disease Unit, Monaldi Hospital, Azienda Ospedaliera dei Colli, Via Leonardo Bianchi, 80131 Naples, Italy
5 Emergency Department, Marcianise Hospital, Caserta Local Health Authority (ASL Caserta), Viale Sossietta Scialla, 81025 Marcianise, Caserta, Italy
6 Nephrology and Dialysis Unit, Ramazzini Hospital, Via Guido Molinari, 2, 41012 Carpi, Italy

* Correspondence: renata91rc@gmail.com
**Abstract**
*Background/Objectives:* Hyperphosphatemia in hemodialysis patients is associated with vascular calcification, increased cardiovascular risk, and reduced quality of life. Phosphorus from food additives, which is highly absorbable and often underrecognized, is a key challenge in dietary management. Structured, personalized educational interventions may improve nutritional control in chronic kidney disease. This project aims to standardize multiprofessional counseling and evaluate the impact of an educational brochure on added phosphates on serum phosphorus control, chelator adherence, and self-efficacy in hemodialysis patients. *Methods*: This quality improvement project follows the Plan–Do–Study–Act cycle and Lean principles to standardize nutritional counseling. Adult hemodialysis patients with phosphorus >5.0 mg/dL on two consecutive checks will be included. The intervention will consist of a structured 10–15-min educational session using a checklist and brochure, with reinforcement at 2 and 4 weeks through teach-back, focusing on phosphorus sources, label reading, and correct chelator use. Primary outcomes are a phosphorus reduction of ≥0.8 mg/dL or ≥15% and an increase in the proportion of patients within the target of ≤5.5 mg/dL at 12 weeks; secondary outcomes are treatment adherence and self-efficacy. *Expected Results:* A clinically meaningful improvement in serum phosphorus control is expected, with lower mean values and a higher proportion of patients within target. The intervention is also expected to increase awareness of phosphorus sources, especially additives, support appropriate chelator use and adherence, and improve self-efficacy. Standardizing counseling may reduce variability among providers and improve intervention sustainability and replicability. *Conclusions*: The project proposes a structured, measurable, and replicable educational model for dialysis centers that may provide a basis for prospective controlled studies.
**Keywords:** hyperphosphatemia; hemodialysis; quality improvement

**SC37. The PLAY-KID Study (Preventive Learning Activity for Kidney Injury Detection): implementation of a gamified educational strategy for renal prevention in community nursing—a mixed-methods Quality Improvement protocol**
Vincenzo Di Nuzzo ^1,^*, Giuseppe Frattolillo ^2^, Ciro Pozzuoli ^3^, Incoronata Chiusolo ^4^, Domenico Di Sivo ^5^ and Anna Grimaldi ^2^

1 Emergency Department, Marcianise Hospital, Caserta Local Health Authority (ASL Caserta), Viale Sossietta Scialla, 81025 Marcianise, Caserta, Italy
2 Intensive Care Unit, Marcianise Hospital, Caserta Local Health Authority (ASL Caserta) Viale Sossietta Scialla, 81025 Marcianise, Caserta, Italy
3 General Surgery Unit, Marcianise Hospital, Caserta Local Health Authority (ASL Caserta), Viale Sossietta Scialla, 81025 Marcianise, Caserta, Italy
4 University Hospital of Padua, Via Nicolò Giustiniani, 2, 35128 Padua, Italy
5 Congenital Heart Disease Unit, Monaldi Hospital, Azienda Ospedaliera dei Colli, Via Leonardo Bianchi, Naples, Italy

* Correspondence: vincenzo.dinuzzo1998@gmail.com
**Abstract**
*Background/Objectives:* In primary care settings, prevention of acute kidney injury (AKI) is a clinical priority, especially for frail patients. However, the high workload of community nurses may make traditional training programs difficult to integrate. Gamification may offer an innovative strategy to support learning and self-efficacy through active engagement and positive reinforcement. The primary objective is to evaluate the impact of the PLAY-KID intervention on nurses’ knowledge of renal risk prevention. Secondary objectives include perceived self-efficacy, participant engagement (completion rates), satisfaction, and contextual factors (barriers and facilitators) influencing implementation. *Methods:* A mixed-methods quality improvement (QI) design with a convergent approach will be adopted over 5–6 weeks. A convenience sample of 40 community nurses will participate in a digital asynchronous micro-learning program consisting of four weekly modules based on micro-clinical cases, quizzes, and immediate feedback. Quantitative data (pre-post tests and self-efficacy scales) and qualitative data (focus groups) will be integrated using a joint-display framework. *Expected Results:* The intervention is expected to improve knowledge and perceived self-efficacy while achieving satisfactory engagement and acceptability; qualitative findings will identify barriers and facilitators to implementation. *Conclusions:* The PLAY-KID study will evaluate gamified micro-learning as a potentially sustainable and scalable tool for continuous quality improvement in primary care and professional training in high-intensity work contexts.
**Keywords:** acute kidney injury (aki); community nursing; gamification; quality improvement; mixed methods; micro-learning

**SC38. Self-efficacy of Nursing Students in the Management of Patients Undergoing Dialysis and CRRT: A Scoping Review According to PRISMA**
Artiola Gaetano, Stefanini Emmanuele, Antonio De Biase, Serena Caparroti and Giuseppe de Falco
Università degli Studi della Campania “Luigi Vanvitelli”, polo Didattico AO dei Colli, Napoli, Italy; gart1587@gmail.com
**Abstract**
*Background/Objectives*: Nursing management of patients undergoing hemodialysis, peritoneal dialysis, and continuous renal replacement therapy (CRRT) requires advanced technical and decision-making skills. Self-efficacy is a key determinant of clinical performance, influencing safety, autonomy, and quality of care. However, its development within nephrology education remains insufficiently explored. *Methods*: A scoping review was conducted according to JBI methodology and PRISMA-ScR guidelines. The PCC framework included nursing students (Population), clinical self-efficacy (Concept), and nephrology settings (Context). A systematic search was performed in PubMed/MEDLINE, Embase, CINAHL, ERIC, and Scopus (2016–2026). Quantitative, qualitative, and mixed-methods studies were included. Screening was conducted by two independent reviewers, with standardized data extraction and narrative synthesis. *Results*: The literature is limited and heterogeneous. Main educational strategies include high-fidelity simulation, clinical experiential training, and structured mentorship. These interventions are associated with increased perceived competence, confidence, and clinical readiness. However, there is a lack of standardized tools to measure self-efficacy. *Conclusions*: Self-efficacy appears underestimated in nephrology education. Structured, simulation-based educational models with systematic evaluation are essential to bridge the theory–practice gap. Further robust studies and standardized interventions are needed to strengthen advanced competencies and improve care outcomes.
**Keywords:** nursing students; self-efficacy; dialysis and crrt

**SC39. Therapeutic Adherence and Self-Management in Kidney Transplant Recipients: A Systematic Review of Randomized Controlled Studies**
Domenico Di Sivo ^1,^*, Anna Grimaldi ^2^, Vincenzo Di Nuzzo ^3^, Giuseppe Frattolillo ^2^, Ciro Pozzuoli ^4^, Giovanni Gavarro ^2^ and Incoronata Chiusolo ^5^

1 Congenital Heart Disease Unit, Monaldi Hospital, Azienda Ospedaliera dei Colli, Via Leonardo Bianchi, 80131 Naples, Italy
2 Intensive Care Unit, Marcianise Hospital, Caserta Local Health Authority (ASL Caserta) Viale Sossietta Scialla, 81025 Marcianise, Caserta, Italy
3 Emergency Department, Marcianise Hospital, Caserta Local Health Authority (ASL Caserta), Viale Sossietta Scialla, 81025 Marcianise, Caserta, Italy
4 General Surgery Unit, Marcianise Hospital, Caserta Local Health Authority (ASL Caserta), Viale Sossietta Scialla, 81025 Marcianise, Caserta, Italy
5 University Hospital of Padua, Via Nicolò Giustiniani 2, 35128 Padua, Italy

* Correspondence: disivo.domenico1@gmail.com
**Abstract**
*Background/Objectives:* Kidney transplantation is the treatment of choice for end-stage renal disease; however, long-term graft survival depends on adherence to immunosuppressive therapy. Non-adherence is associated with rejection and graft loss, with reported rates varying due to differences in measurement methods. Nursing plays a key role in supporting patient self-management during follow-up. This review aimed to evaluate the effectiveness of nurse-led or nurse-coordinated educational, behavioral, and self-management interventions in improving adherence among adult kidney transplant recipients, and their impact on self-management, quality of life, and clinical outcomes. *Methods*: A systematic review was reported according to PRISMA guidelines. Literature searches were performed in MEDLINE, Embase, Scopus, CINAHL, and ClinicalTrials.gov, supplemented by grey literature. Randomized controlled trials involving adult kidney transplant recipients were included. Risk of bias was assessed using the RoB 2 tool, and certainty of evidence was evaluated using the GRADE approach. Findings were synthesized narratively. *Results*: Nurse-led interventions were generally associated with improved adherence compared with standard care, particularly when based on multicomponent approaches combining education and follow-up. Some studies also reported improvements in self-management and quality of life. However, heterogeneity in intervention design and adherence measures limited comparability, and clinical outcomes were inconsistently reported. *Conclusions*: Nurse-led self-management interventions may improve adherence in kidney transplant recipients. Further high-quality randomized controlled trials with standardized outcome measures and consistent reporting of clinical endpoints are needed.
**Keywords:** kidney transplantation; therapeutic adherence; systematic review

**SC40. Transplant path: clear procedures, small team and great results—An effective organizational model. The ASLCN1 experience**
Alfonso Pecoraro, Rossana Carrera, Erika Naso, Ilaria Serra, Valentina Russo, Ivano Fea, Daniela Omento, Irene Negro, Maura Bosio, Eliana Mandanici, Paola Olivero, Federica Rinaudo, Cristina Fornero, Claudia Mattarozzi and Marita Marengo
S.C. Nephrology and Dialysis, ASLCN1, Cuneo, Italy; alfo.8723@gmail.com
**Abstract**
*Background/Objectives:* The process of entering the transplant waiting list for chronic dialysis patients is complex, requiring multiprofessional coordination, continuity of care, and organizational clarity. This work aims to describe the experience of the Nephrology and Dialysis Unit of Savigliano (ASLCN1, Cuneo, Italy) in implementing an organizational model based on a small, dedicated medical-nursing team and structured operational tools, evaluating its impact on the quality of care. *Methods*: Since 2021, an organizational model featuring a small multiprofessional team dedicated to the transplant path was introduced. This involves periodic meetings to monitor candidate status, schedule diagnostic tests, and coordinate specialist evaluations. Clinical information is shared with the entire healthcare staff to ensure continuity of care. An operational checklist was implemented for managing patients at the time of the transplant call to standardize procedures and support both experienced and newly hired personnel. Effects were evaluated based on the number of transplants, professional coordination, and care safety. *Results*: Between 2021–2025, 28 transplants (14 M; 14 F) were performed, with a progressive annual increase from 3.1% to 6.5% of the total dialysis population. Most patients were on chronic hemodialysis (85.7%), while 10.7% were on peritoneal dialysis. The model improved continuity of care, communication, and likely reduced waiting list entry times. The checklist increased operational safety and reduced the risk of errors, proving particularly useful for new staff. *Conclusions*: Working in a small team is an effective and replicable approach in other nephrological settings. Further developments, such as primary nursing, could further enhance care personalization and the therapeutic relationship.
**Keywords:** pre-transplant; organization; team

**SC41. Management of maxi-emergencies with evacuation protocols in hemodialysis centers: a scoping review**
Mattia Zanon ^1,^*, Nicola Celon ^1^ and Federico Nalesso ^3^

1 Nephrology 2—Dialysis Unit, Azienda Ospedale–Università di Padova, Padua, Italy
2 UOC Nephrology, Dialysis and Kidney Transplantation, Azienda Ospedale–Università di Padova, Padua, Italy
3 School of Nephrology, University of Padua, Padua, Italy

* Correspondence: mattia.zanon8@icloud.com
**Abstract**
*Background/Objectives:* Hemodialysis is a life-saving treatment vulnerable to earthquakes, floods, fires, and conflicts that can disrupt treatment sessions and jeopardize patient and healthcare worker safety. This scoping review maps international evidence on the preparation, evacuation and relocation of hemodialysis patients during major emergencies. *Methods*: A scoping review was conducted according to the Arksey-O’Malley framework and the PRISMA-ScR (Preferred Reporting Items for Systematic reviews and Meta-Analyses extension for Scoping Reviews) standard. PubMed, CINAHL (Cumulative Index to Nursing and Allied Health Literature), Scopus, and grey sources were searched for studies published between January 2000 and May 2025 in Italian or English. Following screening of titles/abstracts and full texts, 40 studies were included. Data were extracted across six domains: risk management, disconnection, transport, resource allocation, training, and temporary “non-dialytic” strategies. *Results*: Risk management is addressed in 95% of the studies, with HIRA (Hazard Identification and Risk Assessment) matrices, mutual aid networks and advance dialysis strategies that have reduced post-event hospitalizations. Only 17.5% of the studies describe protocols for disconnecting from extracorporeal circulation; 72.5% focus on evacuation and transport. Resource allocation appears in 72.5% of the articles, and training in 60%. Restrictive dietary strategies, potassium-binding resins, and diuretics in patients with residual diuresis serve as a temporary bridge when dialysis is suspended. *Conclusions*: Dialysis resilience stems from the integration of hospital governance, infrastructure redundancy, interprofessional expertise, and active patient participation. Multicenter trials, uniform outcome indicators, and standardized national protocols are needed to transform the recommendations into binding standards.
**Keywords:** maxi-emergencies; evacuation; hemodialysis

**SC42. Care of the patient in Double Filtration Therapeutic Apheresis: a current challenge for the Dialysis nurse. The ASL CN1 experience.**
Alfonso Pecoraro, Stefania Zeri, Erika Naso, Federica Tomatis, Rossana Carrera, Stefano Dubois Chiabrando, Claudia Mattarozzi and Marita Marengo
S.C. Nephrology and Dialysis, ASLCN1, Cuneo, Italy; alfo.8723@gmail.com
**Abstract**
*Background/Objectives:* To describe the experience of the Nephrology and Dialysis Unit of ASL CN1 (Cuneo, Italy) with Double Filtration Plasmapheresis (DFPP). The focus is on the dialysis nurse’s role in managing this complex technique applied to immune-mediated neurological pathologies that cause a rapid loss of patient autonomy. *Methods*: We analyzed our experience with DFPP treatments: since 2022, 14 patients (9 M, 5 W; mean age 56.3 years) with Guillain-Barré Syndrome, Myasthenia Gravis, and other immune-mediated neuropathies were treated. Management follows a multidisciplinary approach: the neurologist provides the clinical indication, the nephrologist manages vascular access and prescription, and the dialysis nurse is responsible for the technical and care setting. *Results*: A total of 64 treatments were performed. Nursing care proved central to process safety through: management and monitoring of vascular access; careful monitoring of Transmembrane Pressure (TMP) as an indicator of membrane fouling; early assessment of treatment-related clinical issues (e.g., hemolysis, hypotension); and psychological support, alarm management, and continuity of care in collaboration with Neurology and Intensive Care Units. Conclusions: DFPP is a complex procedure requiring high technical and relational skills. The specialized dialysis nurse, integrated into the multidisciplinary team, is essential to guarantee therapeutic efficacy and high-quality holistic care, contributing significantly to treatment success.
**Keywords:** therapeutic plasmapheresis; nurse; nephrology

**SC43. The role of nursing ultrasound in the management of DVA (Difficult Vascular Access) in the Dialysis Area**
Lemma Ruggiero ^1,^*, Petruzzelli Giuseppe ^1^, Petruzzelli Grazia ^2^, Biga Maria Luisa ^3^, Pasquale Vincenzo Pietro ^1^, Quacquarelli Giuseppe ^1^, Pischetola Raffaella ^1^ and Piccolo Tiziana ^4^

1 Dialysis Center, Barletta Hospital, ASL BT, Barletta, Italy
2 NephroCare Dialysis Center, Minervino Murge, Italy
3 U.O.C. Nephrology and Dialysis, ASL BT, Andria, Italy
4 U.O.C. Nephrology and Dialysis, Barletta Hospital, ASL BT, Barletta, Italy

* Correspondence: ruggiero.lemma1987@gmail.com
**Abstract**
*Background/Objectives:* Preserving vascular accesses is essential in hemodialysis, but when an access is already compromised, the priority shifts from prevention to management of ongoing dysfunction. In this setting, nurse-performed ultrasound may be a valuable clinical tool. *Case presentation*: We report the case of R.F., a 45-year-old patient with end-stage chronic kidney disease who has been on hemodialysis for approximately 18 months through a native arteriovenous fistula (AVF) created 24 months ago. During a dialysis session, AVF malfunction was detected, with absence of thrill, making continuation of treatment impossible. The patient was referred to the Emergency Department of Barletta Hospital, where placement of a temporary central venous catheter (CVC) was initially indicated. Before CVC placement, bedside ultrasound was performed by the on-duty Nephrology nurse ultrasonographer. The examination included vascular access mapping, identification of endoluminal hypoechoic areas, and blood flow measurement, showing severely reduced flow (Qa = 200 mL/min). Based on the ultrasound findings, two cannulation sites were selected: an arterial access distal to the lesions, and a venous access proximal to them, in order to optimize residual blood flow. The following day, the patient underwent successful percutaneous transluminal angioplasty (PTA). In the meantime, effective hemodialysis was completed for 4 h using the residual AVF flow, achieving adequate depuration and fluid balance without temporary CVC placement. *Conclusions:* This case highlights the clinical value of nurse-performed ultrasound in identifying safe cannulation sites, supporting dialysis delivery, and potentially avoiding invasive procedures such as temporary CVC insertion.
**Keywords:** arteriovenous fistula; point-of-care ultrasound; hemodialysis

**SC44. The choice to donate: heroes of donation are born at school**
Virginia Lacanale and Maria Rosa Barattucci
Hemodialysis Department, ASL 02 Lanciano-Vasto-Chieti, Via San Camillo De Lellis, Vasto, Chieti, Italy; virginialacanale@asl2abruzzo.it
**Abstract**
*Background/Objectives:* Organ and tissue donation is a public health priority and an act of solidarity. Despite national awareness campaigns, misinformation and doubts persist among young people. Schools are a strategic setting to promote informed choices, with nurses playing a key educational role. The objective was to promote a culture of donation among 1700 secondary school students through a multidisciplinary intervention, highlighting the nurse’s role in fostering awareness and social responsibility. *Methods*: A total of 1700 students participated in interactive sessions led by a multidisciplinary team. Nurses explained the transplant waiting list, dialysis experience, and post-transplant follow-up. Patient video testimonials were shown, and a creative contest (“Heroes of donation are born at school”) was organized. An anonymous feedback questionnaire was administered. *Results*: 90% of students supported organ donation. Many had prior exposure through family, school, or social media. The preferred way to express consent was through ID card renewal. Student work showed strong awareness and empathy. *Conclusions*: A multidisciplinary and participatory approach was well accepted and was associated with high reported support for organ donation. Nurses are key figures in promoting donation culture, combining clinical expertise, education, and humanized care.
**Keywords:** organ donation and transplantation; adolescent health promotion; multidisciplinary educational intervention

**SC45. MUST technique (Multiple Single Cannulation Technique): a new adventure for our dialysis center**
Lucia Principe
U.O.C. Nephrology and Dialysis, San Donato Hospital, Azienda USL Toscana Sud Est, Arezzo, Italy; lucia.principe@uslsudest.toscana.it
**Abstract**
*Background/Objectives:* This study aimed to identify a reliable puncture technique that is practical for operators and less painful for patients. The Multiple Single Cannulation Technique (MUST) is based on establishing multiple predefined puncture sites and ensuring appropriate rotation and needle insertion to reduce complications in arteriovenous fistulas (AVFs). *Methods*: The MUST technique was applied to newly created AVFs and to patients with low tolerance for venipuncture pain, with the aims of preserving aesthetic appearance, promoting homogeneous AVF development, and monitoring pre-existing aneurysms. The method involved patient selection, ultrasound evaluation of AVF anatomy, identification of puncture sites (three for the arterial needle and three for the venous/re-entry needle), dermographic marking of the selected sites for approximately 10 sessions, and sharing images with the nursing team. *Results*/*Conclusions*: In this experience, the technique supported successful cannulation of difficult AVFs, pain control, preservation of the aesthetic appearance of the AVF arm, and stability of pre-existing aneurysms. The MUST technique was reported as satisfactory for both operators and patients.
**Keywords:** Innovative; repeatable; satisfying

**SC46. Palliative Hemodialysis in the Frail Patient: Experience in Early Identification and Shared Care**
Maria Gabriella, Maria Cristina Gambirasio and Gianluca Bergna
U.O. Dialysis, ASST Nord Milano, Bassini Hospital, Cinisello Balsamo (Milan), Italy; luca.bergna@asst-nordmilano.it
**Abstract**
*Background/Objectives:* In frail patients, particularly elderly individuals with multiple comorbidities and limited life expectancy, hemodialysis-related complications may lead to disability, recurrent exacerbations, and progressive clinical decline with reduced quality of life. An integrated model combining dialysis and palliative care is increasingly recommended to address these needs. This study aims to describe an approach focused on early identification and shared decision-making. *Methods*: Participation in a Lombardy-based multidisciplinary Working Group on Palliative and Simultaneous Care in Frail Elderly Nephropathic Patients (FAN) led to the development of a nursing assessment tool for early identification of clinical deterioration. Continuous observation period involving several patients, combined with shared management involving a palliative care specialist and a psychologist, enabled the evaluation of treatment proportionality and the planning of individualized care pathways. *Results*: Among patients, the model facilitated shared care planning with patients and families up to the terminal phase. Multidisciplinary collaboration improved team cohesion, reduced emotional burden among healthcare professionals, and enhanced the humanization of care and family involvement. *Conclusions*: Simultaneous dialysis, integrated into a structured multidisciplinary pathway supported by palliative care, represents an ethical and patient-centered approach in advanced disease stages. This experience highlights the importance of innovation not only in technology but also in care processes, promoting humanization, professional collaboration, and reflective practice.
**Keywords:** palliative care; hemodialysis; frailty; shared decision-making; humanization

**SC47. Underreporting of CKD-Associated Pruritus in Hemodialysis Patients: A Qualitative Study and Implications for Proactive Nursing Assessment**
Silvia Cappelletti
ASST Lariana, Hemodialysis Unit, Como, Italy; silviacappelletti74@outlook.com
**Abstract**
*Background/Objectives:* Chronic Kidney Disease-Associated Pruritus (CKD-aP) is a common and burdensome complication in hemodialysis patients, affecting sleep, emotional well-being, and quality of life, with prevalence rates reaching up to 70% in international registries. Despite its clinical relevance, CKD-aP remains frequently underreported and insufficiently explored in routine nursing assessment. This study aimed to explore patients’ lived experiences of CKD-aP and identify mechanisms contributing to symptom underreporting. *Methods*: A qualitative study with a phenomenological-interpretative approach was conducted. Ten semi-structured interviews were performed with adult hemodialysis patients undergoing treatment for at least 12 months and presenting documented CKD-aP. Interviews were audio-recorded, transcribed verbatim, and analyzed using inductive thematic analysis according to Braun and Clarke. Progressive theme identification was performed until conceptual saturation was achieved. The observational non-interventional study was approved by the local bioethics office, and written informed consent was obtained from all participants. *Results*: Four main thematic areas emerged: (1) sleep impairment and persistent fatigue; (2) emotional distress and loss of control; (3) symptom normalization and minimization; and (4) the nurse’s role as a facilitator for symptom disclosure. Patients reported pruritus more frequently when specifically asked through targeted nursing questions, confirming significant spontaneous underreporting. *Conclusions*: CKD-aP represents a clinically relevant but frequently invisible symptom in dialysis care. Systematic integration of proactive screening questions during dialysis sessions may improve early identification, patient-centered care, and symptom management.
**Keywords:** CKD-associated pruritus; hemodialysis; nursing assessment; qualitative research; symptom underreporting

**SC48. Nursing Protocol for Skin Barrier Restoration in CKD-Associated Pruritus: A Prospective Pilot Study in Hemodialysis Patients**
Silvia Cappelletti
ASST Lariana, Hemodialysis Unit, Como, Italy; silviacappelletti74@outlook.com
**Abstract**
*Background/Objectives:* Chronic Kidney Disease-Associated Pruritus (CKD-aP) is a frequent and burdensome complication in hemodialysis patients, significantly affecting sleep quality, emotional well-being, and quality of life. Skin xerosis represents a relevant and potentially modifiable contributing factor. This pilot study aimed to evaluate the feasibility, acceptability, and preliminary efficacy signal of a nursing protocol focused on skin barrier restoration in patients with CKD-aP. *Methods*: A single-center prospective pilot study was conducted in five chronic hemodialysis patients presenting CKD-aP and clinically evident xerosis. The 12-week nursing protocol included daily gentle cleansing with physiological pH detergents, twice-daily application of barrier emollients containing ceramides, urea, and glycerol, and therapeutic education on skin care. The primary endpoint was change in Worst Itch Numerical Rating Scale (WI-NRS). Secondary endpoints included xerosis severity (Overall Dry Skin Score, ODS), sleep disturbance (SADS), adherence, tolerability, and patient-reported experience. Descriptive longitudinal analysis and inductive thematic qualitative analysis were performed. Results: Clinically relevant improvement in pruritus was observed, particularly in patients with mild-to-moderate CKD-aP, together with consistent reduction in xerosis severity. Adherence to the protocol was high, with no adverse events reported. Qualitative findings highlighted improved symptom controllability, reduced nocturnal discomfort, and better perception of skin condition. Patients with severe pruritus showed partial but less sustained benefit. *Conclusions:* The proposed nursing protocol appeared feasible, safe, and associated with preliminary symptomatic improvement in CKD-aP. Skin barrier restoration may represent a simple and implementable non-pharmacological strategy within multimodal symptom management in hemodialysis care. Larger controlled studies are needed to confirm these findings.
**Keywords:** CKD-associated pruritus; hemodialysis; skin barrier; nursing protocol; xerosis

**SC49. Eco-Controlling: A Structured System for Green Management of Dialysis Centers and Reduction of Environmental Impact**
Alessandro Pizzo, Giuseppe Santarcangelo, Gessica De Pascale
NephroCare Italia, Napoli, Italia. Alessandro.Pizzo@Freseniusmedicalcare.com
**Abstract**
*Background/Objectives:* Dialysis therapy requires substantial water and energy consumption and generates considerable amounts of hazardous healthcare waste, contributing significantly to the environmental footprint of nephrology care. National studies have highlighted the need for the implementation of “Green Dialysis” practices to mitigate these impacts, in line with current European sustainability recommendations. To address this challenge, Fresenius Medical Care developed a digital dashboard system for monitoring key environmental performance indicators across its Italian dialysis network. To implement an Eco-Controlling system through digital dashboards for monitoring electricity consumption (kWh/treatment), water consumption (liters/treatment), hazardous healthcare waste (BCW, kg/treatment), and number of treatments, with the aim of optimizing green management strategies and reducing environmental impact. *Methods*: The Fresenius Eco-Controlling Dashboard integrates monthly data from management information systems and technical plant infrastructures. The platform provides real-time visualization of key indicators, including electricity consumption (40.8 kWh/treatment in December 2025), water consumption (413 liters/treatment), and BCW generation (0.87 kg/treatment), as well as trend analyses, inter-center benchmarking, and standard deviation assessments. Data are reviewed locally by multidisciplinary teams and centrally validated to ensure consistency and reliability. *Results*: A total of 341,207 hemodialysis treatments were monitored across the dialysis network during 2025. Annual mean values were 41.5 kWh/treatment for electricity consumption, decreasing from 43.1 kWh/treatment in January to 40.8 kWh/treatment in December; 415 liters/treatment for water consumption; and 0.85 kg/treatment for BCW generation. Downward trends were observed across environmental indicators, reflecting the positive impact of technical and organizational optimization initiatives implemented within the network. *Conclusions*: The Eco-Controlling Dashboard proved to be an effective and reproducible tool for routine environmental monitoring in dialysis care. Practical applications include monthly environmental audits, benchmarking activities, and targeted staff training programs. For healthcare organizations, the system supports data-driven decision-making for sustainability investments, regulatory compliance, resource optimization, and potential reductions in resource consumption of approximately 5–10%, contributing to a favorable environmental return on investment. This model represents a scalable approach to promoting sustainability within nephrology services.
**Keywords:** Green Dialysis; Sustainability; Environmental Impact; Eco-Controlling; Hemodialysis; Healthcare Waste; Water Consumption; Energy Efficiency

## 3. Concluding Remarks

The 44th National Congress of SIAN highlighted the continued evolution of nephrology nursing toward an increasingly evidence-based, technologically advanced, patient-centred, and multidisciplinary model of care [1,2,3,4,5]. The scientific contributions presented during the pre-congress session and the main Congress reflected the broad range of competencies now required in nephrology practice, encompassing dialysis care, vascular access management, kidney transplantation, symptom assessment, palliative and supportive care, nutrition, patient safety, digital health, sustainability, education, and professional development [6,7,8,9,10]. A recurring theme throughout the meeting was the importance of integrating scientific evidence with clinical expertise and person-centred care. Technological innovation, including ultrasound applications, telemonitoring, digital tools, and artificial intelligence, offers important opportunities to improve assessment, monitoring, clinical decision-making, and continuity of care. At the same time, the contributions emphasized that technological advancement should complement rather than replace the relational, educational, and human dimensions of nursing care. The pre-congress session further emphasized the importance of strengthening research competencies from the earliest stages of nursing education. Providing students and early-career nurses with opportunities to engage in scientific activities, present research, interact with experienced clinicians and researchers, and participate in professional networks may contribute to the development of a stronger and more sustainable research culture within nephrology nursing [11,12,13,14,15,16,17,18,19,20].

Several contributions also highlighted areas in which further research is required, including the standardization of assessment tools and nursing protocols, evaluation of innovative care models, development of digital and home-based interventions, and generation of stronger evidence regarding nursing-sensitive outcomes. These priorities underline the need for continued collaboration among clinical professionals, universities, healthcare organizations, patients, and scientific societies. Overall, the Congress demonstrated the value of scientific exchange as a driver of professional growth and improvement in kidney care. By bringing together education, research, clinical practice, technological innovation, and professional experience, SIAN can continue to support the development of nephrology nursing and contribute to safer, more effective, sustainable, and person-centred care for people living with kidney disease [21,22,23,24,25,26,27,28,29,30].

## Data Availability

No new research data were generated or analyzed for the preparation of these conference proceedings. The publication includes abstracts and contributions submitted by the authors for presentation at the 44th National Congress of SIAN. Any data referenced within individual abstracts remain the responsibility of the respective authors.

## Supplementary Materials

The following supporting information can be downloaded at: https://www.mdpi.com/article/10.3390/healthcare14203445/s1, File S1: Complete scientific program of the 44th National Congress (PCS and SC).

## Appendix A

SIAN Board of Directors (contributed as authors of the manuscript):

1. Domenica Gazineo: Italian Society of Nephrology Nurses (SIAN) Board of Directors, Via Capotesta 1/30, 07026 Olbia, Italy; Governo Clinico e Qualità, IRCCS Azienda, Ospedaliero-Universitaria di Bologna, Bologna, Italy; domenica.gazineo3@unibo.it;
2. Addolorata Palmisano: Italian Society of Nephrology Nurses (SIAN) Board of Directors, Via Capotesta 1/30, 07026 Olbia, Italy; dorianapalmisano77@gmail.com;
3. Alessandro Pizzo: Italian Society of Nephrology Nurses (SIAN) Board of Directors, Via Capotesta 1/30, 07026 Olbia, Italy; pizzoalexxandro@gmail.com;
4. Silvia Cappelletti: Italian Society of Nephrology Nurses (SIAN) Board of Directors, Via Capotesta 1/30, 07026 Olbia, Italy; silviacappelletti74@outlook.com;
5. Angela Greco: Italian Society of Nephrology Nurses (SIAN) Board of Directors, Via Capotesta 1/30, 07026 Olbia, Italy; angelagreco1967@gmail.com;
6. Francesco Barci: Italian Society of Nephrology Nurses (SIAN) Board of Directors, Via Capotesta 1/30, 07026 Olbia, Italy; francescobarci81@gmail.com.

* Email: direttivo@sianitalia.it
