# Supplementary material for: 44th National Congress of the Italian Society of Nephrology Nurses (SIAN)
Source: Healthcare (Basel). 2026 Oct 14;14(20):3445. doi: 10.3390/healthcare14203445 (PMC13704731; doi:10.3390/healthcare14203445)
Supplement: Supplementary file 1 [file healthcare-14-03445-s001.zip › healthcare-4602412-supplementary.pdf]

Società Infermieri Area Nefrologica
SIAN Italia

4-5-6 Maggio 2026
Zanhotel Europa - Bologna

Prevenzione, Cura, Innovazione

Nuove prospettive per
l'Infermieristica Nefrologica

44° CONGRESSO NAZIONALE SIAN

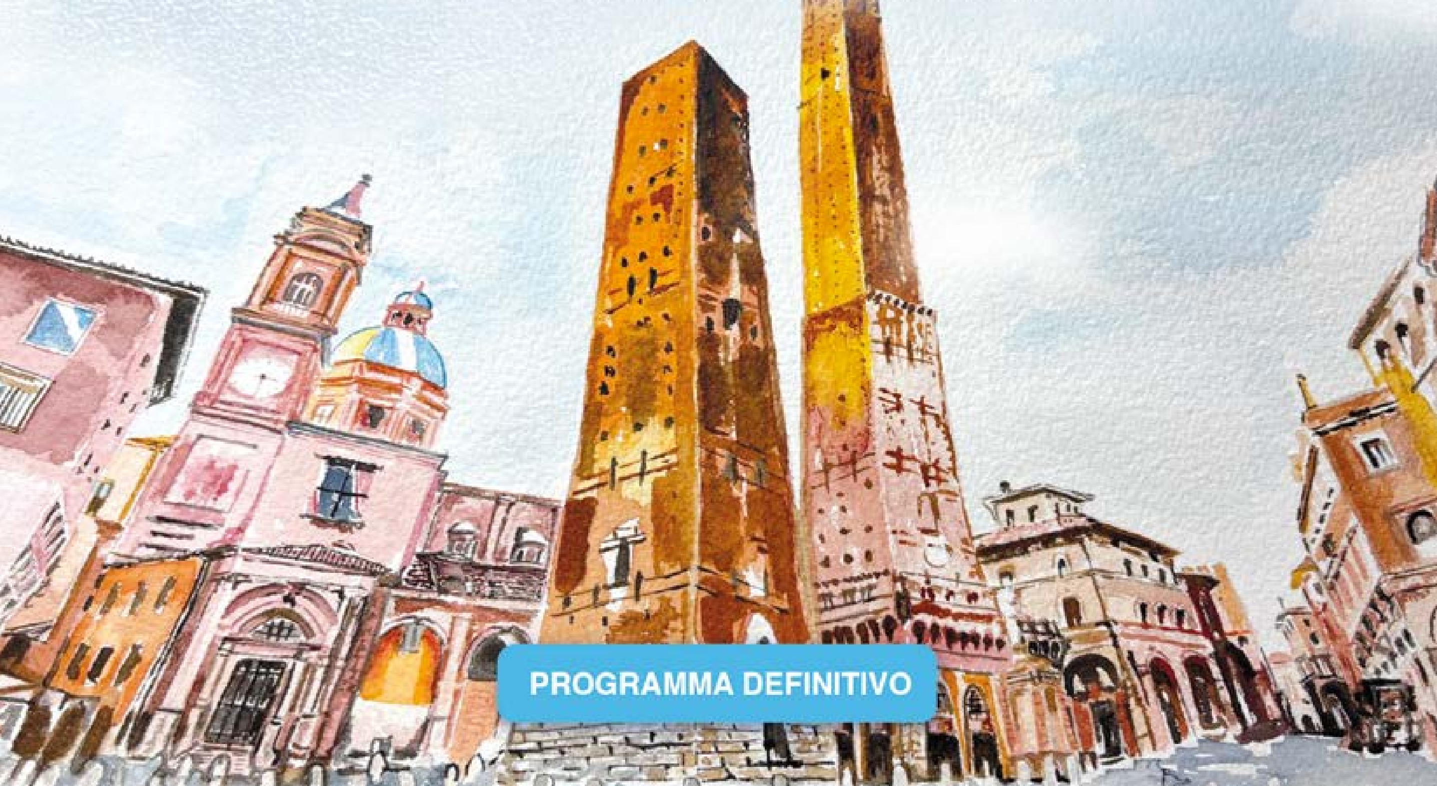

PROGRAMMA DEFINITIVO

44° CONGRESSO NAZIONALE SIAN

Prevenzione, Cura, Innovazione

Nuove prospettive per
l'Infermieristica Nefrologica

4-5-6 Maggio 2026

Con il Patrocinio di

Associazione Nazionale Emodializzati
Dialisi e Trapianto - APS
Medaglia d'Oro al Merito della Sanità Pubblica

www.sianitalia.it

**Presidente del Congresso**	**Stefano Mancin**
**Responsabile Comitato Scientifico**	**Domenica Gazineo**
**Comitato Scientifico Congressuale**	**Francesco Barci**<br>**Silvia Cappelletti**<br>**Lea Godino**<br>**Angela Greco**<br>**Addolorata Palmisano**<br>**Francesco Pastore**<br>**Alessandro Pizzo**<br>**Marco Sguanci**

CONSIGLIO DIRETTIVO SIAN

**Presidente**	**Stefano Mancin**
**Vice Presidente**	**Domenica Gazineo**
**Segretaria**	**Silvia Cappelletti**
**Tesoriere**	**Francesco Barci**
**Componenti consiglio direttivo**	**Angela Greco**<br>*Coordinatore Referente Operativo Regionale*<br>**Addolorata Palmisano**<br>*Coordinatore Referente Gruppi di Interesse*<br>**Alessandro Pizzo**<br>*Componente Consiglio Direttivo*

Cari colleghi,

è con grande entusiasmo che vi diamo il benvenuto al Congresso Nazionale SIAN 2026, un appuntamento ormai consolidato, ma ogni anno rinnovato nei contenuti e nello spirito.

Il titolo e il filo conduttore di questa edizione, centrato su responsabilità professionale, innovazione e integrazione, rappresenta la naturale evoluzione del percorso intrapreso dalla nostra comunità scientifica negli ultimi anni.

La nefrologia moderna vive oggi una fase di profonda trasformazione: nuove tecnologie, terapie integrate, percorsi territoriali e una crescente attenzione alla qualità della vita dei pazienti ridefiniscono il ruolo dell'infermiere, sempre più competente, responsabile e parte attiva nella gestione della cronicità.

Ricerca, formazione e narrazione diventano strumenti per tradurre la conoscenza in cura, per costruire relazioni terapeutiche più consapevoli e umanizzate, capaci di affiancare la dimensione tecnica a quella empatica e comunicativa.

Il Congresso di quest'anno vuole essere un laboratorio di confronto, dove teoria e pratica si incontrano: sessioni plenarie, tavole rotonde, laboratori simulativi e momenti di scambio con le aziende offriranno a tutti noi la possibilità di approfondire le diverse anime dell'assistenza nefrologica e di sperimentare nuove forme di collaborazione interdisciplinare.

A nome del Direttivo SIAN, vi auguriamo buon lavoro e buona partecipazione.

Il Presidente
Stefano Mancin

1981-2026 CONGRESSI NAZIONALI-REGIONALI EDTNA/ERCA - SIAN *(SOCIETÀ INFERMIERI DI AREA NEFROLOGICA)*

1° 1981 EDTNA - Salsomaggiore
2° 1982 EDTNA - Verona
3° 1983 EDTNA – Francavilla – Chieti
1984 - 1° Conferenza Internazionale EDTNA-EDTA Firenze
4° 1985 EDTNA Lecce
5° 1986 EDTNA/ERCA - Bologna
6° 1987 EDTNA/ERCA - Torino
7° 1988 EDTNA/ERCA - Bergamo
8° 1989 EDTNA/ERCA - Cremona
9° 1990 EDTNA/ERCA - Alba
10° 1991 EDTNA/ERCA - Arezzo
11° 1992 EDTNA/ERCA Filiale italiana
1992 1° Regionale Sardegna - Oristano
12° 1993 EDTNA/ERCA Filiale italiana - Brescia
13° 1994 EDTNA/ERCA Filiale italiana e 1° Meeting Dialisi Peritoneale-Montesilvano
14° 1995 EDTNA/ERCA Filiale italiana - Verona
15° 1996 EDTNA/ERCA Filiale italiana e 2° Meeting Dialisi Peritoneale, Baveno
16° 1997 EDTNA/ERCA Filiale italiana - Milano
17° 1998 EDTNA/ERCA Filiale italiana - Bari
18° 1999 EDTNA/ERCA Filiale italiana - Firenze
19° 2001 EDTNA/ERCA Filiale italiana e 4° Meeting Dialisi Peritoneale - Rimini
20° 2002 EDTNA/ERCA Filiale italiana - Bologna
21° 2003 EDTNA/ERCA Filiale italiana - Viterbo
22° 2004 EDTNA/ERCA Filiale italiana - Lecce
23° 2005 EDTNA/ERCA Filiale italiana - Bologna
24° 2006 EDTNA/ERCA Filiale italiana - Roma
25° 2007 Internazionale EDTNA/ERCA - Firenze
26° 2008 EDTNA/ERCA Filiale italiana - Riccione
27° 2009 EDTNA/ERCA Filiale italiana - Riccione
28° 2010 EDTNA/ERCA Filiale italiana - Riccione
29° 2011 EDTNA/ERCA Filiale italiana - Bellaria
30° 2012 EDTNA/ERCA Filiale italiana - Milano Marittima
31° 2013 EDTNA/ERCA Filiale italiana - San Benedetto Del Tronto
32° 2014 EDTNA/ERCA Filiale italiana - San Benedetto Del Tronto
33° 2015 EDTNA/ERCA Filiale italiana - Bari
2015 2° Regionale Sardegna - Oristano
34° 2016 EDTNA/ERCA Filiale italiana
2016 EDTNA/ERCA Filiale italiana - Regionale Piemonte, Torino
35° 2017 EDTNA/ERCA Filiale italiana - Riccione
2017 Interregionale (Friuli-Veneto-Trentino), Padova
36° 2018 EDTNA/ERCA Filiale italiana - Riccione
2018 Interregionale (Toscana-Emilia Romagna-Marche), Firenze
37° 2019 SIAN RICCIONE
2019 SIAN (Interregionale Basilicata-Puglia-Campania) Matera
38° 2020 SIAN - Pacchetti Formativi Webinar (Pandemia)
39° 2021 SIAN - Bologna
40° 2022 SIAN - Rimini
2022 SIAN Interregionale (Campania-Calabria), Castellammare di Stabia
41° 2023 SIAN - Riccione
2023 SIAN 1° Regionale Calabria - Rende
42° 2024 SIAN - Riccione
2024 SIAN 2° Regionale Calabria -Rende
2024 SIAN Regionale Veneto- Padova
43° 2025 SIAN - Rimini
2025 Regionale Lombardia - Como
2025 SIAN 3° Regionale Calabria - Rende
44° 2026 SIAN - Bologna

PRESIDENTI

**Aurelia Castagnoli**	*1981-1991 - 1° Key Member per EDTNA/ERCA*
**Sandro Geatti**	*1991- 2008 - da Key member per EDTNA/ERCA a Presidente Filiale Italiana*
**Marisa Pegoraro**	*2008-2019 - Presidente Filiale Italiana EDTNA/ERCA*
**Cinzia Fabbri**	*2019-2025 - 1° Presidente SIAN*
**Stefano Mancin**	*2025-2028 - 2° Presidente SIAN*

FACULTY

Acquistapace Erika
Inf. di Pronto Soccorso, ASST Lecco Ospedale A. Manzoni di Lecco

Barci Francesco
Inf. U.O.C. Nefrologia e Dialisi, Az. Osp. di Cosenza

Barile Elena
Inf. Emodialisi, Osp. don Tonino Bello di Molfetta

Baruzzo Enrico
Inf. U.O.C. Nefrologia e Dialisi, Az. Osp. Università di Padova

Benetti Lucia
Scrittrice e Formatrice

Bua Antonia
Inf. S.C .Nefrologia e Dialisi, Centro Dialisi San Camillo, ASL n. 1 di Sassari

Buccione Emanuele
Inf. Terapia Intensiva, ASL di Pescara

Campanella Francesca
Inf. Terapia Intensiva, Humanitas Mater Domini di Castellanza (VA)

Cangelosi Giovanni
Inf. Coordinatore, ASUR Marche Area Vasta 4 di Fermo

Canzi Mara
Componente Collegio dei Probiviri

Cappelletti Silvia
Inf. Emodialisi e Dilisi Peritoneale ASST Lariana Osp. di Como

Corti Silvia
Inf. Emodialisi, AUSL Toscana Sud-Est di Arezzo

Dalprà Anna
Gruppo Infermieri Dialisi Peritoneale SIAN GIPD

De Simone Immacolata
Inf. Nefrologia e Dialisi, IRCCS Ca' Granda Osp. Maggiore Policlinico di Milano

Esposto Rossella
Inf. Emodialisi, ASL Cn2 Osp. Michele e Pietro Ferrero di Verduno (CN)

Fabbri Cinzia
Past President SIAN

Fabbrini Paolo
Direttore SC Nefrologia e Dialisi, ASST NORD Milano Osp. Bassini di Cinisello Balsamo (MI)

Faraone Vincenzo
Incaricato di funzione organizzativa T.A.V. Team Accessi Vascolari A.O.R.N. A Cardarelli Napoli

Fenoglio Giuseppe
Inf. Emodialisi, ASL Cn2 Osp. Michele e Pietro Ferrero di Verduno (CN)

Ferrara Gaetano
Dip. Biomedicina e Prevenzione, Università di Roma "Tor Vergata"; Inf. Nefrologia e Dialisi, AUSL Modena Osp. Di Carpi (MO)

Ferraris Alice
Inf. Coordinatore Emodialisi, A.O.U. San Luigi Gonzaga di Orbassano (TO)

Gambirasio Maria Cristina
Inf. Coordinatore U.O. Dialisi, ASST Nord Milano Osp. di Cinisello Balsamo (MI)

Gazineo Domenica
IRCCS Az. Ospedaliero-Università di Bologna

Gesualdo Loreto
Direttore Medico Nefrologia e Dialisi, Università degli Studi di Bari

Giordano Vincenza
Inf. Dip. Sanità Pubblica, A.U.O. Federico II di Napoli

Godino Lea
U.O.C. Genetica Medica; IRCCS Az. Ospedaliero-Università di Bologna

Grasso Giovanni
Inf. Emodialisi, ASST Nord Milano Osp. di Cinisello Balsamo (MI)

Gravante Francesco
Inf. Emodialisi, ASL Caserta, P.O. San Giuseppe Moscati di Aversa (CE)

Greco Angela
Inf. Coordinatore U.O.C. Nefrologia, Dialisi e Trapianto, Az. Osp. di Cosenza

Guarascio Angelo
Inf. Nefrologia e Dialisi, Az. Osp. SS Annunziata di Cosenza

Lentini Paolo
Direttore U.O.C. Nefrologia e Dialisi, Az. ULSS7 Pedemontana Osp. San Bassiano di Bassano del Grappa (VI)

Lenzi Donatella
Inf. Nefrologia e Dialisi, Az. ULSS7 Pedemontana Osp. San Bassiano di Bassano del Grappa (VI)

Limonti Francesco
Inf. Blocco Operatorio, A.O.U. di Ferrara

Lombardi Marco
Rappresentante GdP Green Nephrology e contrasto cambiamento climatico SIN Società Italiana di Nefrologia

Longo Walter
Inf. Dialisi Peritoneale, Az. Ospedaliero-Universitaria IRCCS di Bologna

Longobardi Antonio
Inf. Coordinatore Donatori di sangue, Ambulatorio Aferesi Terapeutica SC 580 Trapianti Lombardia-NITp, IRCCS Ca' Granda Osp. Maggiore Policlinico di Milano

Lovison Luca
Inf. Coordinatore Nefrologia e Dialisi AULSS7 Pedemontana Bassano del Grappa (VI)

Maisto Francesco
Inf. Dialisi, ASST OVEST Milanese, Legnano (MI)

Mancin Stefano
Inf. e Biologo Nutrizionista IRCCS Istituto Clinico Humanitas, Rozzano (MI); Presidente SIAN

Marconi Lorenzo
SSD Gestione Clinica delle Emergenze Epidemiologiche, IRCCS Az. Ospedaliero-Università di Bologna

Matteucci Sofia
Inf. IRCCS Istituto Clinico Humanitas, Rozzano (MI)

Mechini Cristina
Inf. U.O.C. Nefrologia, Dialisi e Trapianto, Az. Ospedaliero Universitaria Senese

Meo Stefania
Inf. Nefrologia, ASST Grande Ospedale Metropolitano Niguarda, Milano

Mettifogo Mariangela
Inf. Emodialisi, Ospedale San Bortolo di Vicenza

Morales Palomares Sara
Inf. Nefrologia, Dialisi e Trapianto, Az. Osp. di Cosenza

Palmisano Addolorata
Inf. Nefrologia e Dialisi, AULSS 6 Euganea di Monselice (PD)

Pastore Francesco
Ricercatore e Inf. di Ricerca, Università di Bari

Pegoraro Marisa
Inf. Senior Nefrologia, in libera professione

Pirreco Angelo
U.O.C. Nefrologia e Dialisi, A.O.U. Policlinico G. Rodolico-San Marco di Catania Dip. Biomedicina e Prevenzione, Università di Roma Tor Vergata

Pizzo Alessandro
Quality e Nursing manager NephroCare Italia

Ricci Zaccaria
Direttore S.O.C. Anestesia e Rianimazione, IRCCS Az. Ospedaliero-Universitaria Meyer; Professore Associato, Dip. di Scienza della Salute, Università degli Studi di Firenze

Sandri Elena
Inf. Coordinatore, Facoltà di Medicina e Scienze della Salute, Università Cattolica di Valencia "San Vicente Mártir"

Sguanci Marco
Inf. Dipartimento di Medicina e Chirurgia, Unità di Ricerca di Scienze Infermieristiche, Università di Roma, Campus Bio-Medico, Bologna

Siclari Teresa
Segretario Regionale e Tesoriere ANED Onlus Lombardia

Soloperto Silvia
Inf. Coordinatore S.C. Nefrologia, Dialisi e Trapianto, IRCCS Ca' Granda Osp. Maggiore Policlinico di Milano

Vrenna Giada
Inf. ASST Grande Ospedale Metropolitano Niguarda, Milano

Zaza Gianluigi
Professore Ordinario Nefrologia, Università Sapienza di Roma; U.O.C. di Nefrologia, Dialisi e Trapianto, Ospedale Umberto I di Roma

LUNEDÌ 4 MAGGIO 2026

Sala Plenaria — **PERCORSO NON ACCREDITATO ECM**

13.30 Registrazione partecipanti

Giornata di apertura dedicata all'evoluzione del ruolo infermieristico in nefrologia, alle nuove competenze, alla promozione della salute e alle prospettive di ricerca. L'obiettivo è valorizzare la responsabilità professionale, la comunicazione e l'integrazione multiprofessionale nei percorsi di cura.

14.00 – 14.20 Saluti introduttivi e Apertura dei lavori

14.20 **Lettura Magistrale**
Le sfide per il SSN e la nefrologia da oggi ai prossimi 20 anni
Loreto Gesualdo

14.40 – 15.30 **Tavola Rotonda**
POSITION PAPER SIAN COMPETENZE INFERMIERISTICHE IN NEFROLOGIA E DIALISI

partecipano:
- **SIAN** *Società Infermieri Area Nefrologica*
- **FNOPI** *Federazione Nazionale Ordini Professioni Infermieristiche*
- **SIN** *Società Italiana Nefrologia*
- **ANED** *Associazione Nazionale Emodializzati*

15.30 – 16.15 I Sessione
PREVENZIONE E PRESA IN CARICO PROATTIVA NEL PAZIENTE NEFROPATICO: MODELLI ORGANIZZATIVI E IMPATTO DI SANITÀ PUBBLICA
Moderatori: **Domenica Gazineo, Lea Godino**

15.30 Strategie di prevenzione e presa in carico nella malattia renale cronica: integrazione dei percorsi in dialisi e trapianto
Lorenzo Marconi

15.45 Il ruolo dell'infermiere nella presa in carico e nella promozione della salute del paziente nefropatico
Giovanni Cangelosi

16.00 Dibattito

16:15 – 16:30 ***Coffee break***

16.30 – 17.15

II Sessione

CKD-aP: OLTRE IL SINTOMO. NUOVE PROSPETTIVE ASSISTENZIALI, CLINICHE E MULTIDISCIPLINARI

Moderatore: **Angela Greco**

16.30 Gestione del CKD-aP: interventi infermieristici evidence-based e monitoraggio degli Outcome
Stefano Mancin, Alessandro Pizzo

17.15 – 18.00

III Sessione

IL TRAPIANTO PRE-EMPTIVE DA DONATORE VIVENTE E DA DONATORE DECEDUTO: FOCUS SULL' ATTIVITÀ INFERMIERISTICA NELL'AMBULATORIO MaReA IN ITALIA

Moderatori: **Silvia Soloperto, Gianluigi Zaza**

17.15 Implementazione del trapianto pre-emptive nel Nord Italia: l'esperienza milanese
Antonio Longobardi

17.30 Implementazione del trapianto pre-emptive nel Sud Italia: l'esperienza pugliese
Elena Barile

17.45 Ruolo infermieristico e trapianto pre-emptive: un'indagine conoscitiva a supporto di futuri interventi
Francesco Pastore

18.00 Dibattito

18.15 – 19.00

Assemblea Annuale Ordinaria SOCI SIAN
Assemblea Referenti Operativi Regionali (ROR)

- Relazione consuntiva e programmatica 2025/2026
- Convegni Regionali
- Organigramma Societario
- Sintesi attività regionali e gruppi di lavoro
- Definizione attività future ROR SIAN e gruppi di lavoro
- Collaborazioni Riviste
- Bilancio consuntivo 2025
- Bilancio preventivo 2026

19.00 ***Aperitivo di benvenuto offerto da SIAN***

MARTEDÌ 5 MAGGIO 2026

La giornata del martedì sarà dedicata all'innovazione tecnologica e alla sostenibilità nei percorsi di cura nefrologica, con momenti di confronto tra professionisti, istituzioni e aziende del settore. La mattina si aprirà con una riflessione sulle sfide ambientali e organizzative legate alla pratica dialitica contemporanea, per poi proseguire con i simposi e workshop aziendali, spazio di aggiornamento sulle soluzioni più recenti in ambito di biocompatibilità, ottimizzazione dei trattamenti e riduzione dell'impatto operativo.
Nel pomeriggio, le sessioni parallele e i laboratori simulativi offriranno un approfondimento tecnico e clinico sui principali temi dell'assistenza nefrologica, dalle tecnologie CRRT per i trattamenti per acuti agli accessi vascolari, favorendo un approccio interattivo e interdisciplinare.
La giornata intende così coniugare scienza, innovazione e pratica, promuovendo una cultura infermieristica capace di integrare la competenza tecnica con la responsabilità ambientale e la collaborazione interprofessionale.

Sala Plenaria | **Inizio percorsi formativi ECM n. 1 e 2**

08.30 – 09.45 **Tavola Rotonda**

GREEN DIALYSIS: SOSTENIBILITÀ, SICUREZZA E NUOVI MODELLI ASSISTENZIALI NEL PAZIENTE NEFROPATICO

Conduttori:
Silvia Cappelletti, Marco Lombardi, Addolorata Palmisano, Alessandro Pizzo, Teresa Siclari

08.30 Introduzione

08.40 Micro e nanoplastiche in dialisi: la sostenibilità invisibile
Marco Lombardi

08.55 Cambiamenti climatici e nuovi modelli assistenziali per il paziente cronico, con particolare riferimento al paziente nefropatico
Erika Acquistapace, Giovanni Cangelosi

09.10 Non solo ascolto Q&A dinamico, confronto e definizioni di azioni concrete
Silvia Cappelletti, Marco Lombardi, Addolorata Palmisano, Alessandro Pizzo

09.45 Orientamento verso scelte terapeutiche sempre più consapevoli. Il ruolo della collaborazione tra medico e infermiere
Paolo Fabbrini, Maria Cristina Gambirasio

10.45 – 11.00 ***Coffee break***

MARTEDÌ 5 MAGGIO 2026

11.00
12.30 **Simposi aziendali** **esclusi dal percorso formativo ECM**

11.00
12.00 Gli indicatori sensibili infermieristici in PD e l’innovazione digitale”

Simona Carnoli, Angela Cortigiani, Nicola Libralon, Alessandro Testa, Sabrina Scagnoli, Roberta Marinelli

in collaborazione con Vantive

12.00
12.30 Aferesi terapeutica con piattaforma Omni Plus

Marco Fiorentino

in collaborazione con B.Braun

12.30
13.00 “L’emodialisi leggera”:
continuità terapeutica, sicurezza e miglior qualità di vita nel proprio ambiente

Corrado De Leonardis, Ugo Teatini

in collaborazione con Sapio Life

13.00
14.00 ***Light lunch***

MARTEDÌ 5 MAGGIO 2026

Sala Londra **Proseguimento percorso formativo ECM n. 1**

14.00<br>17.30	**CRRT OGGI: CLINICA, FORMAZIONE E RICERCA PER UNA GESTIONE INFERMIERISTICA**<br>*Moderatori:* **Vincenza Giordano**, **Addolorata Palmisano**, **Zaccaria Ricci**<br>**CRRT NELLA CLINICA: DALLA TEORIA ALLA PRATICA CLINICA**
14.00	Concetti di base e tipologia di trattamento<br>**Zaccaria Ricci**
14.20	Il paziente sottoposto a CRRT: indicazioni cliniche, casi e scenari<br>**Zaccaria Ricci**
14.40	Anticoagulazione in CRRT: eparina vs citrato, criticità e gestione<br>**Mariangela Mettifogo**
15.00	Dibattito
	**LA GESTIONE PRATICA DELLA CRRT**
15.20	Apparecchiature per CRRT: descrizione e fasi operative<br>**Angelo Pirreco**
15.40	Principali allarmi in CRRT e strategie di risoluzione<br>**Emanuele Buccione**
16.00	Criticità operative nella gestione condivisa del trattamento<br>**Mariangela Mettifogo**
16.20	Dibattito
	**CRRT E RICERCA INFERMIERISTICA: EVIDENZE, STRUMENTI E PROSPETTIVE FUTURE**<br>*Moderatori:* **Francesco Gravante**, **Francesco Limonti**, **Zaccaria Ricci**
16.40	Revisione della letteratura sui fattori di rischio associati al fallimento del filtro nel trattamento CRRT<br>**Francesca Campanella**
17.00	Implicazioni future sulla ricerca e la formazione infermieristica<br>**Alice Ferraris**
17.20	Dibattito

MARTEDÌ 5 MAGGIO 2026

17.30 – 18.00 ***Pausa con degustazione alimenti aproteici*** ***in collaborazione con Dr. Schär***

18.00 – 19.00

Sessione di Comunicazioni Orali CRRT e Ricerca infermieristica

***Moderatori:* Francesco Maisto, Marco Sguanci** *(n. 8 comunicazioni da 7 minuti cad.)*

18.00 Gestione delle maxiemergenze con protocolli di evacuazione nei centri di emodialisi: una scoping review
M. Zanon, N. Celon, F. Nalesso *(Padova)*

18.07 Gestione dei rifiuti in emodialisi: misurazione, criticità e interventi migliorativi. Il ruolo chiave dell'infermiere nell'ecosostenibilità del Centro dialisi
R. Pecoraro, G. Di Chiaro, G. Alfano, P. Scannavini, P. Albinelli, S. Sarti, A.R. Villani, G. Donati *(Modena)*

18.14 L'efficacia delle divise antimicrobiche in un centro dialisi: progettazione di uno studio pilota sulla riduzione delle infezioni e la sicurezza dell'operatore
E. Orlandi, V. Di Nardo, L. Vecchi *(Terni)*

18.21 Approccio evidence – based alla gestione del paziente emodializzato con Clostridioides difficile: sviluppo di un care bundle operativo – gestionale
G. Vrenna, C. V. Benedi *(Milano)*

18.28 La "zona grigia" del bilancio idrico: conoscenze infermieristiche sulla perspiratio insensibilis e usabilità dell'app CareBalance-N. Studio osservazionale trasversale
A. Grimaldi[1], V. Di Nuzzo[1], G. Frattolillo[1], D. Di Sivo[2], G. Gavarro[1], I. Chiusolo[3], C. Pozzuoli[1]
([1]Caserta, [2]Napoli, [3]Padova)

18.35 Bvm-c l'anello di congiunzione tra paziente, medico e infermiere
E. Lo Voi *(Assisi, PG)*

18.42 Impatto di un assistente basato su AI sulla qualità dei Piani di Assistenza Individualizzati nel paziente in trattamento sostitutivo renale: studio randomizzato crossover
A. Grimaldi[1], C. Pozzuoli[1], G. Frattolillo[1], D. Di Sivo[2], I. Chiusolo[3], V. Di Nuzzo[1] *([1]Caserta, [2]Napoli, [3]Padova)*

18.49 Il ruolo dell'infermiere esperto in wound care in area nefrologica nella gestione del prurito associato alla malattia renale cronica: uno studio fenomenologico
G. Gavarro[1], D. Di Sivo[2], I. Chiusolo[3], G. Frattolillo[1], C. Pozzuoli1, **V. Di Nuzzo**[1], G. Ferrara[4], A. Grimaldi[1]
([1]Caserta; [2]Napoli; [3]Padova; [4]Carpi, MO)

19.00 **Fine percorso formativo ECM n. 1**

MARTEDÌ 5 MAGGIO 2026

Sala Parigi — **Proseguimento percorso formativo ECM n. 2**

14.00<br>17.30	**ACCESSI VASCOLARI: COMPETENZE AVANZATE, VALUTAZIONE CLINICA E DECISION MAKING INFERMIERISTICO**<br>*Moderatori:* **Mara Canzi**, **Vincenzo Faraone**
14.00	Scelta e pianificazione dell'accesso vascolare nel paziente nefropatico<br>**Silvia Corti**
14.20	Sorveglianza clinica dell'accesso: identificazione precoce dei segni di disfunzione<br>**Antonia Bua**, **Giuseppe Fenoglio**
14.40	Ecografia bedside dell'accesso: competenze infermieristiche avanzate<br>**Rossella Esposto**, **Giuseppe Fenoglio**
15.00	Ricircolo e performance dell'accesso: valutazione clinica e strumenti di monitoraggio<br>**Rossella Esposto**
15.20	Dibattito
15.30	Cannulazione FAV: criteri di scelta, tecniche e gestione clinica<br>**Rossella Esposto**, **Giuseppe Fenoglio**, **Giovanni Grasso**
16.00	Catetere Venoso Centrale: sicurezza, gestione e innovazione<br>**Rossella Esposto**
16.20	Dal protocollo alla pratica clinica: medicazioni e lock, per la prevenzione delle complicanze del CVC<br>**Elena Barile**, **Silvia Cappelletti**
16.40	Gestione avanzata delle disfunzioni del CVC: occlusioni, trombolisi e rimozione sicura dei residui adesivi<br>**Silvia Cappelletti**, **Rossella Esposto**
17.00	Dimensione emotiva e aderenza terapeutica: strategie infermieristiche per la gestione dell'ansia e della tripanofobia<br>**Angelo Guarascio**
17.20	Dibattito e take home message

17.30
18.00 ***Pausa con degustazione alimenti aproteici*** ***in collaborazione con Dr. Schär***

18.00
19.00 **Sessione di Comunicazioni Orali ACCESSI VASCOLARI**

***Moderatori:* Lea Godino, Addolorata Palmisano** *(n. 7 comunicazioni da 7 minuti cad.)*

18.00	Il fonendoscopio elettronico: una nuova frontiera per il monitoraggio dalla fistola artero venosa (F.A.V.) In dialisi<br>**G. Petruzzelli**[1], L. Di Leo[2], G. Petruzzelli[3], T. Piccolo *([1]Minervino Murge, BT; [2]Bisceglie, BT; [3]Barletta)*
18.07	Oltre la medicazione: qual è l'impatto della visione del Catetere Venoso Centrale nel soggetto emodializzato?<br>**S. Barcaro**[1], M. Mettifogo[1], L. Gattera[2] *([1]Vicenza; [2]Valdagno, VI)*
18.14	Assistenza e gestione del paziente sottoposto ad intervento di fav: monitoraggio dell'accesso vascolare nel periodo post-operatorio<br>R. Ciannarella1, G. Petruzzelli1, G. Civetta1, M. L. Biga1, P. V. Pietro1, G. Quacquarelli1, R. Lemma1, R. **Pischetola**[2], G. Petruzzelli[1], T. Piccolo[1] *([1]Barletta, [2]Minervino BT)*
18.21	Il ruolo dell'ecografia infermieristica nella gestione del DVA (difficult vascular access) in area dialitica<br>**R. Lemma**[1], G. Petruzzelli[1], G. Petruzzelli[2], M. L. Biga[1], V. P. Pasquale[1], R. Pischetola[1], G. Quacquarelli[1], T. Piccolo[1] *([1]Barletta; [2]Minervino Murge, BT)*
18.28	Intelligenza Artificiale nel posizionamento ecoguidato dei cateteri venosi centrali per emodialisi e ad alto flusso: revisione sistematica delle evidenze cliniche e tecnologiche (Protocollo AIDIAL-CVC)<br>A. Grimaldi[1], V. Di Nuzzo[1], G. Frattolillo[1], D. Di Sivo[2], I. Chiusolo[3], **C. Pozzuoli**[1] *([1]Caserta, [2]Napoli, [3]Padova)*
18.35	Care bundle infermieristico per la prevenzione della disfunzione dell'accesso vascolare in emodialisi: proposta di progetto pilota<br>**C. Lo Presti**, A. Cassia, G. Dimauro, L. Dimauro, M. Lozito, A.S. Vita, A. Vittorio, M. Correnti, S. Floridia, G. Gianni, L. Renzelli, T. Gianni *(Palazzolo Acreide, SR)*
18.42	CKD-aP in emodialisi: fattibilità e segnale preliminare di efficacia di un protocollo emolliente combinato mirato al ripristino della barriera cutanea nell'ambito di un approccio multimodale<br>**S. Cappelletti** *(San Fermo della Battaglia, CO)*
19.00	**Fine percorso formativo ECM n. 2**

MERCOLEDÌ 6 MAGGIO 2026

Dalla dialisi domiciliare alla nutrizione clinica: due dimensioni centrali del percorso del paziente cronico. La mattina unisce esperienze cliniche, testimonianze e laboratori interattivi per promuovere l'autonomia, la personalizzazione e l'educazione terapeutica.

Sala Londra **Inizio percorso formativo ECM n. 3 e n. 4**

08.30
09.45 **MODELLI PROFESSIONALI EMERGENTI IN NEFROLOGIA: HUMANITIES, DIRITTO E GOVERNANCE CLINICA**
***Moderatori:* Francesco Barci, Alessandro Pizzo**

08.30 Quando le parole diventano cura: narrazione e counselling per sostenere gli infermieri nella complessità assistenziale
Lucia Benetti

09.00 Protocollo di ricerca basato sulle medical humanities per lo sviluppo delle conoscenze giuridiche degli infermieri di area nefrologica
Immacolata De Simone, Stefania Meo, Marisa Pegoraro

09.30 Prevenire, curare, innovare: visione e responsabilità dell'infermiere di area nefrologica
Francesco Barci, Cappelletti Silvia, Domenica Gazineo, Angela Greco, Stefano Mancin, Addolorata Palmisano

Proseguimento percorso formativo ECM n. 3

09.45
10.45 **Sessione di Comunicazioni Orali MISCELLANEA**
***Moderatori:* Domenica Gazineo, Stefano Mancin** *(n. 8 comunicazioni da 7 minuti cad.)*

09.45 Effetti dell'implementazione del modello Primary Nursing sugli esiti assistenziali e organizzativi nell'AUSL Piacenza: studio quantitativo pre-post
A. Contini, R. Barbieri, M. Muroni, M. G. Droghi, L. Cappucciati, M. Maserati, E. Cordani, C. Scaglia, G. Gallo *(Piacenza)*

09.52 Fatigue post-dialisi nella popolazione adulta: analisi dei fattori predittivi e del suo impatto sulla qualità di vita. Studio osservazionale cross-sectional
G. F. Caruso, M. Figura, R. Latina *(Palermo)*

09.59 Implementazione di una strategia educativa gamificata per la prevenzione renale nell'assistenza sanitaria territoriale: Studio PLAY-KID (Preventive Learning Activity for Kidney Injury Detection)
V. Di Nuzzo[1], G. Frattolillo[1], C. Pozzuoli[1], I. Chiusolo[2], D. Di Sivo[3], A. Grimaldi[1] *([1]Caserta, [2]Padova, [3]Napoli)*

10.06 Gli infermieri abruzzesi di nefrodialisi e le cure palliative
G. La Selva[1], V. Caponnetto[2] *([1]Pescara, [2]L'Aquila)*

10.13	Ascoltare la cronicità: la medicina narrativa nella relazione infermiere–paziente in emodialisi<br>**F. Zullino** *(Chieti)*
10.20	Laboratorio di "Penso Scrittura": il fine vita, vissuto e percezione degli infermieri in area Nefrologica<br>**I. De Simone**, M. Pegoraro, S. Meo *(Milano)*
10.27	La scelta di donare: gli eroi del dono nascono a scuola<br>**V. Lacanale**, M. R. Barattucci *(Vasto, CH)*
10.35	Fragilità percepita e qualità di vita nei riceventi di trapianto renale: una revisione sistematica<br>**D. Di Sivo**[1], A. Grimaldi[2], V. Di Nuzzo[2], G. Frattolillo[2], C. Pozzuoli[2], G. Gavarro[2], I. Chiusolo[3] *([1]Napoli, [2]Caserta, [3]Padova)*
10.45<br>13.30	**DIALISI PERITONEALE**<br>***Moderatori:*** **Cinzia Fabbri**, **Paolo Lentini**
10.45	L'integrazione della dialisi peritoneale nei modelli di assistenza sanitaria territoriale<br>**Paolo Lentini**
11.05	Pre-dialisi: ruolo del professionista tra gli attori del MaReA<br>**Francesco Maisto**
11.25	Il Totem: l'evoluzione della telemedicina in ambito della DP<br>**Donatella Lenzi**, **Luca Lovison**
11.45	L'intelligenza artificiale: quali implicazioni per la DP<br>**Anna Dalprà**, **Cristina Mechini**
12.05	Umanizzazione o disumanizzazione? Quale impatto sociale e psicologico ha tutta questa innovazione tecnologica su pazienti e familiari? È salutare o distruttiva?<br>**Anna Dalprà**, **Cristina Mechini**
12.25	La dialisi peritoneale: un trattamento riservato a pochi?<br>**Walter Longo**
12.45	Dibattito
13.30	**Fine percorso formativo ECM n. 3**
13.30	**Chiusura del Congresso e premiazioni**

Sala Parigi **Proseguimento percorso formativo ECM n. 4**

09.45 – 10.45 Sessione di Comunicazioni Orali NUTRIZIONE NEFROLOGICA

***Moderatori:* Silvia Cappelletti, Marco Sguanci** *(n. 8 comunicazioni da 7 minuti cad.)*

09.45 Formaggio FriP: la miglior tradizione casearia al servizio del paziente nefropatico
G. Ardissino, D. Rossetti, G. Tamburini, L. Dato, M.C. Mancuso *(Milano)*

09.52 PHOS-BALANCE-QI: Quality improvement di un intervento educativo strutturato sull'iperfosfatemia nel paziente in emodialisi
I. Chiusolo[1], C. Pozzuoli[2], G. Frattolillo[2], D. Di Sivo[2], V. Di Nuzzo[3], G. Ferrara[4], A. Grimaldi[2]
([1]Padova; [2]Caserta; [3]Napoli; [4]Carpi, MO)

09.59 Aderenza terapeutica e self-management nei pazienti sottoposti a trapianto renale: revisione sistematica di studi randomizzati controllati
D. Di Sivo[1], A. Grimaldi[2], I. Chiusolo[3], G. Frattolillo[2], C. Pozzuoli[2], G. Gavarro[2], V. Di Nuzzo[2]
([1]Napoli, [2]Caserta, [3]Padova)

10.06 Nursing case management e coordinamento delle cure nella valutazione del donatore vivente di rene: una scoping review
A. Grimaldi[1], C. Pozzuoli[1], G. Frattolillo[1], D. Di Sivo[2], V. Di Nuzzo[1], **I. Chiusolo**[3] *([1]Caserta, [2]Napoli, [3]Padova)*

10.13 Digital self-care nei pazienti con malattia renale cronica: uno studio cross-sectional.
F. Pastore[1], S. Cannone[1], L. Scattarelli[1], B. De Luca[1], G. Pannone[1], B. Forastefano[2], G. Cicolini[3], V. Simonetti[3], D. Comparcini[1] *([1]Bari, [2]Roma, [3]Chieti)*

10.20 La qualità di vita del paziente con prurito uremico sottoposto ad emodialisi
S. La Legname[1], V. Giordano[1], C. Palazzo[1], M. Virgolesi[1], F. Ricciardi[1], A. Caputo[2], R. Nocerino[1], M. Strazzulli[1], A. Guillari[1], T. Rea[1] *([1]Napoli, [2]Salerno)*

10.27 Cure di prossimità e CKD in pre-dialisi: proposta di implementazione dell'Infermiere di Comunità per l'intercettazione precoce e la presa in carico territoriale
A. Pirreco[1], G. Gallo[2], M. Gigliello[3], E. Acquistapane[4], V. Caru[5], R. Consoli[6], F. Sacchini[7,9], G. Cangelosi[8], D. Ivzik[1], D. Tartaglini[1] *([1]Roma; 2Piacenza; [3]Salerno; [4]Como; [5]Varese; [6]Milano; [7]Fermo, FM; [8]Camerino, MC)*

10.35 Studio della calcolosi delle vie urinarie nella popolazione dell'Ast di Ascoli Piceno
T. Traini, L. F. Celani, R. Polci *(Ascoli Piceno)*

10.45 / 13.30	**NUTRIZIONE NEFROLOGICA: DALLA TEORIA ALL'AZIONE**<br>*Moderatori:* **Angela Greco, Stefano Mancin**
10.45	Perché la nutrizione è "care" nefrologico<br>**Elena Sandri**
11.05	Dieta su misura in nefrologia: come cambiano i target nutrizionali per stadio della malattia<br>**Stefano Mancin**
11.25	L'infermiere come "key player" della gestione nutrizionale nel paziente nefropatico<br>**Sara Morales Palomares**
11.45	Dalla teoria alla pratica: competenze nutrizionali dell'infermiere nefrologico in Italia. Evidenze e prospettive future<br>**Gaetano Ferrara**
12.05	Guida rapida e tascabile alla nutrizione: raccomandazioni essenziali per il paziente in emodialisi<br>**Giada Vrenna**
12.25	Disfagia nella malattia renale cronica: un rischio sottostimato per malnutrizione e fragilità<br>**Sofia Matteucci**
12.45	Nutrizione e qualità di vita dei pazienti nefropatici: esperienze, bisogni e proposte<br>**Stefano Mancin, Sara Morales Palomares**
13.05	Dibattito
13.30	**Fine percorso formativo ECM n. 4**
13.30	**Chiusura del Congresso e premiazioni in sala Londra**

ACCESSI VASCOLARI

ID06 Audit 2025: indicazioni alla puntura ecoguidata della fav: linee guida? Suggerimenti? Analisi del numero di fav sottoposte a puntura ecoguidate nel corso dell'anno. Chi passa alla puntura a «mano libera»? Tempo dedicati e motivi
P. Pellicanò, D. Pazzano, M. Cozzupoli, R. Angelo, A. Caserta, A. Putortì *(Reggio Calabria)*

ID12 Educazione terapeutica infermieristica nel paziente con fistola artero-venosa: sviluppo di un opuscolo informativo
R. Secondini, G. Ferrara, I. De Stasio *(Carpi, MO)*

ID14 L'implementazione del nurse-led-ultrasound nel monitoraggio degli accessi vascolari: una strategia per la prevenzione delle complicanze in emodialisi (case report)
G. Petruzzelli[1], G. Petruzzelli[2], M. L. Biga[1], V. P. Pasquale[1], G. Quacquarelli[1], R. Lemma[1], R. Pischetola[1], T. Piccolo[1] *([1]Barletta, [2]Minervino Murge, BT)*

ID19 Tecnica must (multilple single cannulation technique) nuova avventura per il nostro centro dialisi
L. Principe *(Arezzo)*

ID36 Efficacia della soluzione con PHMB e propilbetaina rispetto alla Clorexidina 2% nella prevenzione del rischio effettivo nel catetere venoso centrale (CVC) da Dialisi
A. Carrara, G. Grasso, C. Gambirasio, L. Buzzi, I. Baragetti *(Cinisello Balsamo, MI)*

ID54 Sorveglianza infermieristica e longevità degli accessi vascolari: esperienza multicentrica con focus su endofav
C. Lo Presti, A. Cassia, G. Dimauro, L. Dimauro, M. Lozito, A.S. Vita, A. Vittorio, M. Correnti, S. Floridia, G. Gianni, L. Renzelli, T. Gianni *(Palazzolo Acreide, SR)*

COUNSELLING E MEDICINA NARRATIVA

ID25 Il sintomo fisico: un portale di accesso alle vere origini del malessere. dedicato agli infermieri
M. Pegoraro *(Milano)*

ID44 Immagini che parlano: il Photovoice come ponte tra esperienza del paziente e cura nefrologica
S. P. Chiarot[1], S. Padrin[2], **A. Palmisano**[2] *([1]Pordenone; [2]Monselice, PD)*

ID67 Riconoscimento precoce del deterioramento clinico nel paziente nefropatico: ruolo dell'infermiere nefrologico e integrazione con sistemi di early warning
C. Fatone[1], M. Cuomo[1], C. Bellotta[1], A. Musica[2], A. Grimaldi[2] *([1]Napoli; [2]Marcianise, CE)*

ELENCO POSTER DIGITALI IN ORDINE DI TEMA

CRRT (terapie sostitutive renali continue)

ID65 Approccio multiprofessionale alla dialisi per acuti in area critica: l'esperienza del Renal Emergency Team dell'Azienda Ospedale Università di Padova
F. Schiavon, L. Cattarin, R. Zanella, N. Celon, F. Nalesso *(Padova)*

ID66 Anticoagulazione nella CRRT: evidenze da revisione integrativa della letteratura e implicazioni clinico-infermieristiche
G. Artiola, V. Pettillo, K. Generoso, F. Sommella, R. Colonna *(Marano di Napoli, NA)*

CURE PALLIATIVE NEFROLOGICHE

ID11 Emodialisi palliativa nel paziente fragile: esperienza di identificazione precoce e cure condivise
G. Bergna, M. G. Venditti, M. D'Amico, M. C. Gambirasio *(Cinisello Balsamo, MI)*

ID43 Assistenza infermieristica, cure palliative e decisioni di fine vita nel paziente con ESRD: una revisione narrativa
V. Paoletto[1], M. Simioni[2], **A. Palmisano**[2], G. Ferrara[3] *([1]Santorso, VI; [2]Monselice, PD; [3]Carpi, MO)*

ID50 Protocollo di ricerca basato sulle Medical Humanities per lo sviluppo delle conoscenze giuridiche degli infermieri in area nefrologica.
S. Meo, I. De Simone, M. Pegoraro *(Milano)*

ID57 Creazione di un percorso personalizzato volto a migliorare la qualità di vita del paziente dializzato end-stage
L. Bardelli, A. Cerutti, M. Lantieri, C. Paviolo, S. Scelsi, A. Talamona, M. Breda, G. De Faveri, A. Staffa *(Varese)*

DIALISI PERITONEALE

ID69 La relazione tra Health Literacy e Qualità di Vita nel paziente in dialisi peritoneale: un protocollo di studio
G. Bellu[1], R. Morandini[3], **G. F. Caruso**[2], R. Latina[2], G. Pucciarelli[3], M. Figura[2] *([1]Cagliari, [2]Palermo, [3]Roma)*

EMODIALISI

ID31 Underreporting del CKD-Associated Pruritus in emodialisi: studio qualitativo e implicazioni per l'assessment infermieristico proattivo
S. Cappelletti *(San Fermo della Battaglia, CO)*

ID42 Esperienza clinica con la vaccinazione anti–virus respiratorio sinciziale in pazienti in trattamento emodialitico: il ruolo chiave dell'infermiere
F. Marinotti, C. Miccoli, G. Scaparrotta, N. Celon, F. Nalesso *(Padova)*

ID48 Continuità assistenziale e personalizzazione delle cure nel paziente in emodialisi: il valore dell'integrazione ospedale–territorio
S. Cennerazzo, M. Ballarini, D. Baraldi, G. Calò, G. Morandi, A. Rossini *(Bologna)*

ID60 Nursing students burden: vissuto degli studenti infermieri in emodialisi e rischio di burnout
S. Zagame[1], **A. Pirreco**[2] *([1]Catania, [2]Roma)*

ID70 Eco-Controlling Dashboard: un sistema strutturato per la gestione Green dei centri dialisi e la riduzione dell'impatto ambientale
A. Pizzo[1], G. Santarcangelo[1], **G. De Pascale**[2] *([1]Napoli, [2]Salerno)*

NUOVE TECNOLOGIE (DIGITALE, AI, TELEMEDICINA, DISPOSITIVI)

ID08 L'assistenza al paziente in Aferesi Terapeutica a doppia filtrazione: una sfida attuale dell'infermiere di Dialisi. L'esperienza dell'ASL CN1
A. Pecoraro, S. Zeri, E. Naso, F. Tomatis, R. Carrera, S. Dubois Chiabrando, C. Mattarozzi, M. Marengo *(Cuneo)*

ID49 L'utilizzo della realtà virtuale (VR) per la riduzione del dolore e dell'ansia nei pazienti sottoposti a emodialisi: revisione narrativa della letteratura
A. Guarascio[1], S. Cavaliere[2], E. Palma[3] *([1]Cosenza; [2]Messina; [3]Rozzano, MI)*

ID51 Assistenza infermieristica e realtà virtuale per il trattamento dello stress e del dolore nel paziente in emodialisi: una revisione della letteratura
F. L. Riato[1], M. Simioni[2], **A. Palmisano**[2] *([1]Vicenza; [2]Monselice, PD)*

ID68 Vascular Remote Care: la nuova frontiera digitale nel monitoraggio per la gestione degli accessi FAV
M. Cuomo[1], C. Fatone[1], C. Bellotta[1], A. Musica[2], A. Grimaldi[2] *([1]Napoli; [2]Marcianise, CE)*

NUTRIZIONE

ID02 Standardizzare le competenze nutrizionali dell'infermiere nefrologico: una scoping review
G. Ferrara[1], S. Morales Palomares[2], D. Cannizzaro[3], S. Matteucci[4], G. Cangelosi[5], A. Palmisano[6], S. Mancin[4], M. Sguanci[7] *([1]Carpi, MO; [2]Rende, CS; [3]Modena, MO; [4]Milano, MI; [5]Camerino, MC; [6]Padova, PD; [7]Genova, GE)*

ID38 Esperienze e pratiche degli infermieri nefrologici nella cura nutrizionale del paziente con malattia renale cronica: uno studio qualitativo
P. Di Fronzo[1], S. Matteucci[2], G. Ferrara[3], A. Grimaldi[4], S. Mancin[1], M. Sguanci[1] *([1]Olbia, OT; [2]Rozzano, MI; [3]Roma; [4]Caserta)*

PUBLIC HEALTH E NEFROLOGIA

ID23 Le esperienze dei pazienti e dei professionisti della salute nel paziente con malattia renale cronica pre-dialitica: una revisione sistemica qualitativa

E. Acquistapace[1], G. Cangelosi[2], V. Carù[3], R. Consoli[4], **G. Gallo**[5], D. Ivziku[6], M. Gigliello[7], F. Sacchini[8], D. Tartaglini[6], A. Pirreco[6] *([1]Como; [2]Camerino, MC; [3]Varese; [4]Milano; [5]Piacenza; [6]Roma; [7]Salerno; 8Fermo, FM)*

ID24 Salute e malattia, o malattia e salute? ...e se avessimo letto la mappa al contrario?

M. Pegoraro *(Milano)*

ID40 Masterclass infermieristica e qualità assistenziale nella terapia sostitutiva renale: valutazione dell'efficacia a follow-up annuale

C. Circognini[1], A. Pirreco[2], S. Bonaventura[1], G. Pecora[1], C. Marcantoni[1], A. Gambera[1], V. Parrinello[1] *([1]Catania, [2]Roma)*

ID45 Cambiamento climatico e salute renale: implicazioni assistenziali e infermieristiche per le persone con patologie renali

E. Acquistapace[1], G. Cangelosi[2], V. Carù[3], **R. Consoli**[4], G. Gallo[5], M. Gigliello[6], A. Pirreco[7], F. Sacchini[8] *([1]Como; [2]Camerino, MC; [3]Varese; [4]Milano; [5]Piacenza; [6]Salerno; [7]Roma; [8]Sant'Elpidio a Mare, FM)*

TRAPIANTO

ID07 Percorso trapiantologico: procedure chiare, piccola equipe e grandi risultati, un modello organizzativo che funziona. L'esperienza dell'ASLCN1

A. Pecoraro, E. Naso, I. Serra, **R. Carrera**, V. Russo, I. Fea, D. Omento, I. Negro, M. Bosio, C. Mattarozzi, M. Marengo *(Cuneo)*

ID37 Ipotermia lieve nei donatori DBD/DNC (34–35 °C) e outcome del trapianto renale: revisione sistematica di studi randomizzati con prospettiva infermieristica. Revisione sistematica di studi randomizzati con prospettiva infermieristica

A. Grimaldi[1], C. Pozzuoli[1], V. Di Nuzzo[1], F. Colella[1], D. Di Sivo[1], I. Chiusolo[3], **G. Frattolillo**[1] *([1]Caserta, [2]Napoli, [3]Padova)*

ID53 Implementazione del Primary Nursing nel monitoraggio clinico continuo dei pazienti candidati a trapianto renale

B. Asbusti, B. Gargiulo, A. Rossini, M. Ballarini, L. Panicali, R. Palumbo, R. Toschi *(Bologna)*

E.C.M.
Commissione Nazionale Formazione Continua

E.C.M. – EDUCAZIONE CONTINUA IN MEDICINA

Il Provider My Meeting n. 1396 ha effettuato l'accreditamento E.C.M. per i seguenti **percorsi formativi RES** accreditati **per n. 200 partecipanti** ciascuno, per le seguenti categorie:

- **INFERMIERE**
- **INFERMIERE PEDIATRICO**
- **DIETISTA**
- **BIOLOGO**
- **MEDICO CHIRURGO** *discipline: medicina e chirurgia di accettazione e di urgenza; medicina interna; nefrologia; chirurgia vascolare; anestesia e rianimazione; medicina generale (medici di famiglia); scienza dell'alimentazione e dietetica; organizzazione dei servizi sanitari di base*

Martedì 5 Maggio 2026

Numero Percorso	Percorso	Crediti ECM	Durata attività formativa	Responsabile Scientifico
**Percorso n. 1**<br>**ID 1396-479808**	Mattino Sala Plenaria +<br>Pomeriggio Sala Londra CRRT	**n. 4,9**	**n. 7 ore**	Francesco Gravante

Obiettivo formativo tecnico-professionale n. 18
Contenuti tecnico-professionali (conoscenze e competenze) specifici di ciascuna professione, di ciascuna specializzazione e di ciascuna attività ultraspecialistica, ivi incluse le malattie rare e la medicina di genere

Numero Percorso	Percorso	Crediti ECM	Durata attività formativa	Responsabile Scientifico
**Percorso n. 2**<br>**ID 1396-479813**	Mattino Sala Plenaria +<br>Pomeriggio Sala Parigi ACCESSI VASCOLARI	**n. 4,9**	**n. 7 ore**	Rossella Esposto

Obiettivo formativo di sistema n. 2
Linee guida - protocolli - procedure

Mercoledì 6 Maggio 2026

Numero Percorso	Percorso	Crediti ECM	Durata attività formativa	Responsabile Scientifico
**Percorso n. 3**<br>**ID 1396-479820**	Mattino Sala Londra +<br>Sala Londra DIALISI PERITONEALE	**n. 3,5**	**n. 5 ore**	Francesco Maisto

Obiettivo formativo di processo n. 8
Integrazione interprofessionale e multiprofessionale, interistituzionale

Numero Percorso	Percorso	Crediti ECM	Durata attività formativa	Responsabile Scientifico
Percorso n. 4<br>**ID 1396-479824**	Mattino Sala Plenaria +<br>Sala Parigi NUTRIZIONE NEFROLOGICA	**n. 3,5**	**n.5 ore**	Sara Morales Palomares

Obiettivo formativo tecnico-professionale n. 23
Sicurezza e igiene alimentari, nutrizione e/o patologie correlate

Il test di apprendimento E.C.M. e il questionario di gradimento dell'evento saranno disponibili online.
Per accedere ai questionari:
1. Collegarsi al sito https://mymeeting.testecm.it oppure scansionare il QR Code
2. Effettuare la registrazione attraverso il proprio codice fiscale
3. Selezionare il corso frequentato nella sezione "eventi in corso"

I questionari SARANNO ATTIVI per 72 ore dalla conclusione di ciascun percorso formativo:
Percorso ECM n. 1 e n. 2 dalle ore 19.00 del giorno 5 maggio alle ore 19.00 del giorno 8 maggio 2026.
Percorso ECM n. 3 e n. 4 dalle ore 13.30 del giorno 6 maggio alle ore 13.30 del giorno 9 maggio 2026.
E' consentito un solo tentativo.

A test concluso il sistema invierà una notifica di avvenuta registrazione delle risposte e l'eventuale comunicazione di non superamento del test. In caso di necessità, durante la compilazione dei questionari, sarà attivo un servizio di assistenza tecnica disponibile esclusivamente tramite WhatsApp ore 9.00-18.00 al numero 375 8228302

L'attestato, conferente i crediti, sarà rilasciato ai partecipanti che avranno frequentato il percorso formativo (90% del monte ore) e risposto correttamente ad almeno il 75% delle domande del test di apprendimento.

NORME PER LA FACULTY

Il Congresso sarà soggetto ad **accreditamento E.C.M.**, richiediamo pertanto il rispetto delle seguenti norme:
- l'attività di docenza dovrà essere obiettiva, equilibrata e non influenzata da interessi diretti o indiretti di soggetti portatori di interessi commerciali;
- il relatore/docente non deve inserire nell'attività didattica pubblicità di qualsiasi tipo per specifici prodotti di interesse sanitario, deve indicare solo nomi generici di farmaci, strumenti, presidi e non il nome commerciale, anche se correlato all'argomento trattato;
- il relatore/docente non deve proiettare immagini coperte da diritto d'autore, immagini identificative di farmaci o apparecchiature e immagini che possano violare la privacy dei pazienti.
- il relatore/docente deve comunicare ai partecipanti, prima dell'inizio della propria relazione/docenza, eventuali fonti di finanziamento o dei rapporti con soggetti portatori di interessi commerciali in ambito sanitario, come dichiarato nell'apposita autocertificazione.

Si invita tutta la faculty a prendere visione dell'Informativa Nazionale Formatori Corsi ECM consultabile sul sito Agenas al seguente link https://ecm.agenas.it/privacy-policy

PROIEZIONI > PREFERIBILMENTE 16:9

La sala sarà provvista di Personal Computer per la presentazione dei lavori con videoproiezione. E' consentito l'uso del proprio PC/MAC previa comunicazione anticipata ai tecnici di sala: dotarsi di proprio adattatore. Le relazioni dovranno essere preparate con Power Point e consegnate almeno un'ora prima dell'inizio della presentazione al tecnico del Centro Slide per verificare la compatibilità del proprio materiale tecnico.
Si ricorda di rispettare rigorosamente il tempo assegnato per ciascun intervento per un corretto svolgimento delle sessioni scientifiche.

COMUNICAZIONI ORALI

Gli autori dei contributi accettati come **COMUNICAZIONI ORALI** avranno a disposizione **5 minuti per l'esposizione + 2 minuti per la discussione**, l'autore dovrà preparare una presentazione in Power Point di **massimo n. 6 slide utilizzando il template fornito da SIAN**. Le slide dovranno essere consegnate almeno un'ora prima dell'inizio della presentazione al tecnico del Centro Slide.

POSTER DIGITALI

I contributi accettati come **POSTER DIGITALI** saranno visibili **su postazioni totem touchscreen verticali**, in cui potranno essere consultati liberamente dai partecipanti per tutta la durata del congresso. Il file in PDF sarà inoltre pubblicato sul sito internet SIAN nella pagina dedicata agli atti del Congresso.

PREMI E RICONOSCIMENTI

Mercoledì 6 maggio 2026 alle ore 13.30 in Sala Londra sono previste le premiazioni degli abstract per le seguenti categorie:

- **Miglior Abstract del Congresso**
- **Miglior Poster**
- **Miglior Presentazione Orale** *(primi 3 classificati)*
- **Menzioni speciali per innovazione, impatto sul paziente, trasferibilità**

I migliori lavori selezionati saranno pubblicati a spese SIAN sulla rivista HEALTHCARE.

SIMULATION LAB — TOCCA. PROVA. DECIDI.

NEW

Un'esperienza pratica pensata per gli infermieri di dialisi:
non solo osservare, ma fare, sperimentare e portare a casa competenze reali.

COS'È

Il Simulation Lab è un percorso formativo sul campo, organizzato in 3 aree tematiche, dove potrai partecipare attivamente in piccoli gruppi e confrontarti con tecnologie, tecniche e gesti clinici.

LE AREE

- **ACCESSO VASCOLARE** Vedere, sentire, decidere *(ecografia e simulazione su braccio)*
- **TECNOLOGIA DIALITICA** Impostare, monitorare, comprendere *(emodialisi, CRRT, dialisi peritoneale)*
- **DETERSIONE AVANZATA** Utilizzo pratico di poliesanide e betaina *(applicazione corretta e standardizzazione della procedura)*

COME FUNZIONA

- Accesso solo su prenotazione
- Esperienza pratica a rotazione nelle 3 aree
- Gruppi a numero limitato *(max 9 partecipanti)*
- Durata totale percorso: circa 45 minuti

Ogni partecipante vivrà tutte le aree del laboratorio

PRENOTAZIONE

La prenotazione si effettua in loco, al momento del ritiro badge, fino ad esaurimento posti, l'accesso è consentito solo agli iscritti prenotati. Ti consigliamo di prenotarti subito per assicurarti uno slot.

PASSPORT

All'ingresso riceverai il tuo Simulation Lab Passport

✔ Ti accompagnerà nel percorso
✔ Verrà timbrato in ogni area
✔ Certificherà la tua partecipazione completa

COSA PORTI A CASA

- Esperienza pratica reale
- Confronto diretto con esperti
- Checklist operativa utilizzabile in reparto

PERCHÉ PARTECIPARE

"Perché in nefrologia non basta sapere. Serve saper fare."

TI ASPETTIAMO AL SIMULATION LAB IN SALA MADRID PIANO -1

ATTESTATO DI PARTECIPAZIONE

L'attestato di partecipazione sarà spedito per e-mail al termine dei lavori scientifici a tutti gli iscritti che avranno ritirato il badge nominativo in Segreteria.

VARIAZIONI

Il Comitato Scientifico e la Segreteria Organizzativa si riservano il diritto di apportare al Programma tutte le variazioni che dovessero essere ritenute necessarie per ragioni tecniche e/o scientifiche nel rispetto del percorso formativo accreditato.

TRATTAMENTO DI IMMAGINI FOTOGRAFICHE E RIPRESE VIDEO

Durante il congresso potrebbero essere effettuate riprese video e fotografiche con l'esclusiva finalità di documentare l'evento. I file prodotti saranno parte integrante dell'archivio storico di My Meeting Srl e del promotore dell'evento e potrebbero essere oggetto di pubblicazione sui canali social e/o siti web istituzionali dei proponenti.

PORTALE SVC

Sistema Valutazione Conferenze CONFINDUSTRIA Dispositivi Medici

L'evento cod. 2026-0309103610 è stato valutato conforme al Codice Etico da parte dell'Ufficio Compliance

PRIVACY

Ai sensi del Regolamento EU 2016/679 (GDPR) del Parlamento Europeo e del Consiglio del 27 aprile 2016 e del D.Lsg 30/06/2003 n° 196 relativo alla protezione delle persone fisiche con riguardo al trattamento dei dati personali, nonché alla libera circolazione di tali dati.

Soggetto titolare del trattamento dei dati: My Meeting Srl nella persona del Legale Rappresentante Dott.ssa Cristina Federici – Via 1° Maggio 33/35 – 40064 Ozzano dell'Emilia (BO) Bologna – C. F. / P. IVA 02759781202 – Tel 051 796971 – e-mail: info@mymeetingsrl.com

Finalità del Trattamento: i dati fornitici nella presente occasione saranno oggetto di trattamento informatico e manuale, al fine dare seguito alla richiesta e di documentare la Sua partecipazione all'evento e a trattamenti derivanti da obblighi di legge. I dati saranno comunicati a fornitori e soggetti diversi che concorrono o partecipano a vario titolo all'evento e alle competenti Autorità secondo le prescrizioni di Legge ed eventualmente ad AGENAS ai fini ECM. I dati saranno altresì utilizzati per l'invio della Newsletter periodica che la manterrà aggiornato sugli eventi di suo interesse organizzati da My Meeting.

Conservazione dei dati personali: My Meeting Srl conserverà i suoi dati per il tempo necessario alla gestione delle attività connesse sopra indicate, evitando in ogni caso la conservazione a tempo indeterminato e favorendo l'esercizio dei propri diritti da parte dell'interessato.
Il tempo di conservazione è fissato in cinque anni, salvo che l'interessato esegui azioni che confermino la sua volontà di consentire l'utilizzo dei suoi dati.
La compilazione della "Scheda di Iscrizione" costituisce presa d'atto della presente Informativa e dei diritti di cui sopra e consente il trattamento dei dati personali e la loro comunicazione per le finalità sopra indicate. Il mancato consenso le precluderà la partecipazione all'evento.

SEDE CONGRESSUALE

ZANHOTEL EUROPA ★★★★
Via Cesare Boldini, 11
40121 Bologna
Tel. 051 4211348
www.zanhotel.it/hotel-europa-bologna
hoteleuropa@zanhotel.it

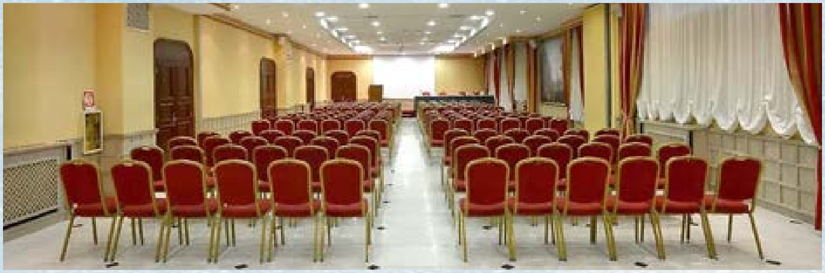

ISCRIZIONI

La partecipazione al Congresso è pagamento. Per iscriversi è necessario compilare il modulo di iscrizione online" All'atto di iscrizione è necessario esprimere la preferenza del percorso formativo scelto, le adesioni saranno accolte fino al raggiungimento dei posti disponibili.

QUOTE DI ISCRIZIONE 44° CONGRESSO NAZIONALE SIAN

dal 31/3/2026 e in sede

	IVA inclusa	*IVA esclusa*
Soci SIAN	**€ 260,00**	**€ 213,11**
NON Soci SIAN	**€ 310,00**	**€ 254,10**

L'iscrizione al 44° Congresso Nazionale comprende:
- *la partecipazione alle Sessioni Scientifiche*
- *il materiale congressuale*
- *la ristorazione prevista da programma*
- *l'attestato di partecipazione*

APERITIVO DI BENVENUTO

L'aperitivo di Benvenuto sarà offerto da SIAN a tutti i partecipanti regolarmente iscritti al Congresso.
Si svolgerà **lunedì 4 maggio 2026 alle ore 19.00 in sede congressuale**, al termine dell'Assemblea Annuale Ordinaria SOCI.

CENA SOCIALE

Martedì 5 maggio alle ore 20.30 si svolgerà la Cena Sociale SIAN presso il **Ristorante Bravo Caffè** *(Via Mascarella, 1 - 40126 Bologna BO)* walking distance dalla sede congressuale. **Partecipazione su prenotazione al costo di Euro 30,00 per persona.** La location ha una capienza limitata pertanto eventuali richieste pervenute in sede congressuale saranno evase in base alla disponibilità dei posti.

ASSEMBLEA ANNUALE ORDINARIA SOCI

Si terrà in Sala Plenaria **lunedì 4 maggio 2026 dalle ore 18.15 alle ore 19.00**.

PRENOTAZIONI ALBERGHIERE

In occasione del Congresso è stata attivata una convenzione per i pernottamenti in **Sede Congressuale ZanHotel Europa**★★★★
Via Cesare Boldrini, 11 | 40121 Bologna | Tel. 051 4211348
www.zanhotel.it/hoteleuropa-bologna hoteleuropa@zanhotel.it

Camera DUS Doppia Uso Singola	*Camera DOPPIA*
**€ 130,00**	**€ 150,00**

Tariffe per camera, per notte, comprensive di prima colazione e IVA 10%. Tassa di soggiorno esclusa.

Lunedì 4 maggio 2026

	Sala Plenaria	Sala Madrid *(piano -1)*	Area Poster
08.30	*Registrazione dei partecipanti Pre-Congresso*		
09.15<br>12.30	**Sessione Pre-Congresso**<br>**Formazione, ricerca e pratica clinica: un dialogo per l'evoluzione dell'assistenza infermieristica nefrologica**		
13.30	*Registrazione dei partecipanti Congresso*		
14.00	**Saluti introduttivi e Apertura dei lavori**<br>**Lettura Magistrale**	**SIMULATION LAB**	**ESPOSIZIONE POSTER DIGITALI**
14.40<br>15.30	**Tavola Rotonda**<br>POSITION PAPER SIAN COMPETENZE INFERMIERISTICHE IN NEFROLOGIA E DIALISI		
15.30<br>16.15	**I Sessione**<br>PREVENZIONE E PRESA IN CARICO PROATTIVA NEL PAZIENTE NEFROPATICO: MODELLI ORGANIZZATIVI E IMPATTO DI SANITÀ PUBBLICA		
16:15<br>16.30	***Coffee break***		
16.30<br>17.15	**II Sessione**<br>CKD-aP: OLTRE IL SINTOMO. NUOVE PROSPETTIVE ASSISTENZIALI, CLINICHE E MULTIDISCIPLINARI		
17.15<br>18.00	**III Sessione**<br>IL TRAPIANTO PRE-EMPTIVE DA DONATORE VIVENTE E DA DONATORE DECEDUTO: FOCUS SULL' ATTIVITÀ INFERMIERISTICA NELL'AMBULATORIO MaReA IN ITALIA		
18.15<br>19.00	Assemblea Annuale Ordinaria SOCI SIAN		

Martedì 5 maggio 2026

	Sala Plenaria	Sala Madrid *(piano -1)*	Area Poster
08.30<br>09.45	PERCORSO ECM 1 e 2<br>**Tavola Rotonda**<br>GREEN DIALYSIS: SOSTENIBILITÀ, SICUREZZA E NUOVI MODELLI ASSISTENZIALI NEL PAZIENTE NEFROPATICO	**SIMULATION LAB**	**ESPOSIZIONE POSTER DIGITALI**
10.45<br>11.00	***Coffee break***		
11.00<br>13.00	**Simposi aziendali**		
13.00<br>14.00	***Light Lunch***		

	Sala Londra	Sala Parigi	Sala Madrid *(piano -1)*	Area Poster
14.00<br>17.30	PERCORSO ECM 1<br>CRRT OGGI: CLINICA, FORMAZIONE E RICERCA PER UNA GESTIONE INFERMIERISTICA	PERCORSO ECM 2<br>ACCESSI VASCOLARI: COMPETENZE AVANZATE, VALUTAZIONE CLINICA E DECISION MAKING INFERMIERISTICO	**SIMULATION LAB**	**ESPOSIZIONE POSTER DIGITALI**
17.30<br>18.00	*pausa lavori*	*pausa lavori*		
18.00<br>19.00	**Sessione di Comunicazioni Orali**<br>CRRT e Ricerca infermieristica	**Sessione di Comunicazioni Orali**<br>ACCESSI VASCOLARI		

Mercoledì 6 maggio 2026

	Sala Londra	Sala Parigi	Sala Madrid *(piano -1)*	Area Poster
08.30<br>09.45	PERCORSO ECM 3 e 4<br>MODELLI PROFESSIONALI EMERGENTI IN NEFROLOGIA: HUMANITIES, DIRITTO E GOVERNANCE CLINICA		**SIMULATION LAB**	**ESPOSIZIONE POSTER DIGITALI**
09.45<br>10.45	PERCORSO ECM 3<br>**Sessione di Comunicazioni Orali**<br>MISCELLANEA	PERCORSO ECM 4<br>**Sessione di Comunicazioni Orali**<br>NUTRIZIONE NEFROLOGICA		
10.45<br>13.30	DIALISI PERITONEALE	NUTRIZIONE NEFROLOGICA: DALLA TEORIA ALL'AZIONE		
13.30	Chiusura del Congresso e premiazioni			

Si ringraziano per il contributo non condizionante dato all'evento

Platinum Sponsor

B BRAUN SHARING EXPERTISE

NIPRO

Vantive

Gold Sponsor

FRESENIUS MEDICAL CARE

GSK

SAPIO life

e inoltre

Abbott

Ceramol

CSL

DANONE | NUTRICIA

Dr Schär INNOVATING SPECIAL NUTRITION

EMODIAL

ERREKAPPA EUROTERAPICI

FRIP

neupharma

NUTRISENS insieme, diamo più gusto alla vita

PHARMANUTRA

SVAS BIOSANA

UNIFARCO we care for care

Elenco aggiornato al 22-04-2026

www.sianitalia.it

My Meeting

PCO e Provider ECM n. 1396

My Meeting S.r.l.

Via I° Maggio 33/35 | 40064 Ozzano dell'Emilia (BO), Italy | Tel. e WhatsApp Business 051 796971

siansegreteria@mymeetingsrl.com | www.mymeetingsrl.com

U1 - stampato Aprile 2026

In copertina "Le due torri di Bologna" si ringrazia per la gentile concessione Maria Grazia Landini
